# BRD4 inhibition suppresses histone H4 UFMylation to increase ferroptosis sensitivity through TXNIP

**DOI:** 10.1038/s41419-025-08166-y

**Published:** 2025-11-17

**Authors:** Zhen Yang, Baoshuai Wang, Bin Guo, Shimin Sun, Yishen Li, Jingbo Lu, Xuejun Cao, Hao Wang, Xinyu Wang, Yongjian Guo, Tao Wu

**Affiliations:** 1 State Key Laboratory of Natural Medicines, Jiangsu Key Laboratory of Carcinogenesis and Intervention, School of Basic Medicine and Clinical Pharmacy, China Pharmaceutical University, Nanjing, People’s Republic of China; 2 School of Biopharmacy, China Pharmaceutical University Jiangning Campus, Nanjing, People’s Republic of China

**Keywords:** Oncogenes, Epigenetics

## Abstract

Bromodomain Containing 4 (BRD4) inhibition selectively alters gene transcription, which subsequently influences cellular responses to BET inhibitors. The specific genes that mediate the effects of BET inhibitors in solid tumors remain inadequately characterized. In this study, we demonstrate that the BET inhibitor JQ1 induces the upregulation of Thioredoxin Interacting Protein (TXNIP), which mediates the anti-tumor effects of JQ1. Mechanistically, JQ1 reduces histone H3 Lysine 9 trimethylation within *TXNIP* promoter, enhancing its transcription in the presence of glucose. Increased TXNIP inhibits histone H4 UFMylation by disrupting the interaction between H4 and UFM1 binding protein 1 (UFBP1), a pivotal component mediating protein UFMylation. Rather than modulating cMYC expression directly, H4 UFMylation facilitates the chromatin binding of cMYC to promote the transcription of cell cycle regulatory genes. Furthermore, TXNIP inhibits the proteasomal degradation of P27, a cyclin-dependent kinase (CDK) inhibitor. Consequently, solid cancer cells treated with JQ1 enter a dormant state which is associated with cancer relapse and drug tolerance. Nevertheless, these quiescent cells exhibit sensitivity to ferroptosis, suggesting that BET inhibitors enhance the anti-tumor efficacy of ferroptosis inducers. Collectively, our findings elucidate the regulators of protein UFMylation and cMYC activity, which modulate cellular responses to BET inhibitors and ferroptosis inducers in solid cancer cells.

## Introduction

BRD4, a member of the Bromo and Extra-Terminal (BET) family, represents a promising target for cancer treatment. Its inhibitors have been continuously discovered and shown significant anti-tumor efficacy in preclinical studies, primarily through the induction of cancer cell cycle arrest and cell death. However, the clinical application of these agents has been hindered by side effects and the development of tumor resistance [1–4].

The transcriptional outcomes of BRD4 inhibition determine the sensitivity of cancer cells to BET inhibitors [5, 6]. Given that BRD4 binds to acetylated histones and facilitates gene transcription and mRNA splicing [1–3], it is not unexpected that inhibiting BRD4 leads to the repression of oncogenes such as cMYC. However, BRD4 inhibition also results in the upregulation of various genes [7–13], and the potential role of these upregulated genes in mediating cancer cell responses to BET inhibitors has been largely overlooked. A comprehensive understanding of the transcriptional alterations induced by BRD4 inhibition could elucidate the anti-tumor mechanisms of BET inhibitors and improve the efficacy of BRD4-targeted therapies in cancer treatment [5, 8, 9].

Protein UFMylation is a ubiquitin-like post-translational modification that, similar to ubiquitination, is mediated by a cascade of enzymes, including UBA5, UFC1, and UFL1, which ultimately transfer one or multiple UFM1 proteins to target substrates. Currently, only a limited number of proteins have been identified as UFMylated, including UFBP1, ASC1, RPL26, P53, histone H4, MRE11, and PD-L1. The UFMylation of these proteins regulates endoplasmic reticulum (ER) homeostasis, ribosome-associated quality control, unfolded protein response (UPR), gene transcription, and DNA damage response. Despite these critical roles of protein UFMylation in various cellular processes, the mechanisms governing its regulation within cells remain poorly understood [14, 15].

Ferroptosis is a unique type of cell death characterized by iron-dependent lipid peroxidation. Ferroptosis-inducing therapies hold promise for cancer treatment, since therapy-resistant cancer cells, which contribute to cancer relapse and drug tolerance, exhibit heightened susceptibility to ferroptosis [16–18]. It is well established that excessive reactive oxygen species (ROS) and iron overload trigger ferroptosis, while glutathione (GSH), GPX4, FSP1, and DHODH protect cells against ferroptosis. Whether BRD4 and protein UFMylation are involved in ferroptosis are largely unexplored.

In this study, we demonstrated that BRD4 negatively regulates the expression of TXNIP. Increased TXNIP upon BRD4 inhibition not only sustained P27 expression but also suppressed H4 UFMylation, which facilitated cMYC binding to its target genes in solid tumor cells. As a result, cancer cells treated with BET inhibitors experienced cell cycle arrest and exhibited increased sensitivity to ferroptosis inducers. This study reveals a new way through which BET inhibitors suppresses tumor cell growth and provides a rationale for targeting BRD4 and UFMylation to enhance ferroptosis in solid tumors. Additionally, these findings offer insights into the regulatory mechanisms governing H4 UFMylation and cMYC transcriptional activity.

## Results

### BRD4 inhibition increased TXNIP expression and resulted in cell cycle arrest

**Fig. 1:**
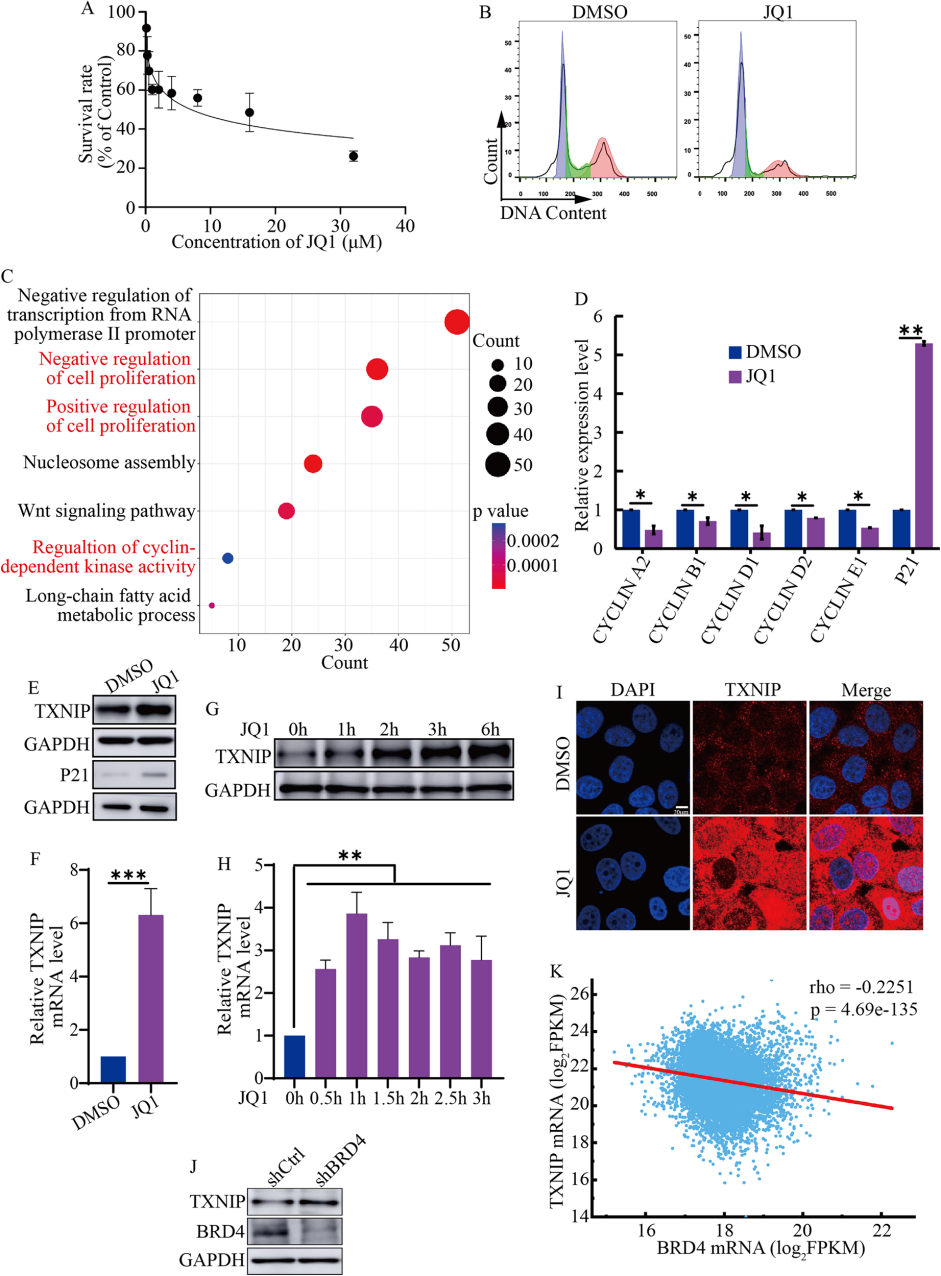
BET inhibitor caused cell cycle arrest and upregulated TXNIP. **A** The survival rate of HepG2 cells treated with different concentrations of BET inhibitor JQ1. **B** Flow cytometric analysis of the cell cycle in HepG2 cells treated with 2 μM JQ1 for 48 h. **C** Gene Ontology (GO) analysis of differentially expressed genes following treatment with 2 μM JQ1. **D** RT-qPCR analysis of the transcription levels of *CYCLIN A2*, *B1*, *D1*, *D2*, *E1*, and *P21*. **E** The protein levels of TXNIP and P21 in control cells versus those treated with 2 μM JQ1. **F** RT-qPCR analysis of TXNIP transcription after treatment with 2 μM JQ1. **G**, **H** The changes of TXNIP protein (**G**) and mRNA (**H**) levels over time with JQ1 treatment. Cells were treated with 2 μM JQ1 for indicated time periods. **I** Immunostaining of TXNIP in HepG2 cells treated with DMSO and 2 μM JQ1 for 48 h. Scale bar: 20 μm. **J** TXNIP expression levels after BRD4 knockdown using shRNA. **K** The correlation between TXNIP and BRD4 expression. Pearson correlation test was used to evaluate the association between TXNIP and BRD4 mRNA levels from TCGA pan-cancer database. ***p value < 0.005, **p value < 0.01, *p value < 0.05 (two-tailed unpaired Student *t* test in **D**, **F**, and **H**).

To investigate the impact of BET inhibitors on solid tumor growth, we initially treated hepatocellular carcinoma HepG2 cells with varying concentrations of the BET inhibitor JQ1. Our results indicated that JQ1 inhibited cell proliferation in a dose-dependent manner (Fig. 1A). Similar inhibitory effects were observed in two additional liver cancer cell lines, SMMC-7721 and Hepa 1–6 (Fig. S1A, B). Notably, rather than inducing cell death (Fig. S1C), JQ1 primarily caused cell cycle arrest in HepG2 cells following 48 h of treatment (Fig. 1B). We performed a global transcriptomic profiling (RNA-seq) in DMSO and JQ1 treated cells and found significant alterations in the expression of genes associated with cell proliferation and cyclin-dependent kinase (CDK) activity (Fig. 1C). Consistent with this result, real time-PCR (RT-qPCR) analysis showed that JQ1 inhibited the transcription of several cyclins, including *CYCLIN A2*, *B1*, *D1*, *D2* and *E1* (Fig. 1D), while concurrently activating P21, an inhibitor of CDKs (Fig. 1D, E).

Given that BRD4 is typically regarded as a transcriptional activator, it was noteworthy that our RNA-Seq data revealed an upregulation of various genes following JQ1 treatment (Fig. S1D). Gene Ontology (GO) analysis indicated that these upregulated genes were implicated in CDK inhibitor activity (Fig. S1E). We sought to determine whether any of these genes mediated the effects of JQ1. Among the identified genes, TXNIP, a tumor suppressor, was greatly induced by JQ1. Western blotting and RT-qPCR results confirmed the upregulation of TXNIP (Fig. 1E, F). Furthermore, *TXNIP* transcription was activated in cells treated with JQ1 for just thirty minutes, and its protein levels increased after two hours of treatment (Fig. 1G, H). These observations suggested that the elevation of TXNIP expression was less likely to be a mere secondary effect of JQ1 treatment. The increased TXNIP localized in both the cytoplasm and nucleus (Fig. 1I). Additionally, JQ1 enhanced TXNIP expression across all examined solid cancer cell lines, including MHCC-97H, SMMC-7721, Hepa 1–6, A375, Capan-2, and HCT116 (Fig. S1F), suggesting that the induction of TXNIP expression by BET inhibitors was a common phenomenon in solid cancer cells.

While JQ1 was prone to inhibit BRD4, it also demonstrated the capacity to inhibit BRD2 and BRD3. The expression of TXNIP was upregulated following the knockdown of BRD4 with shRNA (Fig. 1J). In contrast, the knockdown of BRD2 and BRD3 did not influence TXNIP expression (Fig. S1G, H). This inverse association between TXNIP and BRD4 expression was further corroborated through an analysis of the TCGA pan-cancer database (Fig. 1K). These data suggested that BRD4 repressed TXNIP expression in cancer cells.

### TXNIP mediated the effects of JQ1 on cell cycle

**Fig. 2:**
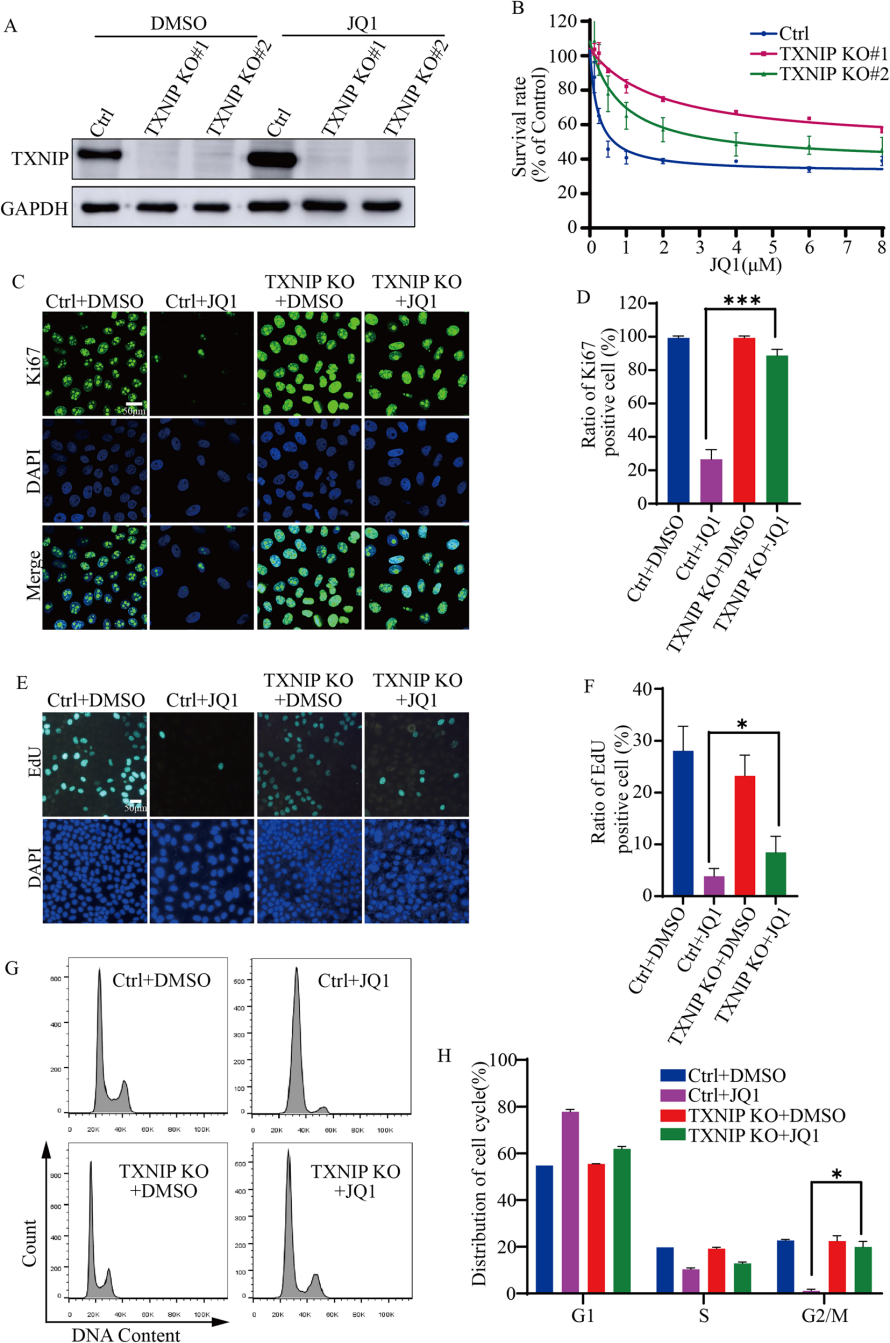
Increased TXNIP resulted in cell cycle arrest upon JQ1 treatment. **A** TXNIP expression in Control (Ctrl) and two TXNIP knockout (KO) cell lines. *TXNIP* in HepG2 cells was knocked out by CRISPR-Cas9. **B** Survival rates of Control and TXNIP KO cells treated with different concentration of BET inhibitor JQ1. **C** Ki67 immunostaining in Control and TXNIP KO cells treated with DMSO and 2 μM JQ1 for 48 h. Scale bar: 50 μm. **D** The proportion of Ki67 positive cells in Control versus TXNIP KO cells, quantified from (**C**). **E** EdU labeling in Control and TXNIP KO cells treated with DMSO and 2 μM JQ1 for 48 h. Scale bar: 50 μm. **F** The proportion of EdU positive cells in Control and TXNIP KO cells, quantified from (**E**). **G** Cell cycle analysis of Control and TXNIP KO cells treated with 2 μM JQ1 for 48 h by flow cytometry. **H** The distribution of cell cycle. Quantification of (**G**). ***p value < 0.005, *p value < 0.05 (one-way ANOVA test in **D**, **F**, and **H**).

The expression level of TXNIP was lower in primary tumor tissues than that in normal solid tissues (Fig. S2A), and low TXNIP expression was associated with poor prognostic outcomes in liver cancer patients (Fig. S2B), suggesting a potential role for TXNIP in suppressing liver cancer growth. To understand whether TXNIP mediated the anti-tumor effects of JQ1, we knocked out TXNIP in HepG2 cells with two sgRNA and subsequently assessed the sensitivity of these cells to JQ1. The results indicated that both TXNIP knockout (KO) cells became less sensitive to JQ1 in comparison to control cells (Fig. 2A, B).

Given that JQ1 induced cell growth inhibition, we investigated whether increased TXNIP was implicated in JQ1-caused cell cycle arrest. JQ1 significantly decreased the number of Ki67 positive cell, with a minimal effect on TXNIP KO cells (Fig. 2C, D). Correspondingly, JQ1 treatment resulted in a reduction of Ki67 protein levels, and the trend was reversed in TXNIP KO cells as well (Fig. S3A). Similar results were observed by labeling proliferating cell with EdU (Fig. 2E, F). Flow cytometric analysis of the cell cycle revealed that control cells were arrested in the G1 phase following JQ1 treatment, whereas a greater proportion of TXNIP KO cells progressed to the G2/M phase (Fig. 2G, H). These results suggested that JQ1 increased TXNIP, which in turn impeded cell cycle progression.

TXNIP has miscellaneous activities in cell, including binding to and recycling the glucose transporter GLUT1 from the cell membrane, thereby inhibiting glucose uptake [19]. We examined whether BET inhibitor could reduce glucose uptake by increasing TXNIP. While JQ1 did reduce membrane localized GLUT1 (Fig. S3B), it did not significantly affect glucose uptake in the cells (Fig. S3B). Moreover, TXNIP KO did not change glucose uptake either (Fig. S3C). Consistently, JQ1 did not regulate lactate production (Fig. S3D). While ATP production was elevated by JQ1, this increase was not mitigated by TXNIP KO (Fig. S3E). Collectively, these findings suggested that although TXNIP reduced membranous GLUT1, it was not important in controlling glucose uptake and metabolism in response to JQ1 treatment, at least in HepG2 cells.

It has also been documented that BRD4 inhibition enhanced the transcription of lysosomal and autophagic genes, and TXNIP also activated autophagy [20]. However, JQ1 promoted lysosome formation in both control and TXNIP KO cells (Fig. S3F). IL1β was increased when inflammasome was activated by TXNIP [21, 22]. Our results showed that IL1β was not changed by JQ1 or TXNIP KO, suggesting that JQ1-induced TXNIP did not activate inflammasome in HepG2 (Fig. S3G). Taken together, while TXNIP regulated glucose metabolism, autophagy, and inflammasome activation, the TXNIP induced by BET inhibitors was not involved in these processes in HepG2 cells. Instead, it primarily led to cell cycle arrest in response to BET inhibitor treatment in solid cancer cells.

### JQ1 inhibited histone H3K9 trimethylation to increase TXNIP expression in the presence of glucose

**Fig. 3:**
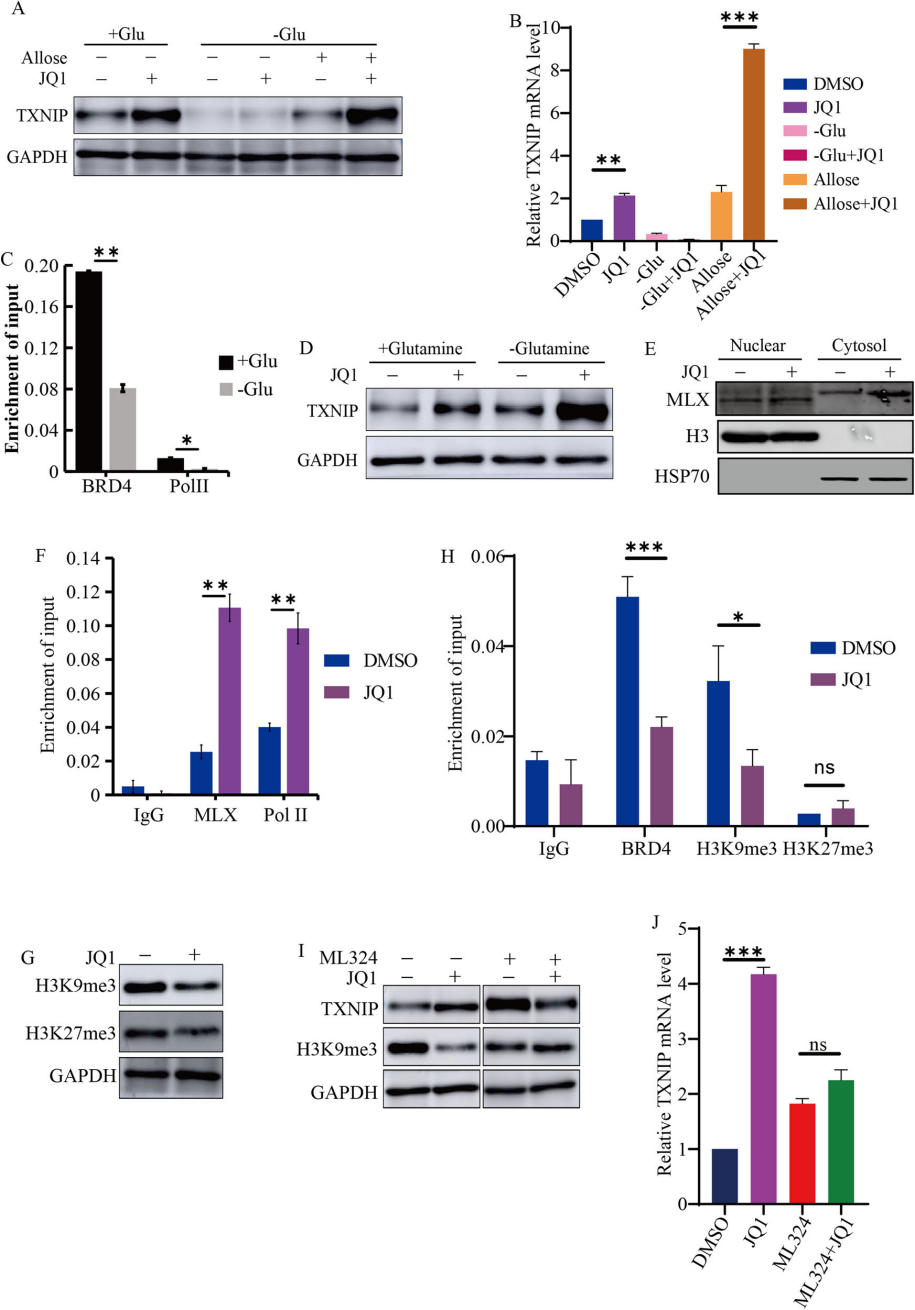
JQ1 increased TXNIP expression in the presence of glucose and inhibited histone H3K9 trimethylation. **A**, **B** The protein (**A**) and mRNA (**B**) levels of TXNIP after JQ1 treatment with or without glucose. **C** The enrichment of BRD4 and RNA Pol II at *TXNIP* promoter in cells cultured with or without glucose. **D** The expression of TXNIP after JQ1 treatment with or without glutamine. **E** The distribution of MLX in cells treated with JQ1. Nuclear and cytosolic fraction were prepared and probed with MLX antibody. **F** The enrichment of MLX and RNA Pol II at *TXNIP* promoter in cells treated with or without JQ1. **G** JQ1 decreased H3K9Me3 and H3K27Me3 levels in HepG2 cells. Whole cell extracts were prepared and probed with indicated antibodies. **H** The levels of BRD4, H3K9Me3, and H3K27Me3 at *TXNIP* promoter in cells treated with or without JQ1. **I** The protein levels of TXNIP and H3K9Me3 in cells treated by JQ1, ML324, and a combination of JQ1 and ML324. **J** The mRNA levels of *TXNIP* in cells treated by JQ1, ML324, and a combination of JQ1 and ML324. ***p value < 0.005, **p value < 0.01, *p value < 0.05 (two-tailed unpaired Student *t* test in **B**, **C**, **F**, **H**, and **J**); ns not significant.

We then queried the mechanisms by which the BET inhibitor activated TXNIP. It is known that TXNIP is a direct and glucose-dependent target of MondoA [23]. Notably, when glucose was deprived from the culture medium, the ability of JQ1 to elevate TXNIP levels was abolished. However, JQ1 augmented TXNIP levels in cell when glucose was replaced by allose, a non-glucose hexose (Fig. 3A, B). Furthermore, glucose deprivation reduced the binding of BRD4 and RNA Pol II to the promoter regions of *TXNIP* (Fig. 3C). In line with previous study [24], glutamine deprivation increased TXNIP protein levels (Fig. 3D), with JQ1 further enhancing its expression in the absence of glutamine (Fig. 3D). These results suggested that the effect of JQ1 on TXNIP expression was contingent upon glucose metabolism rather than glutamine metabolism. The MondoA-MLX heterodimer senses cellular glucose, translocates to the nucleus, and occupies the regulatory regions of *TXNIP* to promote its transcription [25]. Our results indicated that JQ1 treatment facilitated the nuclear translocation of MLX (Fig. 3E). ChIP-PCR analysis revealed an increase in the binding of MLX and RNA Polymerase II to the TXNIP promoter following JQ1 treatment (Fig. 3F). These observations indicated that BRD4 inhibition induced TXNIP transcription by promoting MondoA-MLX activity in the presence of glucose.

We then sought to elucidate the mechanism by which BET inhibition facilitated MondoA-MLX binding to the *TXNIP* promoter. Epigenetic mechanisms, particularly histone modifications, play a crucial role in regulating MLX’s binding to chromatin. Histone acetylation promoted the occupancy of MondoA-MLX on TXNIP promoter [25], however, BRD4 inhibition repressed histone acetylation [26, 27]. We hypothesized that JQ1 would inhibit histone modifications associated with gene repression, such as histone H3K9 and H3K27 trimethylation (Me3), to facilitate MLX binding to chromatin. Western blot analysis confirmed that JQ1 inhibited these histone modifications in HepG2 cells (Fig. 3G). However, ChIP analysis indicated that JQ1 specifically reduced the level of BRD4 and H3K9Me3, but not H3K27Me3, at *TXNIP* promoter (Fig. 3H). The compound ML324, which inhibits the KDM4 demethylase responsible for removing H3K9Me3, was found to reverse the reduction of H3K9Me3 induced by JQ1. Additionally, JQ1 was unable to upregulate TXNIP transcription in the presence of ML324 (Fig. 3I, J). Taken together, JQ1 reduced H3K9Me3 level at the regulatory region of *TXNIP*, promoting MondoA-MLX binding and activating *TXNIP* transcription in the presence of glucose.

### P27 stabilized by TXNIP contributed to JQ1-caused cell cycle arrest

**Fig. 4:**
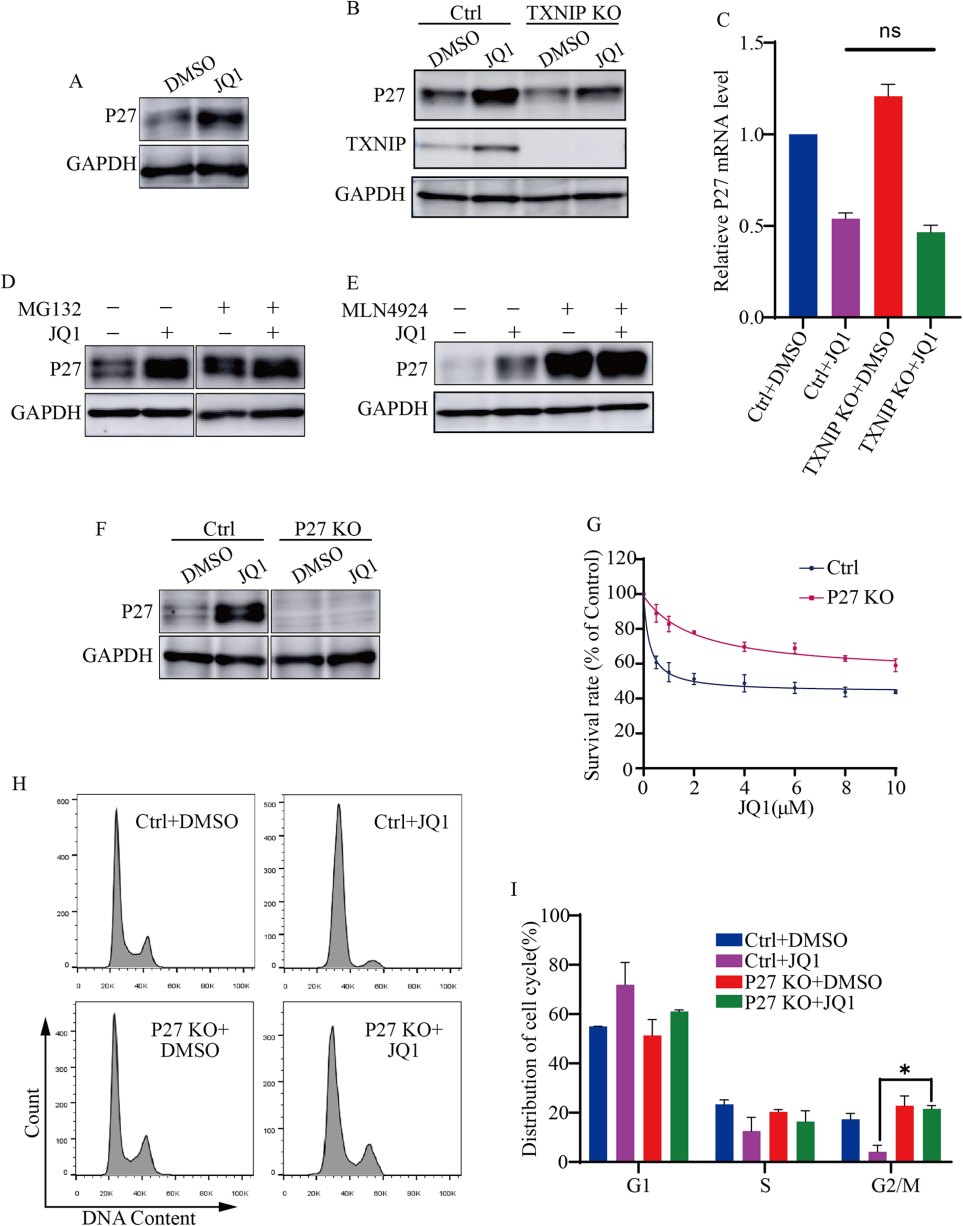
P27 proteins stabilized by TXNIP contributed to JQ1-caused cell cycle arrest. **A** JQ1 led to an increase in P27 expression. Whole cell extracts were prepared and analyzed using a P27 antibody. **B**, **C** The protein (**B**) and mRNA (**C**) level of P27 in Control and TXNIP KO cells treated with DMSO and JQ1. **D** The protein levels of P27 in cells treated by JQ1, MG132, and a combination of JQ1 and MG132. **E** The protein levels of P27 in cells treated by JQ1, MLN4924, and a combination of JQ1 and MLN4924. **F** The expression of P27 in Control and P27 KO cells. P27 in HepG2 cells was knocked out by CRISPR-Cas9. **G** The survival rates of Control and P27 KO cells treated with increasing concentration of JQ1. **H** Flow cytometric analysis of the cell cycle in Control and P27 KO cells treated with JQ1. **I** The distribution of the cell cycle. Quantification of (**H**). *p value < 0.05 (one-way ANOVA test in **C** and **I**); ns not significant.

We continued to explore how TXNIP facilitated JQ1’s effect on the cell cycle. CDK inhibitor activity was increased by JQ1 treatment (Fig. S1E). Notably, JQ1 upregulated one of the CDK inhibitors, P21 (Fig. 1D, E), prompting us to investigate whether other CDK inhibitors like P16 and P27 were also affected. The results revealed that JQ1 did not influence P16 expression (Fig. S4A), but did increase P27 expression (Fig. 4A). Since nuclear P27 inhibits cell cycle progression, we noted that JQ1 also raised nuclear P27 levels (Fig. S4B). To understand whether TXNIP regulated P21 and P27 expression, we treated both control and TXNIP KO cells with JQ1. The results indicated that TXNIP was indispensable for the upregulation of P27 protein following JQ1 treatment, but not for P21 (Figs. 4B and S4A). We then examined the impact of TXNIP on P27 transcription, finding that JQ1 reduced P27 mRNA levels, and TXNIP KO did not change this outcome (Fig. 4C). In the presence of MG132, the proteasome inhibitor, which prevents P27 degradation, JQ1 was less effective at increasing P27 protein levels (Fig. 4D). P27 is ubiquitinated by Cullin-RING ligase (CRLs) [28, 29], which is inhibited by MLN4924 [30, 31]. In line with this, our results showed that MLN4924 stabilized P27. However, JQ1 could not further enhance P27 protein levels in the presence of MLN4924 (Fig. 4E). These data indicated that JQ1-increased TXNIP prevented P27 degradation.

To assess whether P27 was crucial for the anti-tumor effects of JQ1, we knocked out P27 using CRISPR-Cas9 (Fig. 4F). Compared to control cells, P27 KO cells were less sensitive to JQ1 (Fig. 4G). Flow cytometric analysis of the cell cycle revealed that more P27 KO cells entered G2/M phase upon JQ1 treatment (Fig. 4H, I), similar to the TXNIP KO cells. Hence, TXNIP stabilized P27, which contributed to cell cycle arrest in response to JQ1 treatment.

### TXNIP inhibited cMYC activity and protein UFMylation

**Fig. 5:**
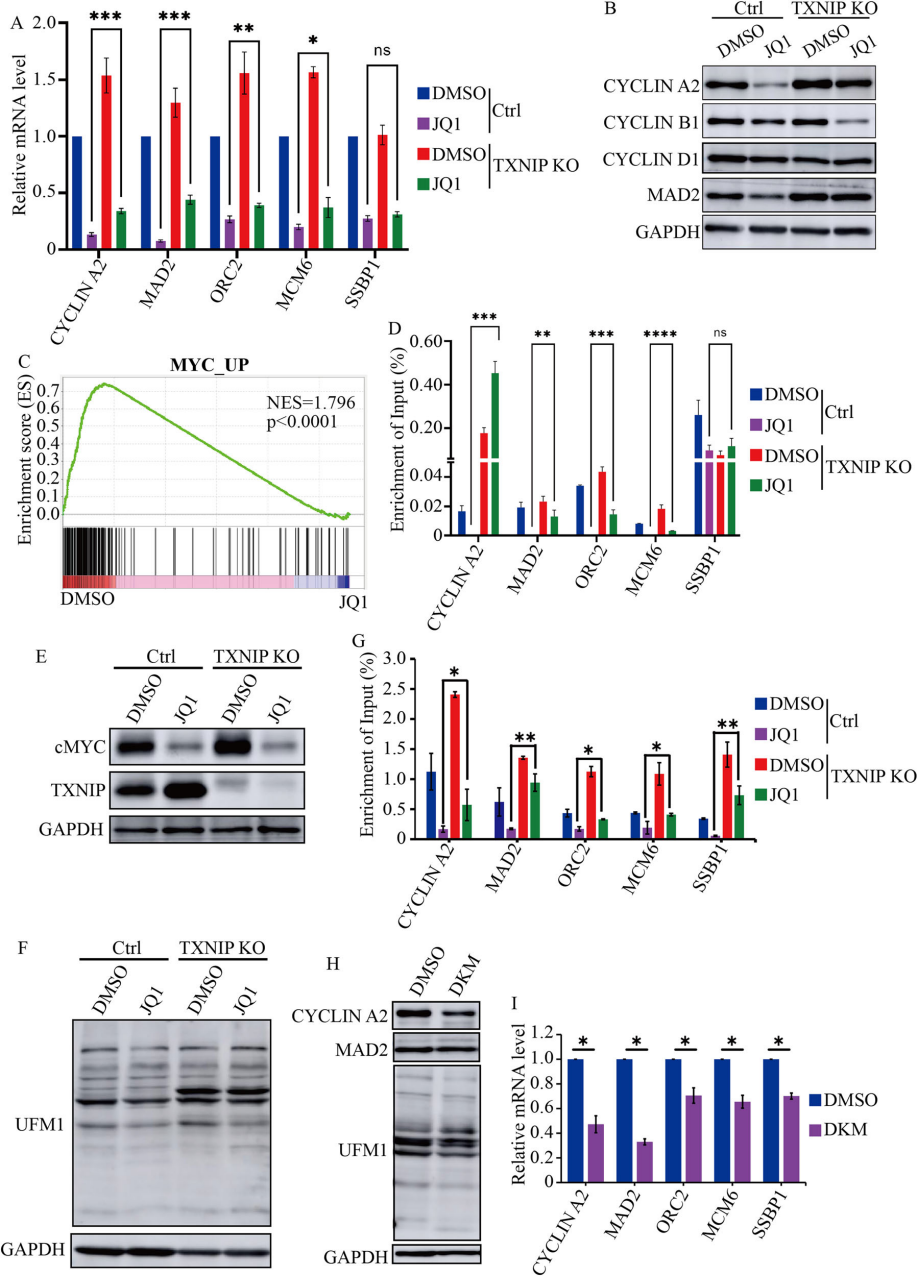
TXNIP inhibited the expression of cMYC targets and protein UFMylation. **A** RT-qPCR analysis of the mRNA levels of *CYCLIN A2*, *MAD2*, *ORC2*, *MCM6*, and *SSBP1* in Control and TXNIP KO cells treated with DMSO and 2 μM JQ1. **B** The protein levels of CYCLIN A2, B1, D1, and MAD2 in Control and TXNIP KO cells treated with DMSO and 2 μM JQ1. **C** GSEA analysis of gene expression in cells with DMSO and 2 μM JQ1 treatment indicated that cMYC target genes were downregulated in JQ1 treated cells. **D** The enrichment of cMYC at the promoters of *CYCLIN A2*, *MAD2*, *ORC2*, *MCM6*, and *SSBP1* in Control and TXNIP KO cells with or without JQ1 treatment. **E** The expression of cMYC in Control and TXNIP KO cells after JQ1 treatment. **F** The level of protein UFMylation in Control and TXNIP KO cells with 2 μM JQ1 treatment. **G** The enrichment of UFMylated proteins at the promoters of *CYCLIN A2*, *MAD2*, *ORC2*, *MCM6*, and *SSBP1* in Control and TXNIP KO cells with or without JQ1 treatment. **H** The protein levels of CYCLIN A2, MAD2, and total protein UFMylation in cells with 30 μM DKM 2-93 treatment. **I** The mRNA levels of *CYCLIN A2*, *MAD2*, *ORC2*, *MCM6*, and *SSBP1* in cells treated with DMSO and 30 μM DKM 2-93. ****p value < 0.001, ***p value < 0.005, **p value < 0.01, *p value < 0.05 (one-way ANOVA test in **A**, **D**, and **G**; two-tailed unpaired Student *t* test in **I**); ns not significant.

TXNIP KO promoted DNA synthesis and G2/M entry in cells treated with JQ1 (Fig. 2C–H). JQ1 repressed the transcription of various cyclins (Fig. 1D), including CYCLIN A2, which is essential for S phase progression and mitotic entry [32–34]. To further characterize TXNIP’s functions in cell cycle regulation, we examined whether TXNIP also regulated CYCLIN A2 expression. TXNIP KO partially restored the mRNA and protein levels of CYCLIN A2 following JQ1 treatment (Fig. 5A, B). To understand how TXNIP regulated the expression of CYCLIN A2, we analyzed our RNA-Seq data with Gene set enrichment analysis (GSEA) and noted that JQ1 repressed several oncogenic gene sets, such as cMYC, MEL18, ERBB2, and MEK pathway (Figs. 5C and S5A–C). Given that CYCLIN A2 was a target of cMYC [35, 36], we proposed that TXNIP might regulate the transcription activity of cMYC. We assessed the expression of other cMYC targets involved in mitosis and DNA replication, such as MAD2, ORC2, MCM6, and SSBP1. Notably, JQ1 repressed their expression, and TXNIP KO reversed the expression of MAD2, ORC2, and MCM6 (Fig. 5A, B). Additionally, JQ1 inhibited cMYC’s binding to the regulatory regions of *CYCLIN A2*, *MAD2*, *ORC2*, and *MCM6*, while TXNIP KO blunted these effects of JQ1 (Fig. 5D). We also examined whether JQ1 and TXNIP influenced E2F1, another key transcription factor in cell cycle regulation, binding to *CYCLIN A2*, *MAD2*, *ORC2*, and *MCM6*. The results showed that JQ1 reduced E2F1 binding to the promoters of *CYCLIN A2* and *MAD2*, but TXNIP KO did not alter JQ1’s effects on these two genes. JQ1 did not change the binding of E2F1 to *ORC2* and *MCM6*, while knocking out TXNIP increased the association of E2F1 with these regions (Fig. S5D). These results implied that JQ1-TXNIP specifically repressed the activities of certain transcription factors, such as cMYC.

To understand how TXNIP regulated cMYC activity, we first examined whether it modulated cMYC expression. JQ1 decreased cMYC expression, which could not be rescued by TXNIP KO (Fig. 5E). As a transcription factor, cMYC binds to cognate sequences on its target genes to modulate their expression. The binding of cMYC to target genes is also influenced by chromatin structure [37, 38]. We hypothesized that JQ1-TXNIP regulated specific histone modifications to modulate cMYC’s chromatin binding. JQ1 inhibited various histone modifications, including H3K9Me3, H3K27Me3, H3K36Me3, H3K27Ac, H3K56Ac, and H4K16Ac, which could not be restored by TXNIP KO (Fig. S5E). We also assessed the level of protein ubiquitination and other ubiquitin-like modifications, including UFMylation and Neddylation, after JQ1 treatment. BET inhibition reduced protein UFMylation but had minimal impact on protein ubiquitination and Neddylation (Figs. 5F and S5F). Importantly, TXNIP KO restored protein UFMylation after JQ1 treatment (Fig. 5F). ChIP-PCR analysis revealed that JQ1 decreased UFMylated proteins at the regulatory regions of *CYCLIN A2*, *MAD2*, *ORC2*, *MCM6,* and *SSBP1*, an effect that could be countered by TXNIP KO (Fig. 5G). Protein UFMylation can be inhibited by DKM 2-93, which, like JQ1, also repressed the expression of CYCLIN A2, MAD2, ORC2, MCM6, and SSBP1 (Fig. 5H, I). Collectively, these findings suggested that JQ1-TXNIP inhibited protein UFMylation and cMYC binding to chromatin, leading to the repression of cell cycle regulators such as CYCLIN A2, MAD2, ORC2, and MCM6.

### JQ1-TXNIP repressed histone H4 UFMylation

**Fig. 6:**
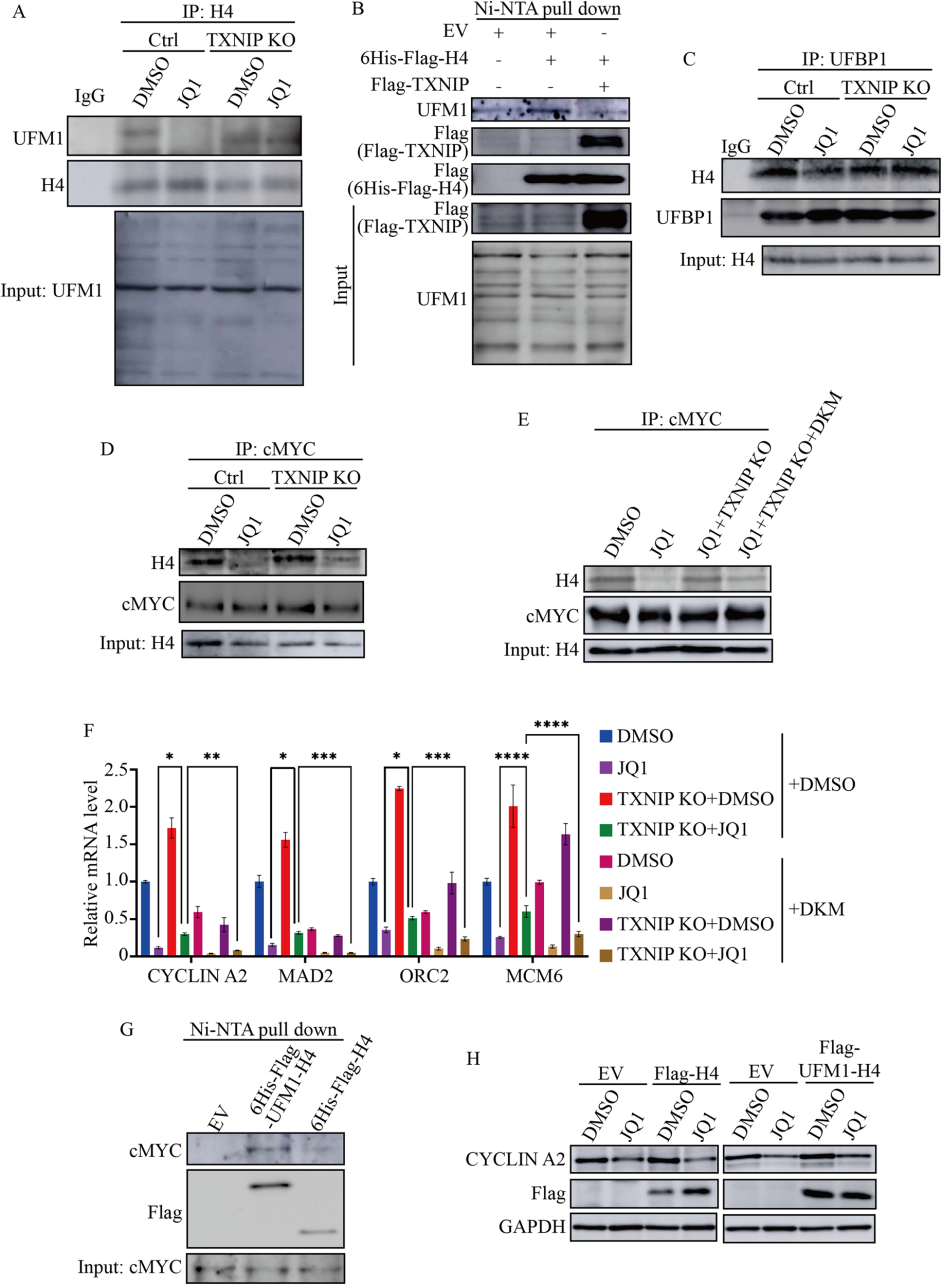
JQ1-TXNIP repressed histone H4 UFMylation. **A** Histone H4 UFMylation in Control and TXNIP KO cells treated with DMSO and 2 μM JQ1. Histone H4 was immunoprecipitated and the precipitated proteins were immunoblotted with UFM1 antibody. **B** Overepression of TXNIP reduced H4 UFMylation. Empty vector (EV), Flag-TXNIP, and 6His-Flag-H4 were transfected into HEK293T cells. 6His-Flag-H4 proteins and their associated proteins were isolated using Ni-NTA beads and analyzed with indicated antibodies. **C** The interaction between histone H4 and UFBP1 in Control and TXNIP KO cells with DMSO and 2 μM JQ1 treatment. The protein complex was immunoprecipitated with UFBP1 antibody and probed with histone H4 antibody. **D** The interaction between histone H4 and cMYC in Control and TXNIP KO cells with and without JQ1 treatment. cMYC was immunoprecipitated and isolated proteins were subjected to Western Blotting analysis with a histone H4 antibody. **E** Protein UFMylation influenced the association between histone H4 and cMYC. cMYC was immunoprecipitated from Control cells treated with DMSO and JQ1 as well as TXNIP KO cells treated by JQ1 alone and JQ1 combined with DKM 2-93. The precipitates were analyzed using a histone H4 antibody. **F** Rescued the expression of cMYC target genes by TXNIP KO was depending on UFMylation. Control and TXNIP KO cells were treated by DMSO, 2 μM JQ1, 30 μM DKM 2-93 and a combination of JQ1 (2 μM) with DKM 2-93 (30 μM). The transcription levels of *CYCLIN A2*, *MAD2*, *ORC2*, and *MCM6* were determined by RT-qPCR. **G** The interaction of cMYC with Flag-UFM1-H4 and Flag-H4. HepG2 cells were transfected with empty vector (EV), 6Hist-Flag-UFM1-H4, and 6Hist-Flag-H4 and their associated proteins were pulled down using Ni-NTA beads and probed for cMYC. **H** UFM1-H4 alleviated the repression of CYCLIN A2 by JQ1. HepG2 cells were transfected with Empty vector (EV), Flag-H4, and Flag-UFM1-H4 and treated with 2 μM JQ1. Total proteins were extracted and CYCLIN A2 expression was analyzed via Western Blotting. ****p value < 0.001, ***p value < 0.005, **p value < 0.01, *p value < 0.05 (one-way ANOVA test in **F**).

We were interested in identifying the chromatin protein whose UFMylation was regulated by JQ1-TXNIP and responsible for the modulation of cMYC binding to its target genes. So far, only a few chromatin proteins have been identified as UFMylated, with histone H4 being one of them [14, 15, 39]. Co-immunoprecipitation (CoIP) assays demonstrated that JQ1 reduced H4 UFMylation, but this reduction was reversed by TXNIP KO (Fig. 6A). Additionally, overexpressing TXNIP in HEK293T cells inhibited H4 UFMylation (Fig. 6B). The pull down assay also showed that TXNIP interacted with histone H4 (Fig. 6B). UFBP1 mediated protein UFMylation [14], and we discovered that JQ1 disrupted the interaction between H4 and UFBP1, which was restored by TXNIP KO (Fig. 6C). Therefore, JQ1-increased TXNIP inhibited the association of H4 with UFBP1, decreasing H4 UFMylation.

We wondered whether TXNIP regulated cMYC’s binding to chromatin by inhibiting H4 UFMylation. JQ1 interrupted the interaction between H4 and cMYC, but TXNIP KO restored their partnership (Fig. 6D, E). Notably, the disruption of the H4-cMYC interaction by JQ1 was not reinstated by TXNIP KO when protein UFMylation was inhibited by DKM 2-93 (Fig. 6E). Consistently, while TXNIP KO restored the transcription of cMYC targets like *CYCLIN A2*, *MAD2*, *ORC2*, and *MCM6* after JQ1 treatment, it failed to alter their transcription in the presence of DKM 2-93 (Fig. 6F). To further clarify the role of H4 UFMylation in regulating cMYC activity, we fused UFM1 with H4 (Flag-UFM1-H4) to create a mimic of UFMylated H4 and investigated its interaction with cMYC. Compared to Flag-H4, the binding of Flag-UFM1-H4 to cMYC was enhanced (Fig. 6G). When Flag-UFM1-H4 was overexpressed in HepG2 cells, it partially mitigated the repression of CYCLIN A2 by JQ1 (Fig. 6H). However, overexpressing Flag-H4 did not changed the protein levels of CYCLIN A2 (Fig. 6H). These findings suggested that JQ1 increased TXNIP, which reduced H4 UFMylation, resulting in the loss of cMYC binding to histone H4 and the inhibition of its transcriptional activity.

### Inhibiting protein UFMylation increased cell sensitivity to ferroptosis

Since cells with low Cyclin A2-CDK2 activity enter a ‘G0-like’ state with hallmarks of senescence [33], we asked whether HepG2 cells treated with the BET inhibitor JQ1 entered a senescent state characterized by irreversible cell cycle exit [40]. Nearly all HepG2 cells were positively stained by beta-Gal after treated with JQ1 for 7 days (Fig. S6A, B). However, several components of the senescence-associated secretory phenotype (SASP), including *IL1ɑ*, *IL6*, *MMP9*, *CSF2,* and *TGFb*, were inhibited by JQ1 (Fig. S6C). Additionally, these treated cells resumed growth once JQ1 was removed (Fig. S6D, E). Coupled with the observation that JQ1 stabilized P27, which marks quiescent cells and drives cancer stem cell features [41, 42], these results indicated that rather than entering senescence, JQ1-treated cells entered a dormant state.

**Fig. 7:**
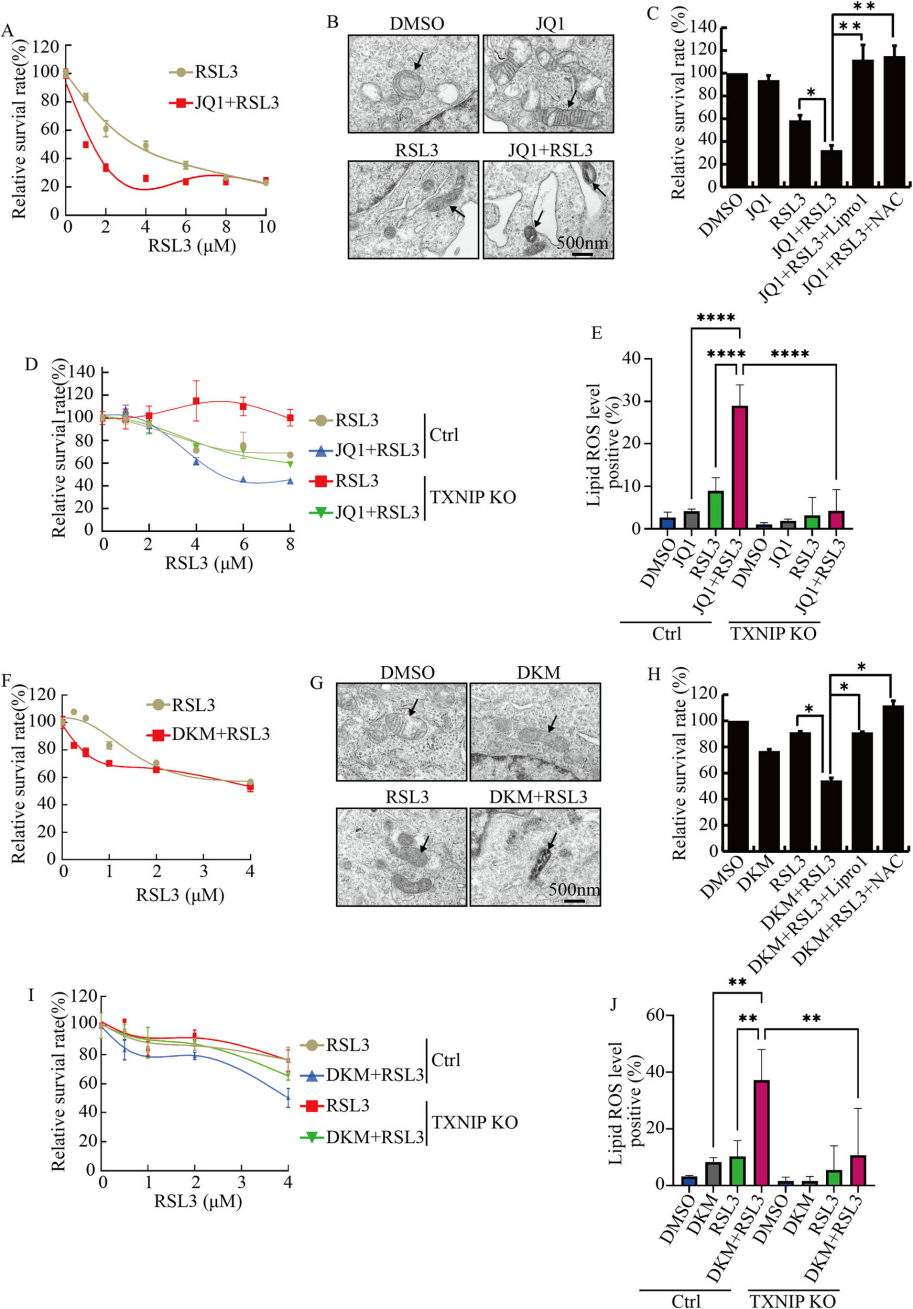
Inhibiting protein UFMylation enhanced ferroptosis sensitivity. **A** The survival rates of cells treated with increasing amount of RSL3 alone or RSL3 combined with 2 μM JQ1. **B** The morphology of mitochondria in cells treated with indicated chemicals was observed by transmission electron microscopy. Scale bars, 500 nm. **C** The survival rates of cells treated with JQ1 (2 μM), RSL3 (6 μM), JQ1 (2 μM) + RSL3 (6 μM), JQ1 (2 μM) + RSL3 (6 μM) with Lipro1 (20 μM), as well as JQ1 (2 μM) + RSL3 (6 μM) with NAC (5 mM). **D** The survival rates of Control and TXNIP KO cells treated with increasing concentration of RSL3 alone or together with 2 μM JQ1. **E** Lipid ROS levels in Control and TXNIP KO cells treated with 2 μM JQ1, 6 μM RSL3, and 2 μM JQ1 + 6 μM RSL3. **F** The survival rates of cells treated with increasing concentration of RSL3 alone or combined with 30 μM DKM 2-93. **G** The mitochondrial ultrastructure in cells treated with indicated chemicals. **H** The survival rates of cells treated with DKM 2-93 (30 μM), RSL3 (2 μM), DKM 2-93 (30 μM) + RSL3 (2 μM), DKM 2-93 (30 μM) + RSL3 (2 μM) with Lipro1 (20 μM), as well as DKM 2-93 (30 μM) + RSL3 (2 μM) with NAC (5 mM). **I** The survival rates of Control and TXNIP KO cells treated with increasing amount of RSL3 alone or in combination with 30 μM DKM 2-93. **J** Lipid ROS levels in Control and TXNIP KO cells treated with 30 μM DKM 2-93, 2 μM RSL3, and 30 μM DKM 2-93 + 2 μM RSL3. ****p value < 0.001, **p value < 0.01, *p value < 0.05 (one-way ANOVA test in **C**, **E**, **H**, and **J**).

We were interested in finding ways to eliminate these quiescent cells, which could cause drug resistance and tumor recurrence [43, 44]. It was reported that dormant cells were more susceptible to ferroptosis inducers [17, 18]. Additionally, the transcription of genes such as SAT1, HMOX1, and ACSL1, which sensitize cells to ferroptosis, were all increased by JQ1 [45–47] (Fig. S6F, G). We thus studied whether JQ1 increased the sensitivity of HepG2 cell to ferroptosis. Cells were treated with increasing concentrations of RSL3, a type II ferroptosis inducer targeting GPX4, alongside DMSO and JQ1. The results demonstrated that JQ1 did increase HepG2’s sensitivity to RSL3 (Fig. 7A). The characteristics of ferroptosis, such as shrunken mitochondria with dense structures, and the loss of mitochondrial cristae, were observed in cells treated with both JQ1 and RSL3 (JQ1 + RSL3) (Fig. 7B). The inhibitory function of JQ1 + RSL3 were diminished by the ferroptosis inhibitor Lipro 1 and N-acetyl cysteine (NAC) (Fig. 7C). TXNIP KO cells were more resistant to JQ1 + RSL3 treatment compared to control cells (Fig. 7D). Reactive oxygen species (ROS) and lipid peroxidation drive ferroptosis. RSL3 increased the production of lipid ROS and cellular ROS, with JQ1 amplifying these effects (Figs. 7E and S7A, B). In contrast, knocking out of TXNIP limited the production of lipid ROS and cellular ROS triggered by RSL3 + JQ1 (Figs. 7E and S7A, B). Similar results were observed when cells were treated with the type I ferroptosis inducers Erastin, which targets the xCT transporter (Fig. S7C–F). These findings indicated that increased TXNIP upon JQ1 treatment disrupted redox homeostasis and enhanced ferroptosis sensitivity.

Our studies also demonstrated that TXNIP suppressed H4 UFMylation, leading to cell cycle arrest. To determine if protein UFMylation regulated ferroptosis, we treated cells with RSL3 and Erastin in the presence of DKM 2-93 and found that DKM 2-93 also heightened sensitivity to these ferroptosis inducers in HepG2 cells (Figs. 7F and S7G). DKM 2-93 and RSL3 co-treatment also changed mitochondrial ultrastructure, resulting in smaller mitochondria (Fig. 7G). Ferroptosis inhibitor Lipro1 and NAC mitigated the effects of DKM 2-93 and RSL3 co-treatment on cell viability (Fig. 7H). Additionally, TXNIP KO attenuated the functions of DKM 2-93 in promoting ferroptosis (Figs. 7I and S7H). Knocking out of TXNIP also diminished the production of lipid ROS triggered by RSL3 + DKM 2-93 (Fig. 7J). These results indicated that inhibiting BRD4 and protein UFMylation sensitized HepG2 cells to ferroptosis.

To figure out whether JQ1 increased ferroptosis sensitivity in other cancer cells, we investigated its effects on HCT116 cells. JQ1 elevated TXNIP expression (Fig. S1F) while reduced the protein level of cMYC, CYCLIN A2, CYCLIN B1, and Ki67 (Fig. S8A). It also inhibited protein UFMylation in HCT116 cells (Fig. S8A). Importantly, JQ1 treatment increased the sensitivity of HCT116 to RSL3 (Fig. S8B). The combination of JQ1 and RSL3 significantly raised the level of lipid ROS (Fig. S8C). We also treated cells with DKM 2-93 and RSL3, finding that this combination induced more lipid ROS than either agent alone (Fig. S8D). Collectively, these findings suggested that inhibiting protein UFMylation by JQ1 and DKM 2-93 increased cancer cells’ sensitivity to ferroptosis inducers.

### BET inhibitor increased the anti-tumor effects of ferroptosis inducer in vivo

**Fig. 8:**
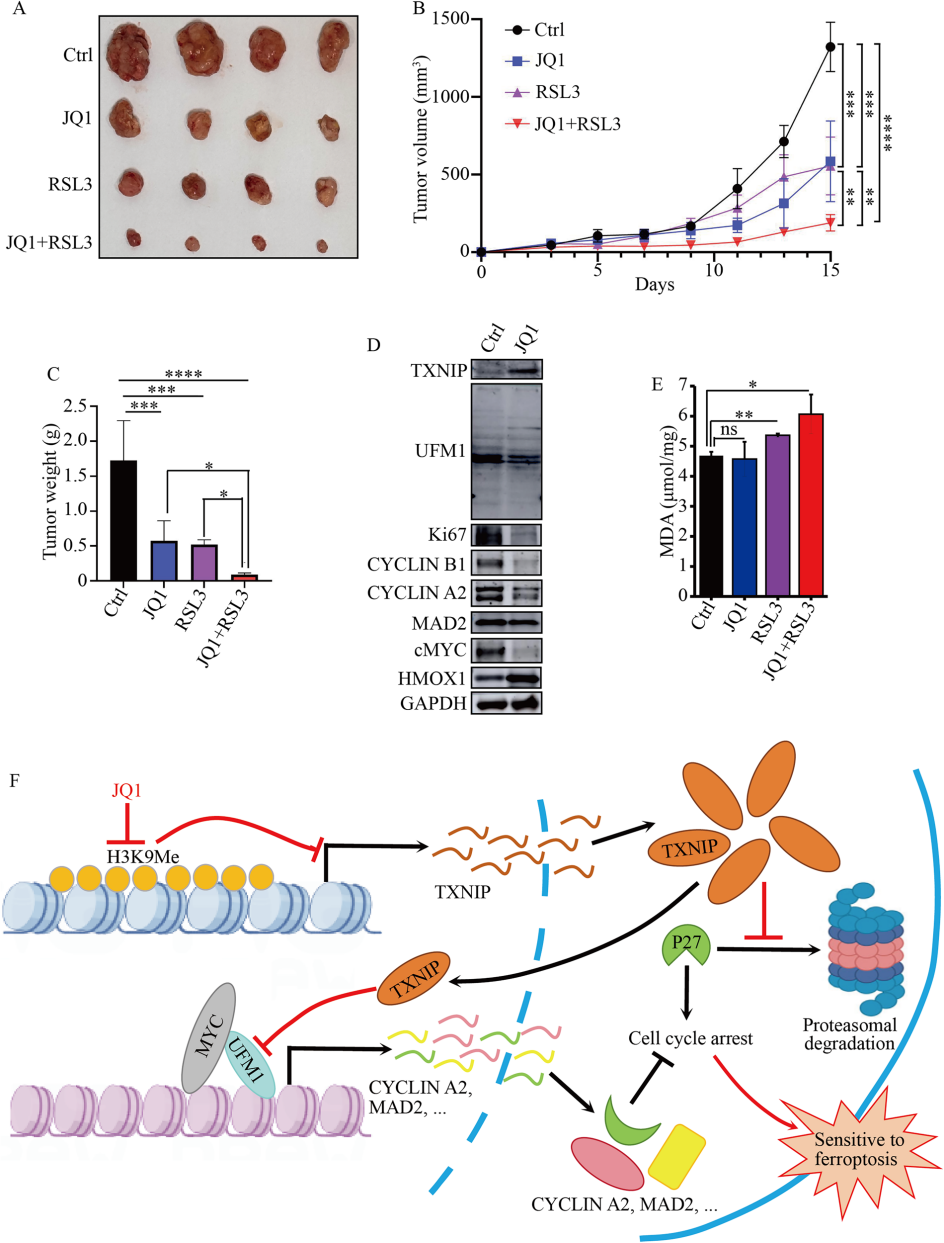
Inhibiting BRD4 increased tumor sensitivity to ferroptosis inducer in vivo*.* **A** Representative images of the HCT116 xenografts treated with control vehicle, JQ1, RSL3, or JQ1 + RSL3. **B** The growth curve of HCT116 xenografts treated with indicated chemicals in mice. **C** The tumor weight of HCT116 xenografts after administration of control, JQ1, RSL3, or JQ1 + RSL3. **D** The expression levels of indicated proteins in HCT116 xenografts treated with control and JQ1. Total proteins were extracted from tumor tissues treated with control and JQ1. **E** The concentration of malondialdehyde (MDA) in tumor tissues treated with indicated compounds.****p value < 0.001, ***p value < 0.005, **p value < 0.01, *p value < 0.05 (one-way ANOVA test in **B**, **C**, and **E**). **F** BRD4 inhibition increased TXNIP expression and caused cell cycle arrest, sensitizing cancer cells to ferroptosis. BET inhibitors, such as JQ1, inhibited H3K9Me3 at the *TXNIP* promoter and increased TXNIP expression. TXNIP stabilized CDK inhibitor P27 by inhibiting its proteasomal degradation. Additionally, TXNIP disrupted the interaction between histone H4 and UFBP1 and reduced H4 UFMylation, inhibiting cMYC binding to its target genes and silencing the transcription of cell cycle regulators, such as CYCLIN A2, MAD2, ORC2, and MCM6. Consequently, cells treated with BET inhibitors entered a dormant state and became sensitive to ferroptosis.

To investigate whether BRD4 inhibition promoted ferroptosis in vivo, we established tumor xenografts with HCT116 cells in nude mice and treated these mice with vehicle, JQ1, RSL3, and a combination of JQ1 and RSL3 (JQ1 + RSL3), respectively. Tumor growth curve and weight were measured and results showed that the combination of JQ1 and RSL3 was more effective in inhibiting tumor growth and reducing tumor weight than either JQ1 or RSL3 alone (Fig. 8A–C). Consistent with the findings in cultured cancer cells, JQ1 increased TXNIP expression while inhibited protein UFMylation in the tumor xenografts (Fig. 8D). Moreover, it reduced the expression of Ki67 and other cell cycle regulators such as CYCLIN B1, A2, MAD2, and cMYC (Fig. 8D), suggesting that BET inhibitor could cause cell cycle arrest in vivo. To determine if the inhibition of tumor growth was due to the induction of ferroptosis, we measured the concentration of malondialdehyde (MDA), a byproduct of lipid peroxidation. Treatments with RSL3 and JQ1 + RSL3 indeed raised MDA levels in tumor tissues (Fig. 8E). Together, these results suggested that inhibiting BRD4 with JQ1 disturbed cell cycle progression, enhancing the anti-tumor effects of RSL3 in tumor xenografts in mice.

## Discussion

Our studies have revealed that BET inhibitors, such as JQ1, inhibited H3K9Me3 at *TXNIP* promoter, leading to increased TXNIP expression and subsequent cell cycle arrest. Mechanistically, elevated TXNIP stabilized CDK inhibitor P27 by inhibiting its proteasomal degradation. Additionally, TXNIP disrupted the interaction between histone H4 and UFBP1, decreasing H4 UFMylation, which in turn prevented cMYC from binding to its target genes and silenced the transcription of cell cycle regulators. The resulting increase of P27 and repression of cell cycle regulators by TXNIP ultimately causes cells to enter a dormant state, making them more susceptible to ferroptosis inducers (Fig. 8F).

While BRD4 is typically recognized as a transcriptional activator, its inhibition led to the activation of TXNIP, even shortly after the application of the BRD4 inhibitor, suggesting that BRD4 may specifically repress the expression of certain genes. It was possible that BRD4 recruited chromatin regulators to facilitate H3K9 trimethylation at the TXNIP promoter. This aligned with previous studies indicating that BRD4 configured chromatin structure to suppress gene expression [2, 11–13].

Recently, BRD4 has gained attention as a potential therapeutic target in cancer treatment. Inhibiting BRD4 with BET inhibitors repressed cMYC expression, leading to tumor suppression [5]. Our findings indicated that TXNIP suppressed cMYC transcriptional activity. Thereby, in addition to directly reduced cMYC expression, BET inhibitors could also disrupt cMYC activity by increasing TXNIP levels. Since cMYC activity determined cellular responses to BET inhibitors [5], TXNIP might represent an additional molecular feature that governed cell sensitivity to these agents. Clarifying the functions of TXNIP may be crucial for elucidating the anti-cancer mechanisms of BET inhibitors.

TXNIP was involved in controlling glucose and lipid metabolism, cell cycle progression, inflammasome activation, and autophagy [48]. Previous research also indicated that TXNIP interfered with the binding of cMYC to chromatin, but the underlying mechanisms were not well understood [36]. Our findings revealed that TXNIP inhibited H4 UFMylation and disrupted the interaction between H4 and cMYC, suggesting that TXNIP modulated cMYC’s transcriptional activity by regulating histone UFMylation. As a transcription factor, cMYC recognizes and binds to specific DNA sequence to regulate gene transcription, and its activity is also influenced by chromatin structure [38]. The mechanism by which H4 UFMylation enhances cMYC binding to H4 remains to be explored further.

Protein UFMylation is a posttranslational modification similar to ubiquitination, but our understanding of its functions and regulatory mechanisms is still limited [14, 15]. BET inhibitors induced TXNIP, which regulated histone H4 UFMylation by modulating H4-UFBP1 interaction, suggesting that BRD4 and TXNIP were regulators of protein UFMylation. However, the exact mechanisms through which TXNIP disrupts the association of H4 and UFBP1 are not yet clear.

The biomarkers that determine sensitivity to ferroptosis inducers are still not well defined. In line with earlier studies [49–51], we found that BRD4 might serve as a predictor of ferroptosis sensitivity. Inhibiting BRD4 increased TXNIP levels and suppressed protein UFMylation, resulting in cancer cell cycle arrest and increased vulnerability to ferroptosis. This is consistent with previous studies showing that dormant cancer cells were sensitive to ferroptosis [17, 18]. Therefore, while dormant cells induced by BET inhibitors may contribute to drug resistance [52], they also presented a therapeutic vulnerability to ferroptosis inducers, which could be leveraged in combination therapies. Our research indicates that BET inhibition led to the induction of TXNIP, which caused cell cycle arrest, offering new insights into the anti-tumor mechanisms of BET inhibitors. Accordingly, targeting these arrested cells with ferroptosis inducers may provide strategies to improve the clinical outcomes of BRD4-targeted therapies.

## Materials and Methods

### Cell culture, reagents, and cell transfection

Human HepG2, MHCC-97H, SMMC-7721, A375, Capan-2, and HCT116 cells, and mouse Hepa 1–6 cells were purchased from Cell Bank, Chinese Academy of Sciences. All cells were cultured in DMEM medium (Gibco) supplemented with 10% fetal bovine serum (Gibco), 100 U/mL penicillin, and 100 μg/mL streptomycin in a humidified atmosphere containing 5% CO_2_ at 37 °C.

BET inhibitor JQ1 (A1910) was purchased from APExBIO. ML324 (HY-12725), MG132 (HY-13259), MLN4924 (HY-70062), DKM 2-93 (HY-101836), RSL3 (HY-100218A), and Liproxstatin-1 (LIPRO1, HY-12726) were from MCE. Erastin (E126853) and Allose (A111907) was purchased from Alading. NAC (50303ES05) was from Yeasen. JQ1 was dissolved in DMSO to make a 2 mM stock solution. Cells were treated with 2 μM JQ1 for 48 h unless otherwise indicated. RSL3 and ERASTIN were dissolved in DMSO to make a 10 mM stock solution and used to treated cells with indicated concentration. Where applicable, cells were treated with 20 μM ML324 for 24 h, 5 μM MLN4924 for 24 h, 30 μM of DKM 2-93 for 48 h, 20 μM LIPRO1 for 24 h, and 5 mM NAC for 24 h. Ten μM of MG132 were used to treat cells for 6 h before harvesting cells.

To knock out TXNIP and P27 in HepG2 cells, Lipo2000 (Thermo Fisher, 11668019) was used to transfect the TXNIP and P27 sgRNA expression plasmids into cells following the instructions. Forty-eight hours after the transfection, cells were selected in culture media with 2 μM Puromycine (CSN Pharma, CSN11799). The knock-out efficiency was checked by western blotting.

### Plasmids and antibodies

The human TXNIP and human P27 sgRNA expression plasmids were constructed according to Ran et al. [53]. Briefly, the oligos of sgRNA were annealed and ligated into the pSpCas9(BB) vector (Addgene PX549) using BbsI (NEB). The target sequences of TXNIP were: KO-1: CACCGAATATGGGTGTGTAGACTAC, KO-2: CACCGTCTGCTCGAATTGACAGAAA. The target sequences of P27 were CACCGTCAAACGTGCGAGTGTCTAA. The target sequences of control were CACCGATCCTGGCTACCAGCTTCAT. All of the plasmids were validated by sequencing.

TXNIP antibody (14715), MYC (13987), and P21 (2947T) were purchased from Cell Signaling Technology. Antibodies against H3 (17168-1-AP), ubiquitin (10201-2-AP), MAD2 (10337-1-AP), and IgG (30000-0-AP) were purchased from Proteintech. The following antibodies were from Abclonal Technology: P27 (A16332), GLUT1 (A11727), KI67 (A20018), UFM1 (A15843), Nedd8 (A22568), BRD4 (A12677), H4 (A17024), RNA Pol II (A11181), Cyclin A2 (A19036), H3K9me3 (A22295), H3K27me3 (A2771), H3K36me3 (A20379), Cyclin B1 (A2056), Cyclin D1 (A1301) and GAPDH(AC002). MLX antibody was from ABways (AY3005).

### MTT assay

About 3 × 10^3^/well cells were plated in 96-well plates. Then the cells were treated with indicated chemicals for 48 h. After treatment, MTT was added into the medium and incubated at 37 °C for another 4 h. Finally, formed formazan was dissolved in 100 μL DMSO and the absorbance of the solution was measured at 570 nm.

### Quantitative real-time polymerase chain reaction (RT-qPCR)

Total cellular RNA was extracted using the RNA isolator (Vazyme, Nanjing, China) following the manufacturer’s instruction. HiScript II 1st Strand cDNA Synthesis Kit (Vazyme, Nanjing, China) was used to synthesize the first strand cDNA with one microgram of total RNA. The RT-qPCR was performed in the ABI PRISM Sequence Detector 7500 (PerkinElmer) with SYBR Green PCR Master Mix (Vazyme, Nanjing, China) using two hundred nanograms of cDNA. The sequences of the RT-qPCR primers were listed in Supplemental Table 1.

### Western blotting

Cells were treated with chemicals at the indicated concentration for 24 h. After treatment, cells were harvested and lysised in 2% SDS supplemented with protease inhibitors. Protein concentration was measured with BCA assay kit following the instruction. Western blotting was performed as previously described [26]. Briefly, about 20 μg of cell extracts were separated in SDS-PAGE gel. After electrophoresis, proteins were transferred onto nitrocellulose membranes, which were then blocked with 3% skim milk in PBST at room temperature and incubated with indicated primary antibodies overnight at 4 °C. After being washed for three times with PBST, the membranes were incubated with HRP-conjugated secondary antibody for 1 h at room temperature. Finally, the blot was developed with enhanced ECL Chemiluminescent Substrate (Yeasen) and the images were acquired by Amersham Imager 600 (GE Healthcare, USA).

### Immunostaining in cell culture

Cultured cells were washed with PBS for three times, fixed with 4% paraformaldehyde for 30 min, permeabilized with 0.25% Triton ×-100 for 15 min, and blocked with 10% fetal bovine serum for 2 h at room temperature. Samples were then incubated with indicated primary antibodies overnight at 4 °C. After being washed with PBS for three times, cells were incubated with Goat anti-Rabbit/anti-Mouse IgG (H + L) Cross-Adsorbed Secondary Antibodies (Thermo, Waltham, USA) for 2 h at room temperature. Nuclei were counter-stained with DAPI (Yeasen). Images were taken with the confocal laser scanning microscope (Fluoview FV1000, Olympus).

### EdU staining

Cells were cultured in medium containing 50 μM EdU for 2 h to label proliferating cells. Then cells were harvested and fixed with 4% paraformaldehyde for 15 min. After that, the cells were permeabilized with 0.5% TritonX 100 for 20 min and incubated with Click-iT reaction for 30 min in dark. Finally, the nuclei were counter-stained with DAPI and images were taken by the confocal laser scanning microscope (Fluoview FV1000, Olympus).

### Beta-gal staining

Beta-gal staining was performed following the instructions of the Beta-gal staining kit (Yeasen). Cells were treated with indicated drugs and then incubated with the fixed solution at room temperature for 15 min. After washed with PBS for three times, cells were stained with the staining solution at 37 °C overnight. The next day, staining solution was removed and cells were washed with PBS. Images were taken by the Nikon Eclipse Ti-S microscope.

### Cell apoptosis analysis

Cells were treated with the indicated concentrations of chemicals for 48 h. Subsequently, they were stained with Annexin-V conjugated with FITC and Propidium iodide (PI) (Vazyme, A211-0) for 10 min at room temperature to detect apoptosis following the product’s instructions. The samples were analyzed with a FACS Calibur cytometer (BD, Biosciences) and data were processed with FlowJo.

### Lysotracker staining

Staining with Lysotracker RED was optimized for acidic protein content estimation. The cells were stained with Lysotracker RED (1:10,000, Beyotime Biotechnology C1046) for 30 min at 37 °C. Finally, the cells were rinsed, suspended in PBS and analyzed with a FACS Calibur cytometer (BD, Biosciences). Data were processed with FlowJo.

### ROS detection

The dye DCFH-DA was prepared at a ratio of 1:1000 in serum-free DMEM. Control and TXNIP KO Cells were treated with indicated drugs, and harvested using trypsin. These cells were re-suspended in 100 μl prepared DCFH-DA solution and then incubated at 37 °C for 20 min in the dark. The fluorescence intensity of DCFH-DA was determined by FACS Calibur cytometer (BD, Biosciences). Data were processed with FlowJo.

### Lipid ROS assay

HepG2 and HCT116 cells were treated with indicated chemicals for 24 h. Treated cells were harvested, resuspended in 500 μl of PBS containing 5 μM C11-BODIPY 581/591 (Beyotime Biotechnology) and stained for 30 min at 37 °C. Lipid peroxidation was measured by FACS Calibur cytometer (BD, Biosciences) with a 488-nm laser on the FL1 detector. At least 10,000 single cells were analyzed in each group. Lipid peroxidation results were processed by FlowJo and the untreated wild-type cell group was selected as lipid ROS 2%.

### MDA assay

The MDA concentration in tumor tissues were determined using Lipid Peroxidation MDA Assay Kit (Beyotime, S0131S) according to the manufacturer’s instruction. Briefly, tumor tissues were ground in homogenizer. After centrifuged at 12,000 × *g* for 10 min, the supernatant was mixed with MDA assay working solution. The optical density was measured at 532 nm. The concentration of MDA was normalized with protein concentration.

### Electron microscopy

Treated HepG2 cells were harvested and fixed in ice-cold Electron Microscope Fixing Agent (SPI Supplies, 111–30-8). The cells were then fixed with 1% OsO4 in 0. 1 M PBS (pH 7.4) for 2 h at room temperature. Fixed samples were dehydrated in graded series of ethanol (50%, 70%, 95%, 100%) and acetone for 15 min. After embedded in EMBed 812, samples were moved into 60 °C oven to polymerize for more than 48 h. Embedded blocks were ultrathin sectioned (RMC, PT-PC) and stained with 2% uranium acetate and 2% Lead citrate sequentially. Finally, they were observed under a transmission electron microscope (HITACHI, HT7800) and images were collected for analysis.

### Cell cycle analysis

Cell cycle was analyzed following the manufacture’s protocol (KeyGen Biotech). Briefly, cells were treated with indicated chemicals, fixed with 70% cold ethanol for 2 h at 4 °C and then stained with PI working solution for 1 h in dark. The fluorescence intensity of PI was determined by FACS Calibur cytometer (BD, Biosciences). Data were processed with FlowJo.

### Nuclear and cytoplasm fraction

Cells were harvested and resuspended in nuclei isolation buffer (10 mM Tris-Cl (pH 7.5), 10 mM NaCl, 3 mM MgCl2, 0.5% NP40, and 10% glycerol) with freshly added protease inhibitors. After incubated on ice for 30 min, the cells were passed through a 26 gauge needle to break the cell membrane. Cells were then centrifuged at 1000 × *g* for 5 min at 4 °C. The supernatant was kept as cytoplasm fraction. The pellet were washed with nuclei isolation buffer for 3 times. Nuclear proteins were extracted from purified nuclei by 2% SDS.

### Protein co-immunoprecipitation (CoIP) assay

To detect the endogenous protein interaction, CoIP assay was carried out as previously described [26]. Briefly, total cells were prepared with NP40 lysis buffer (Beyotime Biotechnology). Whole cell extracts were cleared by centrifuged at 13000 rpm for 15 min at 4 °C, and then incubated with 4 μg primary antibody or control IgG overnight at 4 °C. The next day, Magnetic Protein A/G beads (MCE, HY-K0202) were added to pull down the antibody-protein complex. After washed with PBST solution for five times, the beads were boiled with 1 × SDS-PAGE Loading Buffer and the samples were analyzed by immunoblotting. Protease inhibitors were applied during all these processes.

### Chromatin immunoprecipitation

Control and TXNIP KO HepG2 cells were treated with 2 μM JQ1 for 3 h, then were fixed with 1% formaldehyde for 10 min. Fixed cells were lysised in lysis buffer (1% SDS, 50 mM Tris-HCl(pH8.0), 150 mM NaCl) and chromatin was broken down by sonication in Covaris M220. Supernatant was diluted with dilution buffer (1% TritonX100, 2 mM EDTA, 20 mM Tris-HCl(pH8.0), 150 mM NaCl) and incubated with IgG, UFM1, MYC, MLX, BRD4, and RNA Pol II antibody at 4 °C overnight. The next day, the antibodies were precipitated with Protein A/G Magnetic Beads. Then the beads were washed sequentially with Washing buffer 1 (0.1% SDS, 1% TritonX 100, 2 mM EDTA, 150 mM NaCl, 20 mM Tris-HCl(pH8.0)), Washing buffer 2 (0.1% SDS, 1% TritonX 100, 2 mM EDTA, 500 mM NaCl, 20 mM Tris-HCl(pH8.0)), Washing buffer 3 (0.25 mM LiCl, 1% NP40, 1 mM EDTA, 10 mM Tris-HCl(pH8.0)), Washing buffer 4 (10 mM Tris-HCl(pH8.0), 2 mM EDTA). Finally, bound DNA was eluted by elution buffer (0.1 M NaHCO3, 1% SDS), purified by phenol, and applied for RT-qPCR analysis. Primers were listed in Supplemental Table 1.

### mRNA sequencing by Illumina HiSeq

Total RNA of each sample was extracted using TRIzol Reagent/RNeasy Mini Kit (Qiagen), quantified and qualified by Agilent 2100 Bioanalyzer (Agilent Technologies, USA). One μg of total RNA was used for library preparation. Next generation sequencing library preparations were constructed according to the manufacturer’s protocol. First strand cDNA was synthesized using ProtoScript II Reverse Transcriptase and the second-strand cDNA was synthesized using Second Strand Synthesis Enzyme Mix. Then the libraries were sequenced in Illumina HiSeq instrument according to manufacturer’s instructions (Illumina, USA). Data quality control was processed by Cutadapt. Clean data were aligned to reference genome via software Hisat2. DESeq2 Bioconductor package was used to identify differentially expressed genes. GOSeq(v1.34.1) was used to identify Gene Ontology (GO) terms that annotate a list of enriched genes with a significant *p* value less than 0.05. Geneset enrichment in the RNA-Seq data were analyzed by GSEA package. The RNA-seq data has been deposited into the GEO database and the GEO accession number is GSE298083.

### Animal studies

The animal studies were approved by Institutional Animal Care and Use Committee of China Pharmaceutical University. Female BALB/c nude mice (6–8 weeks old) weighing 18–22 × *g* were purchased from Changzhou Cavens Laboratory. About 6 × 10^6^ HCT116 cells were transplanted into BALB/c nude mice subcutaneously. When the tumors were palatable, the mice with similar body weight were separated into the following four groups randomly: control group, JQ1 group, RSL3 group, and JQ1 + RSL3 group. Each group had 4 animals. JQ1 (50 mg/kg) was applied to mice by intraperitoneal injection and RSL3 (100 mg/kg) was administered by intratumor injection every two days. Tumor volume was measured every two days using caliper and calculated by using the formula V = (W^2^ × L)/2 (W: tumor width, L: tumor length). After 14 days, mice were sacrificed and tumor tissues were taken off for weighing. Total proteins from tumor tissues were extracted by RIPA buffer and used for Western blotting analysis. Data was processed with Graphpad Prism. We were not blinded to the group allocation during the experiment and/or when assessing the outcome.

### Statistical analysis

Data were presented as means ± SD of at least three independent experiments. Comparisons were carried out with a two-tailed unpaired Student’s *t* test or one-way ANOVA test using GraphPad Prism as appropriate.

## Supplementary information

supplemental figure 1

supplemental figure 2

supplemental figure 3

supplemental figure 4

supplemental figure 5

supplemental figure 6

supplemental figure 7

supplemental figure 8

supplemental figure legends

supplemental table 1

supplemental original WB figures

## Data Availability

All data generated during this study are included in this article and its supplementary information files.

## References

[1] Zheng B, Gold S, Iwanaszko M, Howard BC, Wang L, Shilatifard A. Distinct layers of BRD4-PTEFb reveal bromodomain-independent function in transcriptional regulation. Mol Cell. 2023;83:20. 10.1016/j.molcel.2023.06.032 PMC10527981 37442129

[2] Liang Y, Tian J, Wu T. BRD4 in physiology and pathology: “BET” on its partners. Bioessays. 2021;43:e2100180. 34697817 10.1002/bies.202100180

[3] Devaiah BN, Singh AK, Mu J, Chen Q, Meerzaman D, Singer DS. Phosphorylation by JNK switches BRD4 functions. Mol Cell. 2024;84:4282–96.e7. 39454579 10.1016/j.molcel.2024.09.030 PMC11585421

[4] Padmanabhan A, de Soysa TY, Pelonero A, Sapp V, Shah PP, Wang QH, et al. A genome-wide CRISPR screen identifies BRD4 as a regulator of cardiomyocyte differentiation. Nat Cardiovasc Res. 2024;3:34. 10.1038/s44161-024-00431-1 PMC11361716 39196112

[5] Sabò A, Amati B. BRD4 and MYC-clarifying regulatory specificity. Science. 2018;360:713–4. 29773735 10.1126/science.aat6664

[6] Muhar M, Ebert A, Neumann T, Umkehrer C, Jude J, Wieshofer C, et al. SLAM-seq defines direct gene-regulatory functions of the BRD4-MYC axis. Science. 2018;360:800–5. 29622725 10.1126/science.aao2793 PMC6409205

[7] Wu T, Pinto HB, Kamikawa YF, Donohoe ME. The BET family member BRD4 interacts with OCT4 and regulates pluripotency gene expression. Stem Cell Rep. 2015;4:390–403. 10.1016/j.stemcr.2015.01.012 PMC4375790 25684227

[8] Chandrashekar DS, Afaq F, Karthikeyan SK, Athar M, Shrestha S, Singh R, et al. Bromodomain inhibitor treatment leads to overexpression of multiple kinases in cancer cells. Neoplasia. 2024;57:10. 10.1016/j.neo.2024.101046 PMC11408867 39241280

[9] Latif AL, Newcombe A, Li S, Gilroy K, Robertson NA, Lei X, et al. BRD4-mediated repression of p53 is a target for combination therapy in AML. Nat Commun. 2021;12:16. 33431824 10.1038/s41467-020-20378-8 PMC7801601

[10] Yan L, Tan SY, Wang HH, Yuan H, Liu XH, Chen Y, et al. Znf687 recruits Brd4-Smrt complex to regulate gfi1aa during neutrophil development. Leukemia. 2024;38:851–64. 38326409 10.1038/s41375-024-02165-2

[11] Sakamaki JI, Wilkinson S, Hahn M, Tasdemir N, O’Prey J, Clark W, et al. Bromodomain protein BRD4 is a transcriptional repressor of autophagy and lysosomal function. Mol Cell. 2017;66:517–32. 28525743 10.1016/j.molcel.2017.04.027 PMC5446411

[12] Zhao L, Wang YQ, Jaganathan A, Sun YF, Ma N, Li N, et al. BRD4-PRC2 represses transcription of T-helper 2-specific negative regulators during T-cell differentiation. EMBO J. 2023;42:16. 10.15252/embj.2022111473 PMC10015369 36719036

[13] Zhou D, Wu Z, Park JG, Fiches GN, Li TW, Ma Q, et al. FACT subunit SUPT16H associates with BRD4 and contributes to silencing of interferon signaling. Nucleic Acids Res. 2022;50:8700–18. 35904816 10.1093/nar/gkac645 PMC9410884

[14] Zhou XC, Mahdizadeh SJ, Le Gallo M, Eriksson LA, Chevet E, Lafont E. UFMylation: a ubiquitin-like modification. Trends Biochem Sci. 2024;49:52–67. 37945409 10.1016/j.tibs.2023.10.004

[15] Komatsu M, Inada T, Noda NN. The UFM1 system: working principles, cellular functions, and pathophysiology. Mol Cell. 2024;84:156–69. 38141606 10.1016/j.molcel.2023.11.034

[16] Jiang XJ, Stockwell BR, Conrad M. Ferroptosis: mechanisms, biology and role in disease. Nat Rev Mol Cell Biol. 2021;22:266–82. 33495651 10.1038/s41580-020-00324-8 PMC8142022

[17] Lei G, Zhuang L, Gan B. Targeting ferroptosis as a vulnerability in cancer. Nat Rev Cancer. 2022;22:381–96. 35338310 10.1038/s41568-022-00459-0 PMC10243716

[18] Rodriguez R, Schreiber SL, Conrad M. Persister cancer cells: iron addiction and vulnerability to ferroptosis. Mol Cell. 2022;82:728–40. 34965379 10.1016/j.molcel.2021.12.001 PMC9152905

[19] Wu N, Zheng B, Shaywitz A, Dagon Y, Tower C, Bellinger G, et al. AMPK-dependent degradation of TXNIP upon energy stress leads to enhanced glucose uptake via GLUT1. Mol Cell. 2013;49:1167–75. 23453806 10.1016/j.molcel.2013.01.035 PMC3615143

[20] Qiao S, Dennis M, Song X, Vadysirisack DD, Salunke D, Nash Z, et al. A REDD1/TXNIP pro-oxidant complex regulates ATG4B activity to control stress-induced autophagy and sustain exercise capacity. Nat Commun. 2015;6:7014. 25916556 10.1038/ncomms8014 PMC4421852

[21] Choi EH, Park SJ. TXNIP: a key protein in the cellular stress response pathway and a potential therapeutic target. Exp Mol Med. 2023;55:1348–56. 37394581 10.1038/s12276-023-01019-8 PMC10393958

[22] Zhou R, Tardivel A, Thorens B, Choi I, Tschopp J. Thioredoxin-interacting protein links oxidative stress to inflammasome activation. Nat Immunol. 2010;11:136–40. 20023662 10.1038/ni.1831

[23] Stoltzman CA, Kaadige MR, Peterson CW, Ayer DE. MondoA senses non-glucose sugars: regulation of thioredoxin-interacting protein (TXNIP) and the hexose transport curb. J Biol Chem. 2011;286:38027–34. 21908621 10.1074/jbc.M111.275503 PMC3207397

[24] Kaadige MR, Looper RE, Kamalanaadhan S, Ayer DE. Glutamine-dependent anapleurosis dictates glucose uptake and cell growth by regulating MondoA transcriptional activity. Proc Natl Acad Sci USA. 2009;106:14878–83. 19706488 10.1073/pnas.0901221106 PMC2736411

[25] Peterson CW, Stoltzman CA, Sighinolfi MP, Han KS, Ayer DE. Glucose controls nuclear accumulation, promoter binding, and transcriptional activity of the MondoA-Mlx heterodimer. Mol Cell Biol. 2010;30:2887–95. 20385767 10.1128/MCB.01613-09 PMC2876681

[26] Wu T, Kamikawa YF, Donohoe ME. Brd4’s bromodomains mediate histone H3 acetylation and chromatin remodeling in pluripotent cells through P300 and Brg1. Cell Rep. 2018;25:1756–71. 30428346 10.1016/j.celrep.2018.10.003

[27] Devaiah BN, Case-Borden C, Gegonne A, Hsu CH, Chen QR, Meerzaman D, et al. BRD4 is a histone acetyltransferase that evicts nucleosomes from chromatin. Nat Struct Mol Biol. 2016;23:540–8. 27159561 10.1038/nsmb.3228 PMC4899182

[28] Pan YB, Zhang QX, Tian L, Wang X, Fan XH, Zhang HH, et al. Jab1/CSN5 negatively regulates p27 and plays a role in the pathogenesis of nasopharyngeal carcinoma. Cancer Res. 2012;72:1890–900. 22350412 10.1158/0008-5472.CAN-11-3472 PMC3460549

[29] Jeon JH, Lee KN, Hwang CY, Kwon KS, You KH, Choi I. Tumor suppressor VDUP1 increases p27(kip1) stability by inhibiting JAB1. Cancer Res. 2005;65:4485–9. 15930262 10.1158/0008-5472.CAN-04-2271

[30] Soucy TA, Smith PG, Milhollen MA, Berger AJ, Langston SP. An inhibitor of NEDD8-activating enzyme as a new approach to treat cancer. Nature. 2009;458:732–6. 19360080 10.1038/nature07884

[31] Brownell JE, Micheal DS, Gavin JM, Liao H, Bruzzese FJ, Bump NJ, et al. Substrate-assisted inhibition of ubiquitin-like protein-activating enzymes: the NEDD8 E1 inhibitor MLN4924 forms a NEDD8-AMP mimetic in situ. Mol Cell. 2010;37:102–11. 20129059 10.1016/j.molcel.2009.12.024

[32] Loukil A, Cheung CT, Bendris N, Lemmers B, Peter M, Blanchard JM. Cyclin A2: at the crossroads of cell cycle and cell invasion. World J Biol Chem. 2015;6:346–50. 26629317 10.4331/wjbc.v6.i4.346 PMC4657123

[33] Cornwell JA, Crncec A, Afifi MM, Tang K, Amin R, Cappell SD. Loss of CDK4/6 activity in S/G2 phase leads to cell cycle reversal. Nature. 2023;619:363–70. 37407814 10.1038/s41586-023-06274-3 PMC10338338

[34] Mueller L, Gutschner T, Hatzfeld M. Going only half the way: cell cycle exit after the G1 restriction point. Signal Transduct Target Ther. 2023;8:2. 38040673 10.1038/s41392-023-01692-1 PMC10692125

[35] Bretones G, Delgado MD, León J. Myc and cell cycle control. Biochim Biophys Acta-Gene Regul Mech. 2015;1849:506–16. 10.1016/j.bbagrm.2014.03.013 24704206

[36] Lim TY, Wilde BR, Thomas ML, Murphy KE, Vahrenkamp JM, Conway ME, et al. TXNIP loss expands Myc-dependent transcriptional programs by increasing Myc genomic binding. PLoS Biol. 2023;21:29. 10.1371/journal.pbio.3001778 PMC10058090 36930677

[37] Tu WB, Shiah YJ, Lourenco C, Mullen PJ, Dingar D, Redel C, et al. MYC interacts with the G9a histone methyltransferase to drive transcriptional repression and tumorigenesis. Cancer Cell. 2018;34:579–95. 30300580 10.1016/j.ccell.2018.09.001

[38] Lourenco C, Resetca D, Redel C, Lin P, MacDonald AS, Ciaccio R, et al. MYC protein interactors in gene transcription and cancer. Nat Rev Cancer. 2021;21:579–91. 34188192 10.1038/s41568-021-00367-9

[39] Qin B, Yu J, Nowsheen S, Wang M, Tu X, Liu T, et al. UFL1 promotes histone H4 ufmylation and ATM activation. Nat Commun. 2019;10:1242. 30886146 10.1038/s41467-019-09175-0 PMC6423285

[40] McHugh D, Durán I, Gil J. Senescence as a therapeutic target in cancer and age-related diseases. Nat Rev Drug Discov. 2025;24:57–71. 39548312 10.1038/s41573-024-01074-4

[41] Blain SW, Scher HI, Cordon-Cardo C, Koff A. p27 as a target for cancer therapeutics. Cancer Cell. 2003;3:111–5. 12620406 10.1016/s1535-6108(03)00026-6

[42] Razavipour SF, Yoon H, Jang K, Kim M, Nawara HM, Bagheri A, et al. C-terminally phosphorylated p27 activates self-renewal driver genes to program cancer stem cell expansion, mammary hyperplasia and cancer. Nat Commun. 2024;15:18. 38886396 10.1038/s41467-024-48742-y PMC11183067

[43] Phan TG, Croucher PI. The dormant cancer cell life cycle. Nat Rev Cancer. 2020;20:398–411. 32488200 10.1038/s41568-020-0263-0

[44] La T, Chen S, Guo T, Zhao XH, Teng L, Li DD, et al. Visualization of endogenous p27 and Ki67 reveals the importance of a c-Myc-driven metabolic switch in promoting survival of quiescent cancer cells. Theranostics. 2021;11:9605–22. 34646389 10.7150/thno.63763 PMC8490506

[45] Ou Y, Wang SJ, Li D, Chu B, Gu W. Activation of SAT1 engages polyamine metabolism with p53-mediated ferroptotic responses. Proc Natl Acad Sci USA. 2016;113:E6806–12. 27698118 10.1073/pnas.1607152113 PMC5098629

[46] Guo ZY, Zhang W, Gao HX, Li Y, Li X, Yang XH, et al. High expression levels of haem oxygenase-1 promote ferroptosis in macrophage-derived foam cells and exacerbate plaque instability. Redox Biol. 2024;76:8. 10.1016/j.redox.2024.103345 PMC11414708 39255694

[47] Hong J, Li X, Hao Y, Xu H, Yu L, Meng Z, et al. The PRMT6/STAT1/ACSL1 axis promotes ferroptosis in diabetic nephropathy. Cell Death Differ. 2024;31:1561–75. 39134684 10.1038/s41418-024-01357-8 PMC11519485

[48] Deng JH, Pan T, Liu ZQ, McCarthy C, Vicencio JM, Cao LL, et al. The role of TXNIP in cancer: a fine balance between redox, metabolic, and immunological tumor control. Br J Cancer. 2023;129:1877–92. 37794178 10.1038/s41416-023-02442-4 PMC10703902

[49] Jia ZX, Zhang JY, Li ZJ, Ai LM. Identification of ferroptosis-related genes associated with diffuse large B-cell lymphoma via bioinformatics and machine learning approaches. Int J Biol Macromol. 2024;282:10. 10.1016/j.ijbiomac.2024.137117 39488307

[50] Verma N, Vinik Y, Saroha A, Nair NU, Ruppin E, Mills G, et al. Synthetic lethal combination targeting BET uncovered intrinsic susceptibility of TNBC to ferroptosis. Sci Adv. 2020;6:18. 10.1126/sciadv.aba8968 PMC7442484 32937365

[51] Vinik Y, Maimon A, Dubey V, Raj H, Abramovitch I, Malitsky S, et al. Programming a ferroptosis-to-apoptosis transition landscape revealed ferroptosis biomarkers and repressors for cancer therapy. Adv Sci. 2024;11:21. 10.1002/advs.202307263 PMC11077643 38441406

[52] Liu BB, Liu XH, Han LL, Chen X, Wu XD, Wu JJ, et al. BRD4-directed super-enhancer organization of transcription repression programs links to chemotherapeutic efficacy in breast cancer. Proc Natl Acad Sci USA. 2022;119:12. 10.1073/pnas.2109133119 PMC8832982 35105803

[53] Ran FA, Hsu PD, Wright J, Agarwala V, Scott DA, Zhang F. Genome engineering using the CRISPR-Cas9 system. Nat Protoc. 2013;8:2281–308. 24157548 10.1038/nprot.2013.143 PMC3969860

