# Supplementary figures and images for: BRD4 inhibition suppresses histone H4 UFMylation to increase ferroptosis sensitivity through TXNIP
Source: Cell Death Dis. 2025 Nov 17;16(1):843. doi: 10.1038/s41419-025-08166-y (PMC12623952; doi:10.1038/s41419-025-08166-y)

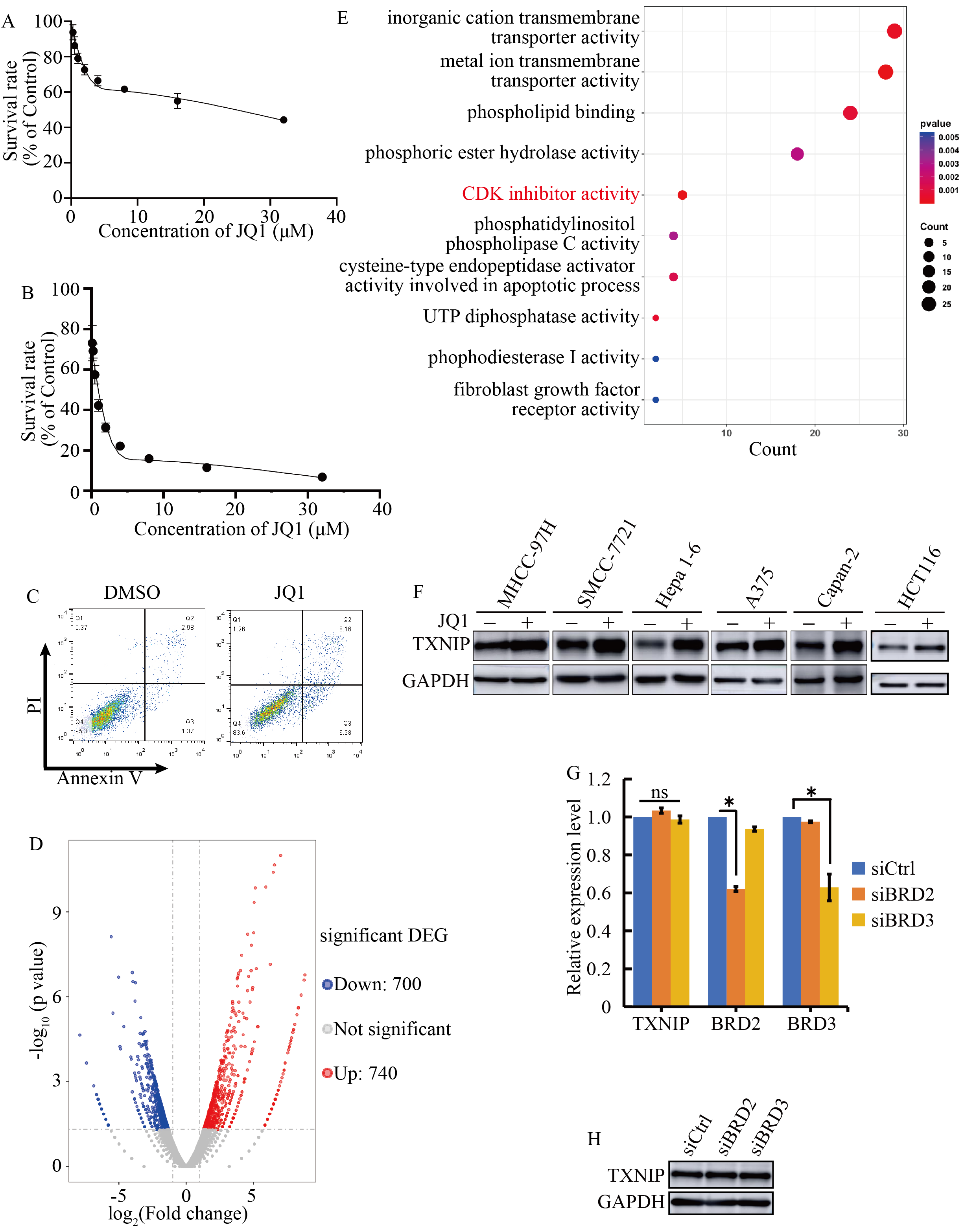

Supplement: Supplementary file 1 — supplemental figure 1 [file 41419_2025_8166_MOESM1_ESM.png]

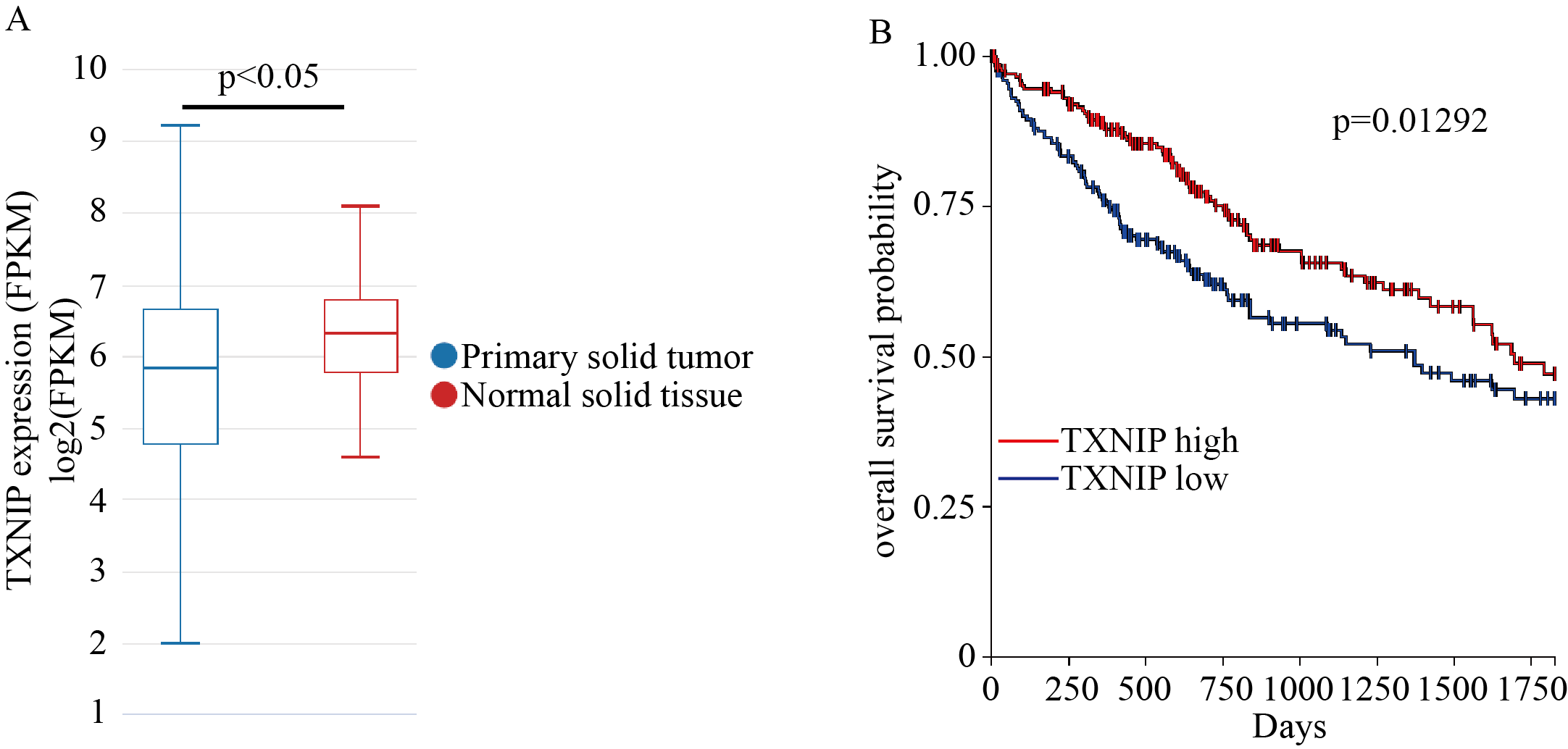

Supplement: Supplementary file 2 — supplemental figure 2 [file 41419_2025_8166_MOESM2_ESM.png]

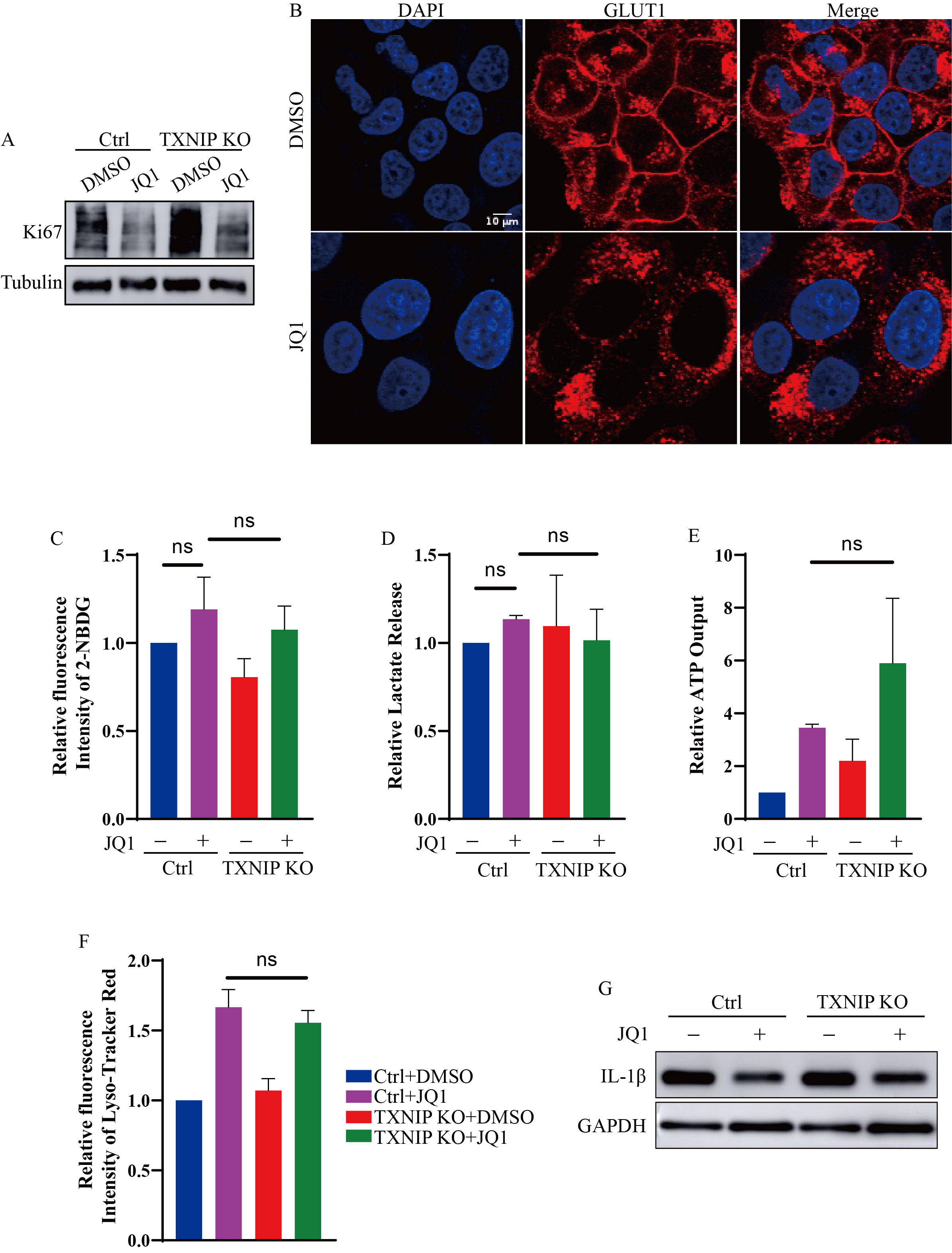

Supplement: Supplementary file 3 — supplemental figure 3 [file 41419_2025_8166_MOESM3_ESM.png]

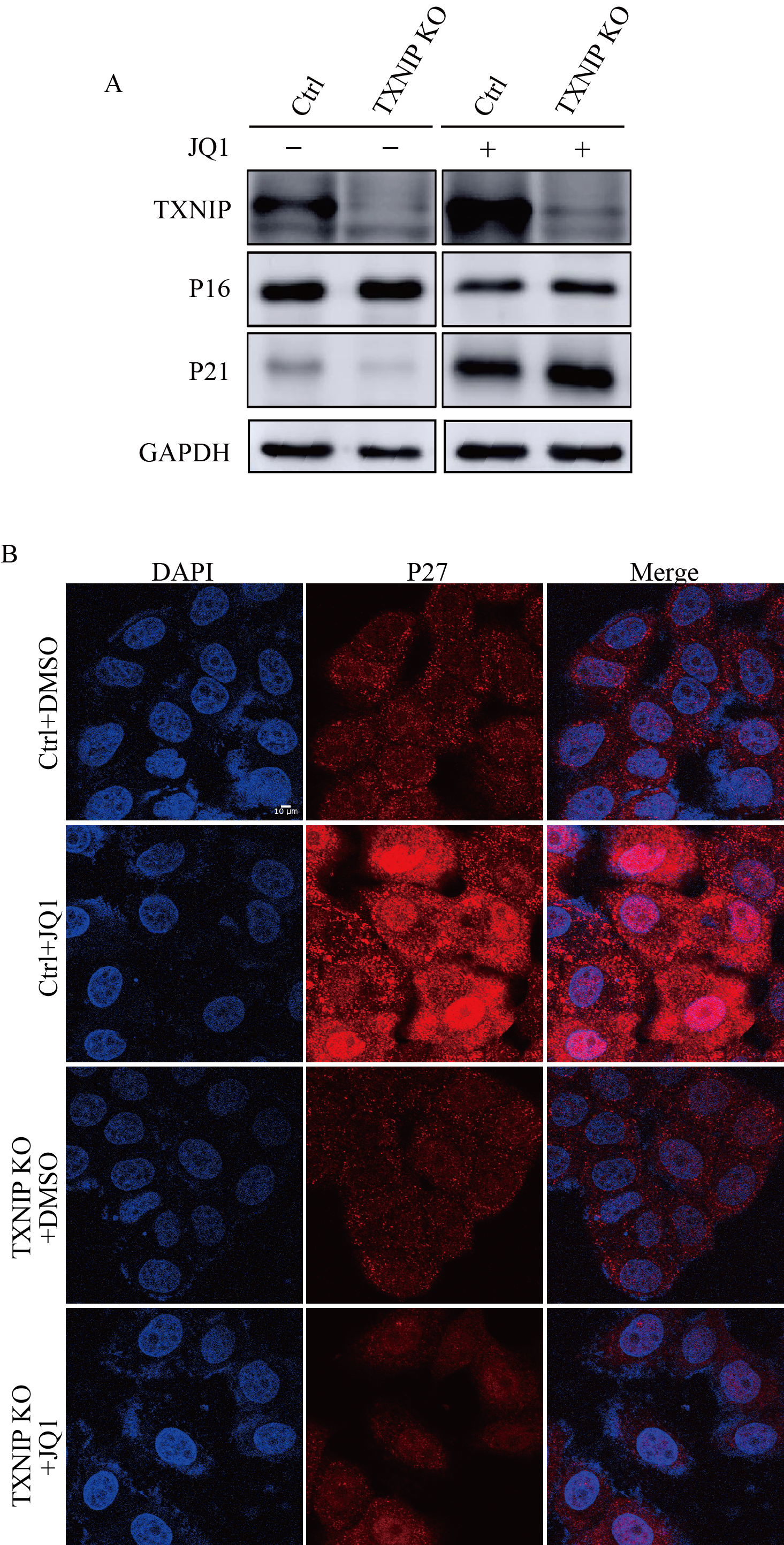

Supplement: Supplementary file 4 — supplemental figure 4 [file 41419_2025_8166_MOESM4_ESM.png]

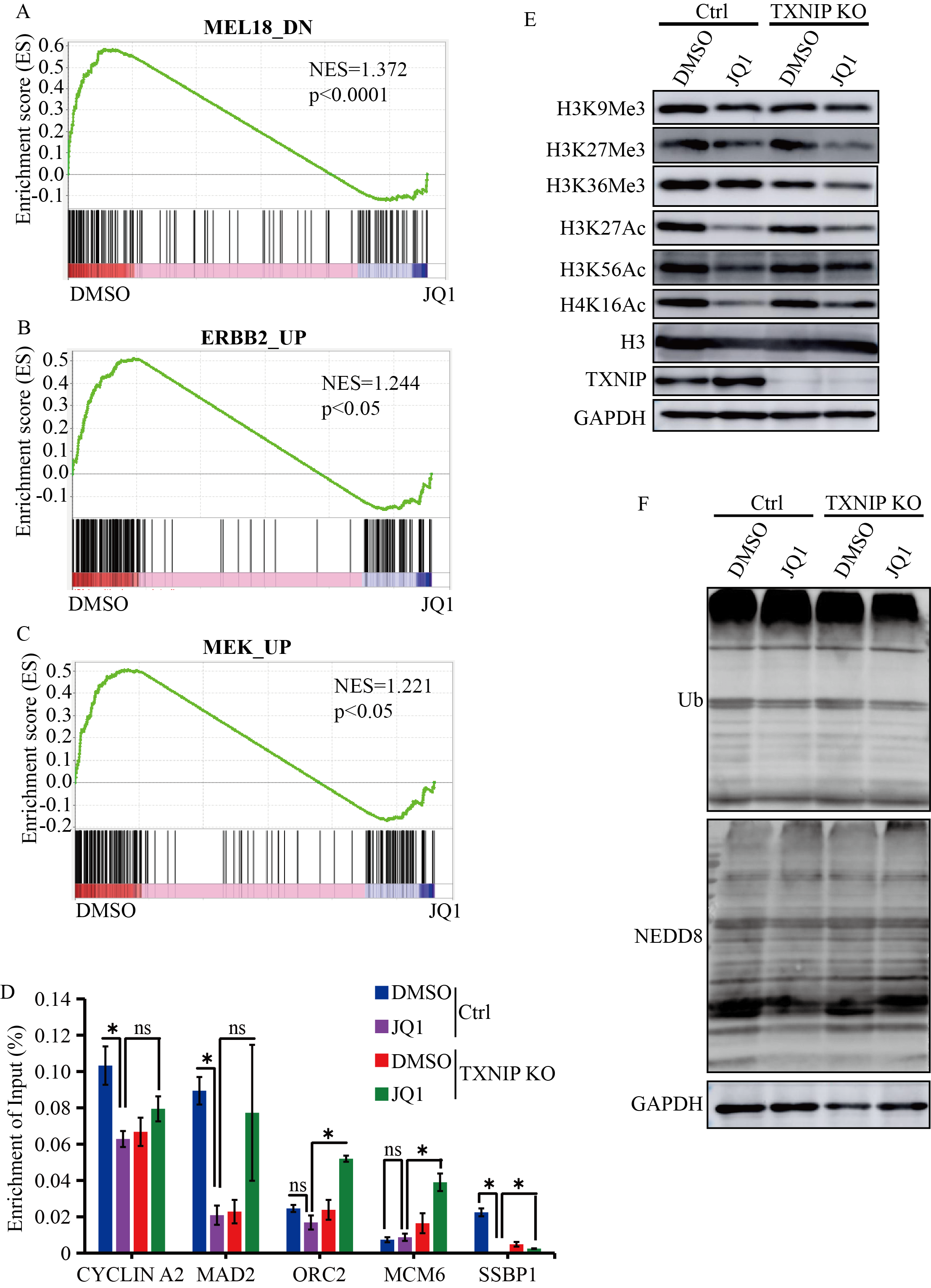

Supplement: Supplementary file 5 — supplemental figure 5 [file 41419_2025_8166_MOESM5_ESM.png]

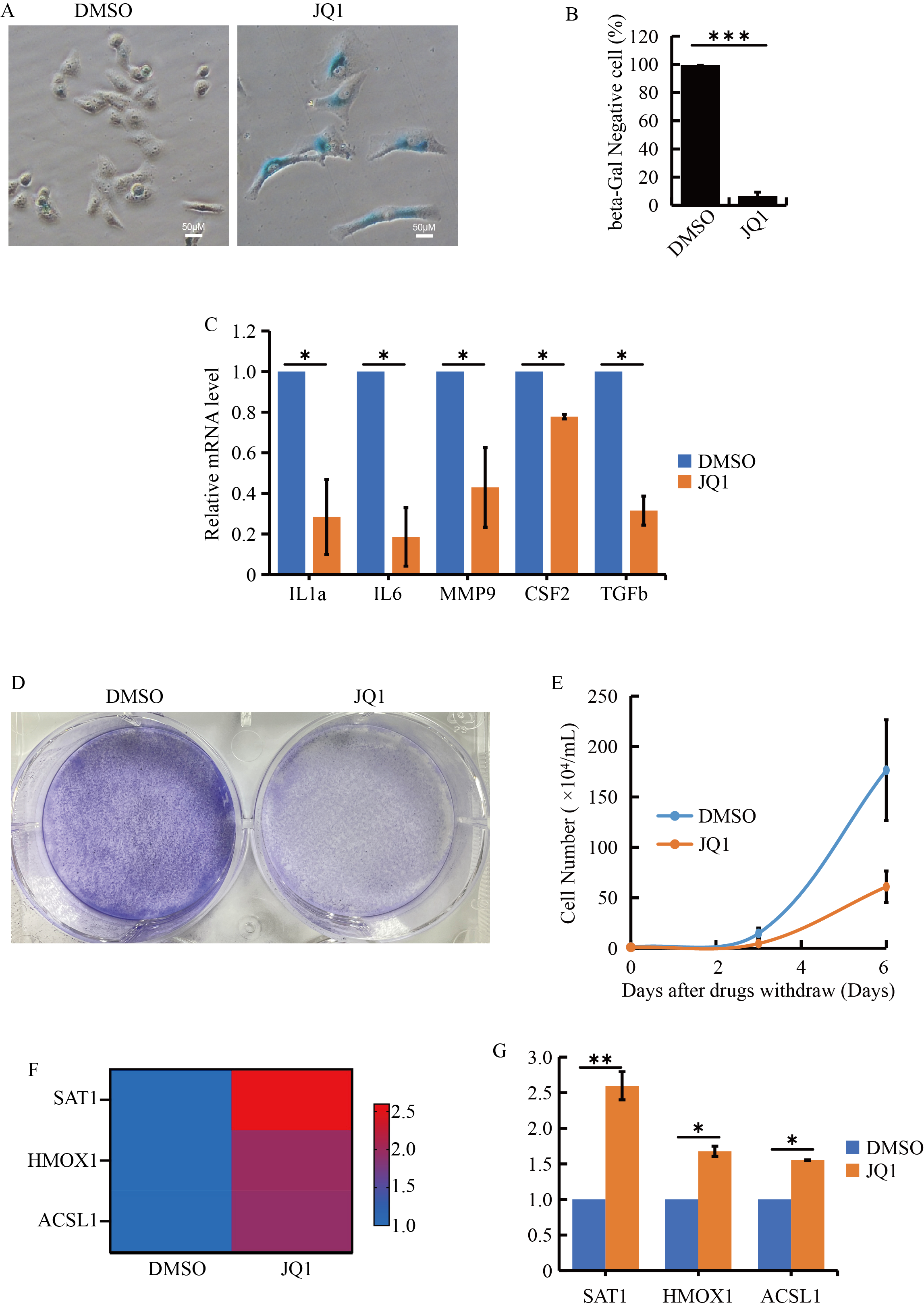

Supplement: Supplementary file 6 — supplemental figure 6 [file 41419_2025_8166_MOESM6_ESM.png]

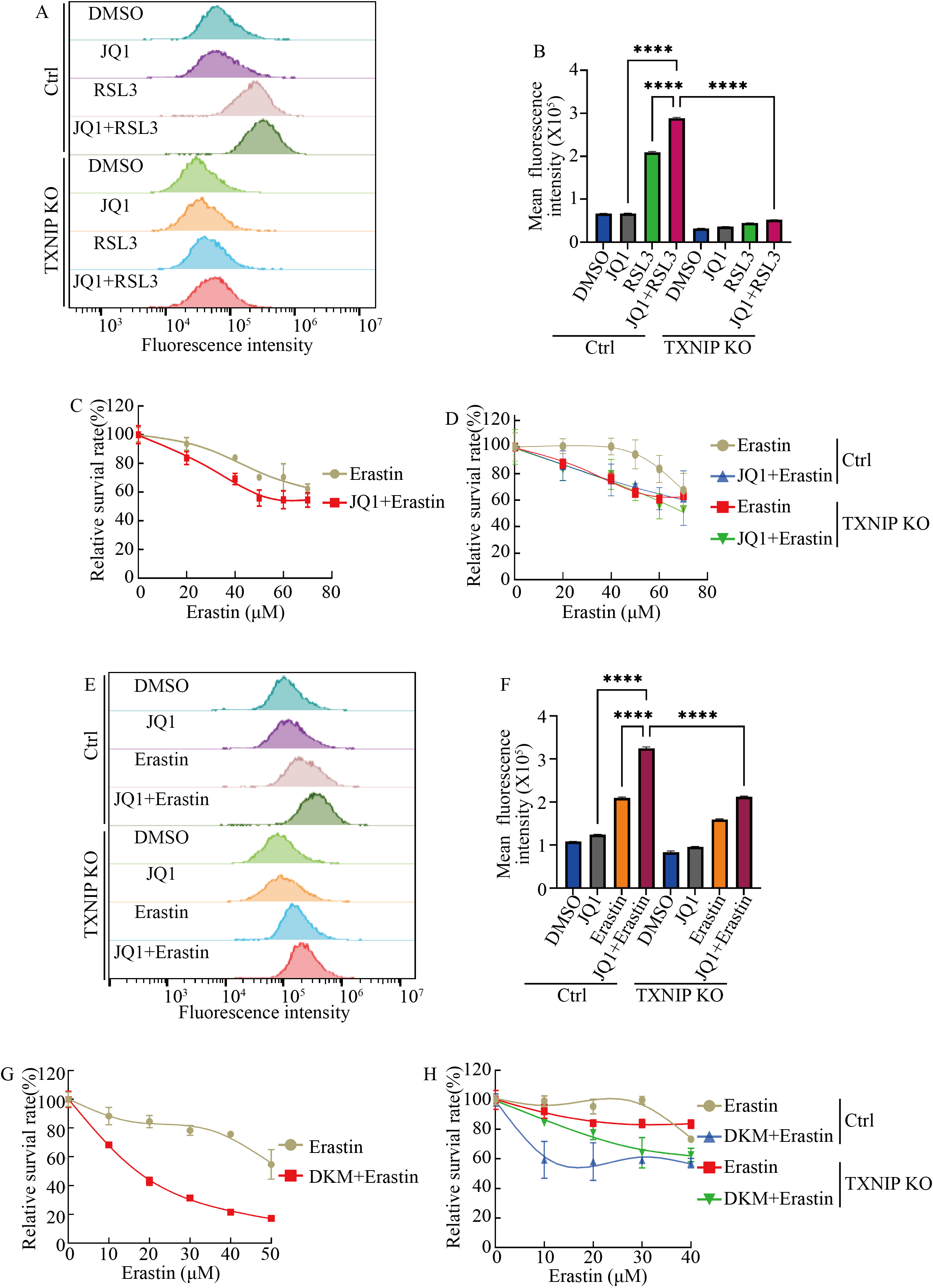

Supplement: Supplementary file 7 — supplemental figure 7 [file 41419_2025_8166_MOESM7_ESM.png]

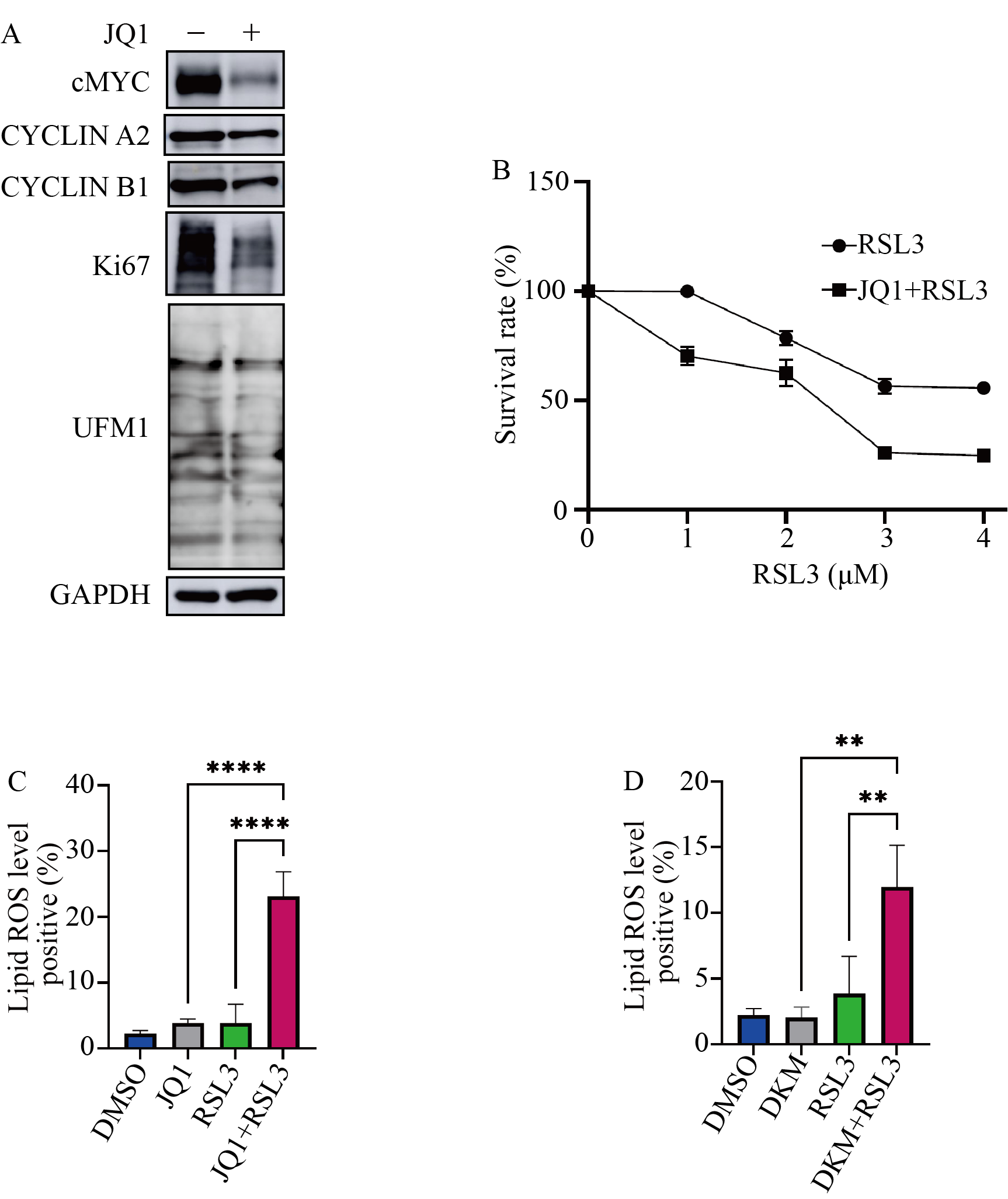

Supplement: Supplementary file 8 — supplemental figure 8 [file 41419_2025_8166_MOESM8_ESM.png]
