# Supplementary material for: BRD4 inhibition suppresses histone H4 UFMylation to increase ferroptosis sensitivity through TXNIP
Source: Cell Death Dis. 2025 Nov 17;16(1):843. doi: 10.1038/s41419-025-08166-y (PMC12623952; doi:10.1038/s41419-025-08166-y)
Supplement: Supplementary file 9 — supplemental figure legends [file 41419_2025_8166_MOESM9_ESM.docx]

**Supplementary** **Figure legends**

Supplementary Figure 1: JQ1 not only suppressed but also increased gene expression.

A&B: The survival rates of SMMC-7721 cells (A) and Hepa 1-6 cells (B) treated with varying concentrations of the BET inhibitor JQ1. C: Apoptosis analysis of HepG2 cells treated with 2 μM JQ1 for 48 hrs by flow cytometry. D: Inhibiting BRD4 increased gene expression. JQ1 increased 740 genes and suppressed 700 genes significantly. E: Gene Ontology (GO) analysis of JQ1-upregulated genes. F: TXNIP expression levels in MHCC-97H, SMMC-7721, Hepa 1-6, A375, Capan-2, and HCT116 cells treated with DMSO and 2 μM JQ1 for 48 hrs. G: The mRNA levels of TXNIP, BRD2 and BRD3 after knocking down BRD2 and BRD3. H: The protein level of TXNIP in cells with BRD2 and BRD3 knocked down. *p value<0.05, ns, not significant (two-tailed unpaired Student t test in G).

Supplementary Figure 2: The expression of TXNIP in human tumor and its association with patient prognosis.

A: TXNIP expression levels in primary tumor tissues and normal solid tissues. TXNIP mRNA level in TCGA pan-cancer database was analyzed. B: The five-year overall survival probability of liver cancer patients with high versus low TXNIP expression. TCGA liver cancer database was analyzed.

Supplementary Figure 3: The functions of TXNIP in HepG2 cells.

A: The level of Ki67 protein in Control and TXNIP KO cells treated with DMSO and 2 μM JQ1. B:The localization of GLUT1 in DMSO and 2 μM JQ1-treated HepG2 cells. Scale bar: 10 μm. C: Glucose uptake in Control and TXNIP KO cells after DMSO and JQ1 treatment. Glucose uptake was tracked by 2-NBDG. The fluorescent intensity of cellular 2-NBDG was measured using flow cytometry. D-G: The production of lactate (D), ATP concentration (E), lysosomal activity (F) and the expression of IL1β (G) in Control and TXNIP KO cells with DMSO and 2 μM JQ1 treatment. Lysosomal activity was assessed using Lysotracker red, and its fluorescent intensity was quantified using flow cytometry. Whole cell extracts were analyzed for IL1β expression using specific antibodies. ns, not significant (one-way ANOVA test in C-F).

Supplementary Figure 4: The effects of JQ1 on CDK inhibitors.

A: The expression of CDK inhibitors P16 and P21 in Control and TXNIP KO cells treated with DMSO and 2 μM JQ1. Whole cell extracts were probed with indicated antibodies. B: Immunostaining was performed using a P27 antibody in Control and TXNIP KO cells treated with DMSO and 2 μM JQ1 for 48 hrs. Scale bar: 10 μm.

Supplementary Figure 5: Histone modifications were altered by JQ1.

A-C: GSEA analysis for RNA-Seq data from cells treated with DMSO and 2 μM JQ1 indicated that MEL18 (A), ERBB2 (B), and MEK pathway (C) were downregulated in response to JQ1 treatment. D: The enrichment of E2F1 at the promoters of *CYCLIN A2*, *MAD2*, *ORC2*, *MCM6*, and *SSBP1* in Control and TXNIP KO cells with or without JQ1 treatment. E: The levels of H3K9Me3, H3K27Me3, H3K36Me3, H3K27Ac, H3K56Ac, and H4K16Ac in Control and TXNIP KO cells with or without 2 μM JQ1 treatment. F: The levels of total protein ubiquitination and Neddylation in Control and TXNIP KO cells treated with DMSO and 2 μM JQ1. Whole cell extracts were prepared and immunoblotted with indicated antibodies. *p value<0.05, ns, not significant (one-way ANOVA test in D).

Supplementary Figure 6: Cells entered a dormant state upon JQ1 treatment.

A&B: Beta-Gal staining assay in cells treated with DMSO and 2 μM JQ1 for 7 days. The ratio of beta-Gal positive cells in DMSO and JQ1 treated cells was shown in B. Scale bar: 50 μm. C: The mRNA levels of *IL1ɑ*, *IL6*, *MMP9*, *CSF2* and *TGFb* in DMSO and JQ1 treated cells was determined by RT-PCR. Cells were treated with DMSO and 2 μM JQ1 for 7 days. D: JQ1-treated cells re-grew after JQ1 withdrawal. Cells were treated with DMSO and 2 μM JQ1 for 7 days, and then these chemicals were withdrawn. Crystal staining was carried out 7 days after JQ1 withdrawal. E: Cell growth curve of DMSO- and JQ1-treated cells after withdrawing DMSO and JQ1. F: Heatmap of fold change (log2 scale) in mRNA levels of *SAT1*, *HMOX1*, and *ACSL1* between DMSO and JQ1 treated cells according to the RNA-seq data. G: The transcriptional levels of *SAT1*, *HMOX1*, and *ACSL1* after JQ1 treatment was validated by RT-qPCR. ***p value<0.005, **p value<0.01, *p value<0.05 (two-tailed unpaired Student t test in B, C and G).

Supplementary Figure 7: Protein UFMylation regulated cell sensitivity to ferroptosis inducers.

A&B: ROS production in Control and TXNIP KO cells treated with 2 μM JQ1, 6 μM RSL3, and a combination of 2 μM JQ1 with 6 μM RSL3. Quantitative analysis of ROS amount was shown in B. C: The survival rates of cells exposed to increasing concentrations of Erastin alone or combined with 2 μM JQ1. D: The survival rates of Control and TXNIP KO cells treated with increasing amount of Erastin alone or combined with 2 μM JQ1. E&F: ROS production in Control and TXNIP KO cells treated with 2 μM JQ1, 60 μM Erastin, and a combination of 2 μM JQ1 with 60 μM Erastin. Quantitative analysis of ROS concentration was shown in F. G: The survival rates of cells treated with increasing concentration of Erastin alone or combined with 30 μM DKM 2-93. H: The survival rates of Control and TXNIP KO cells treated with increasing concentration of Erastin alone or combined with DKM 2-93. ****p value<0.001 (one-way ANOVA test in B&F).

Supplementary Figure 8: JQ1 increased sensitivity to ferroptosis in HCT116 cells.

A. The protein levels of cMYC, CYCLIN A2, CYCLIN B1, Ki67, and protein UFMylation in HCT116 cells following treatment with JQ1. HCT116 cells were treated with DMSO and 2 μM JQ1 for 48 hrs. B. The survival rates of HCT116 cells treated with increasing concentration of RSL3 alone or combined with 2 μM JQ1. C. The level of lipid ROS in HCT116 cells treated with DMSO, 2 μM JQ1, 6 μM RSL3, and JQ1 (2 μM)+RSL3 (6 μM). D. The level of lipid ROS in HCT116 cells treated with DMSO, 30 μM DKM 2-93, 6 μM RSL3, and DKM 2-93 (30 μM)+RSL3 (6 μM).****p value<0.001, **p value<0.01 (one-way ANOVA test in C&D).
