# Supplementary material for: BRD4 inhibition suppresses histone H4 UFMylation to increase ferroptosis sensitivity through TXNIP
Source: Cell Death Dis. 2025 Nov 17;16(1):843. doi: 10.1038/s41419-025-08166-y (PMC12623952; doi:10.1038/s41419-025-08166-y)
Supplement: Supplementary file 10 — supplemental table 1 [file 41419_2025_8166_MOESM10_ESM.docx]

Supplementary Table 1: Primers for RT-PCR

| Primer Name | Sequence (5’ to3’) |
| --- | --- |
| hu*TXNIP*For | ATATGGGTGTGTAGACTACTGGG |
| hu*TXNIP*Rev | GACATCCACCAGATCCACTACT |
| hu*MYC*For | GTCAAGAGGCGAACACACAAC |
| hu*MYC*Rev | TTGGACGGACAGGATGTATGC |
| hu*P27*For | ATCACAAACCCCTAGAGGGCA |
| hu*P27*Rev | GGGTCTGTAGTAGAACTCGGG |
| hu*IL-1β*For | CTCTCTCCTTTCAGGGCCAA |
| hu*IL-1β*Rev | GAGAGGCCTGGCTCAACAAA |
| hu*CSF2*For | TCCTGAACCTGAGTAGAGACAC |
| hu*CSF2*Rev | TGCTGCTTGTAGTGGCTGG |
| hu*TNF-α*For | GAGGCCAAGCCCTGGTATG |
| hu*TNF-α*Rev | CGGGCCGATTGATCTCAGC |
| hu*CXCL10*For | GTGGCATTCAAGGAGTACCTC |
| hu*CXCL10*Rev | TGATGGCCTTCGATTCTGGATT |
| hu*IL18*For | TCTTCATTGACCAAGGAAATCGG |
| hu*IL18*Rev | TCCGGGGTGCATTATCTCTAC |
| hu*TGF-β*For | CAATTCCTGGCGATACCTCAG |
| hu*TGF-β*Rev | GCACAACTCCGGTGACATCAA |
| hu*Cyclin A1*For | GAGGTCCCGATGCTTGTCAG |
| hu*Cyclin A1*Rev | GTTAGCAGCCCTAGCACTGTC |
| hu*Cyclin A2*For | GGATGGTAGTTTTGAGTCACCAC |
| hu*Cyclin A2*Rev | CACGAGGATAGCTCTCATACTGT |
| hu*Cyclin B1*For | AATAAGGCGAAGATCAACATGGC |
| hu*Cyclin B1*Rev | TTTGTTACCAATGTCCCCAAGAG |
| hu*Cyclin D1*For | CAATGACCCCGCACGATTTC |
| hu*Cyclin D1*Rev | CATGGAGGGCGGATTGGAA |
| hu*Cyclin D2*For | TTTGCCATGTACCCACCGTC |
| hu*Cyclin D2*Rev | AGGGCATCACAAGTGAGCG |
| hu*Cyclin E1*For | GCCAGCCTTGGGACAATAATG |
| hu*Cyclin E1*Rev | CTTGCACGTTGAGTTTGGGT |
| hu*MAD2L1*For | GGACTCACCTTGCTTGTAACTAC |
| hu*MAD2L1*Rev | GATCACTGAACGGATTTCATCCT |
| hu*ORC2*For | GCTTCAGACAAGGTTCAACCG |
| hu*ORC2*Rev | CTGTGCAACCCCTTCATCATC |
| hu*MCM6*For | TCGGGCCTTGAAAACATTCGT |
| hu*MCM6*Rev | TGTGTCTGGTAGGCAGGTCTT |
| hu*SSBP1*Rev | ACTGGGTGATGTCAGTCAAAAG |
| hu*SSBP1*Rev | TGCTTGTCGCCTCACATTATT |
| hs*TXNIP*chipFor | CCACAGCGATCTCACTGATTG |
| hs*TXNIP*chipRev | AGGAGGCGGAAACGTCTCTAT |
| hs*Cyclin A2*chipFor | GACAGGGTCGCAGGCGAGTGA |
| hs*Cyclin A2*chipRev | CCAGCCAGTTTGTTTCTCCCTC |
| hs*MAD2L1*chipFor | CCGAGGGAAGAAGATGACGAT |
| hs*MAD2L1*chipRev | CGAACAGTTCCTTTCAGTTTGA |
| hs*ORC2*chipFor | GGAAACCTGAGGAAAACGGA |
| hs*ORC2*chipRev | GAAACGGAAAGAAGAAAGGAAA |
| hs*MCM6*chipFor | GGAACCAATCGTGACACAGGA |
| hs*MCM6*chipRev | CGTGGGCTGGGGCTCTTGGA |
