# Supplementary material for: BRD4 inhibition suppresses histone H4 UFMylation to increase ferroptosis sensitivity through TXNIP
Source: Cell Death Dis. 2025 Nov 17;16(1):843. doi: 10.1038/s41419-025-08166-y (PMC12623952; doi:10.1038/s41419-025-08166-y)

Fig1 E

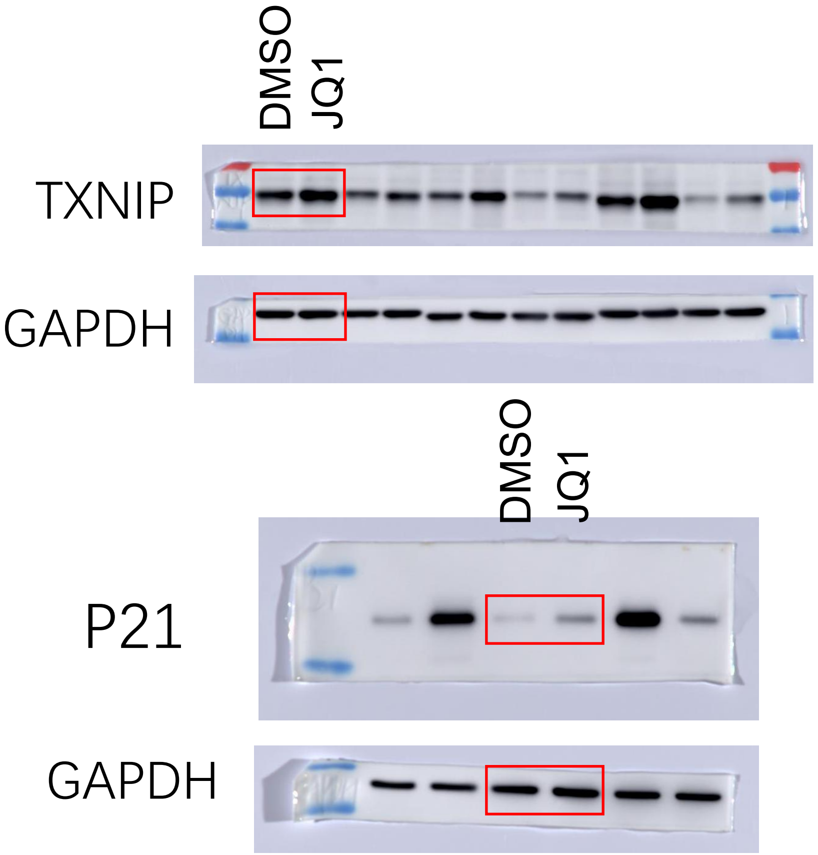

Fig1 G

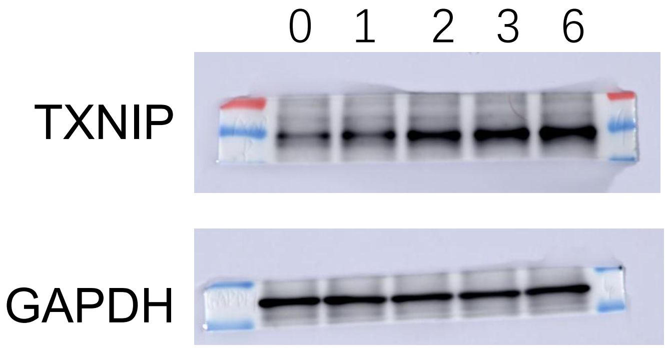

Fig1 J

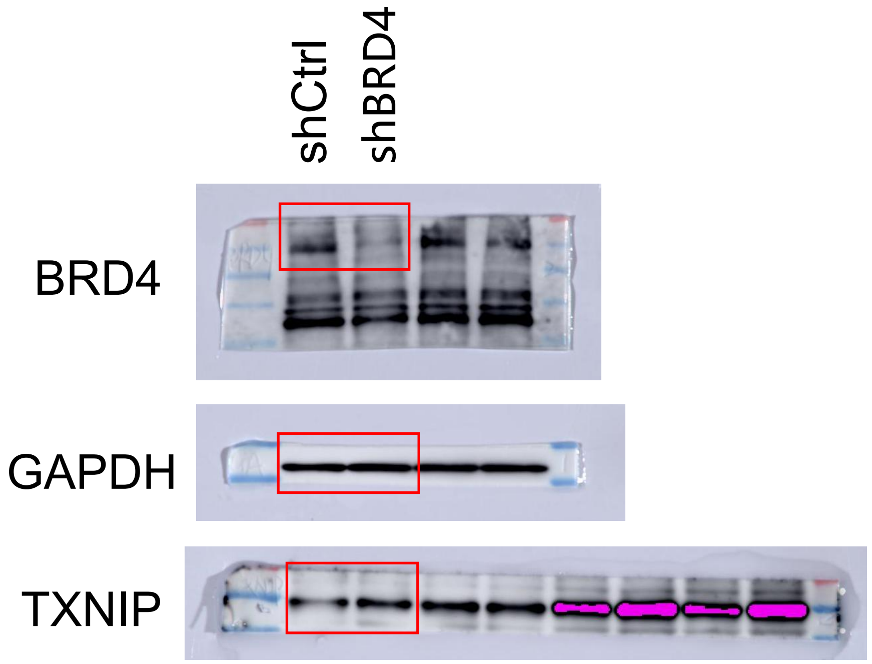

Fig2 A

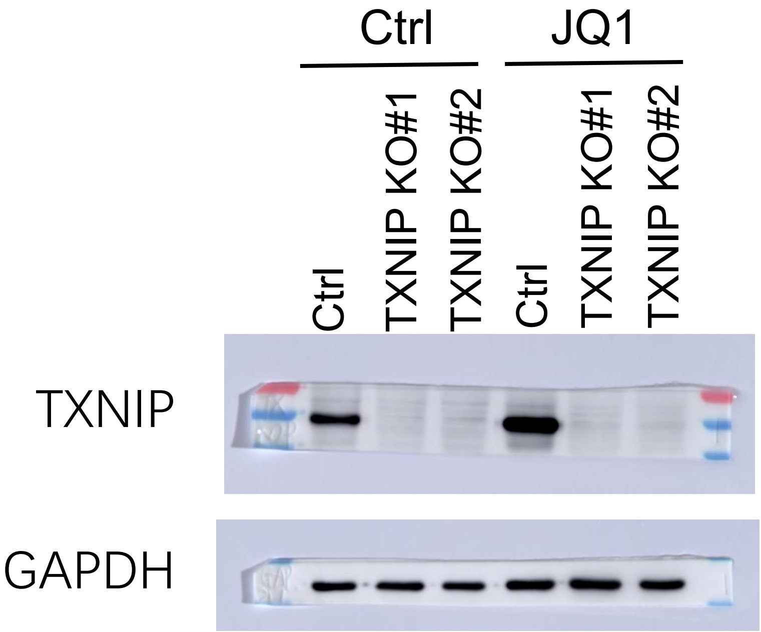
Ctrl
JQ1
Ctrl
TXNIP KO#1
TXNIP KO#2
Ctrl
TXNIP KO#1
TXNIP KO#2
TXNIP
GAPDH

Fig3 A

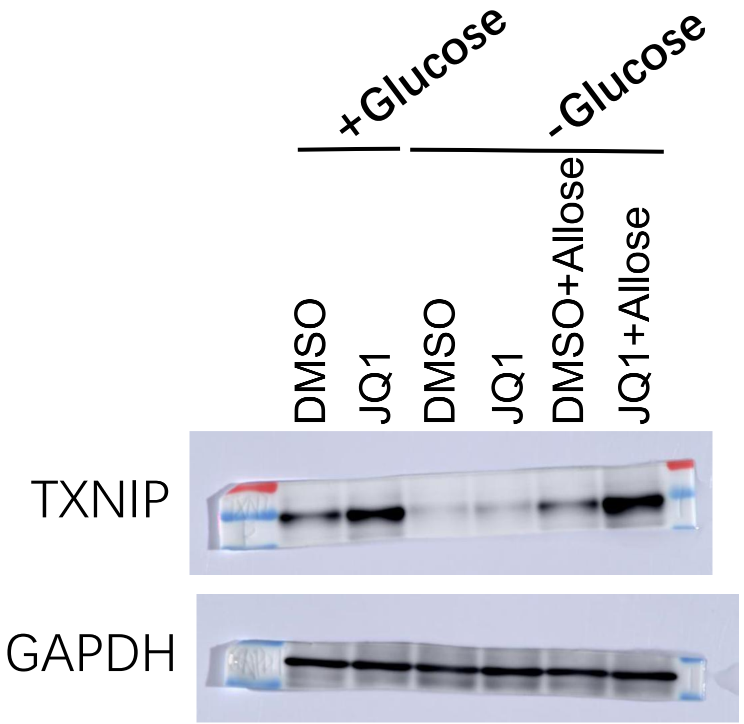
+Glucose
-Glucose
DMSO
JQ1
DMSO
JQ1
DMSO+Allose
JQ1+Allose
TXNIP
GAPDH

Fig3 D

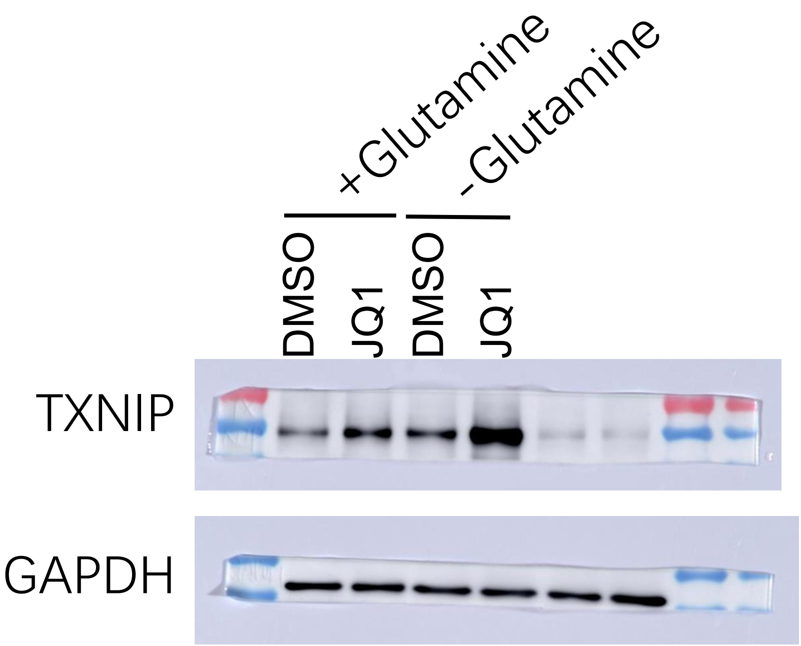

Fig 3E

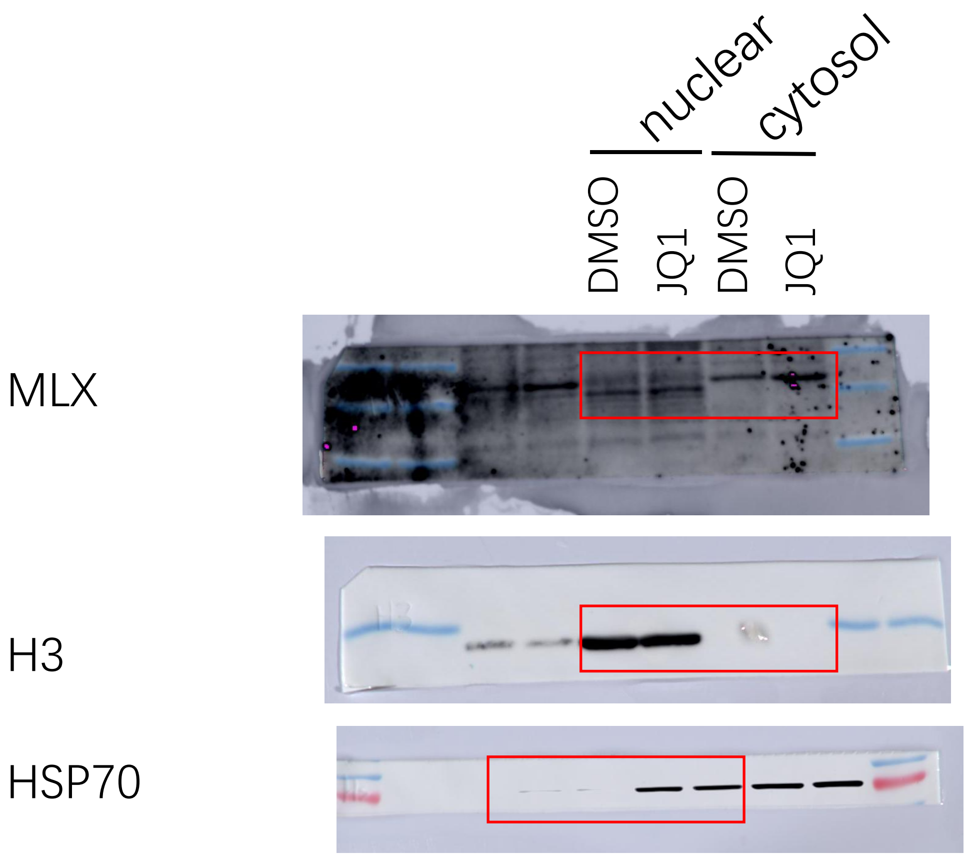

Fig3 G

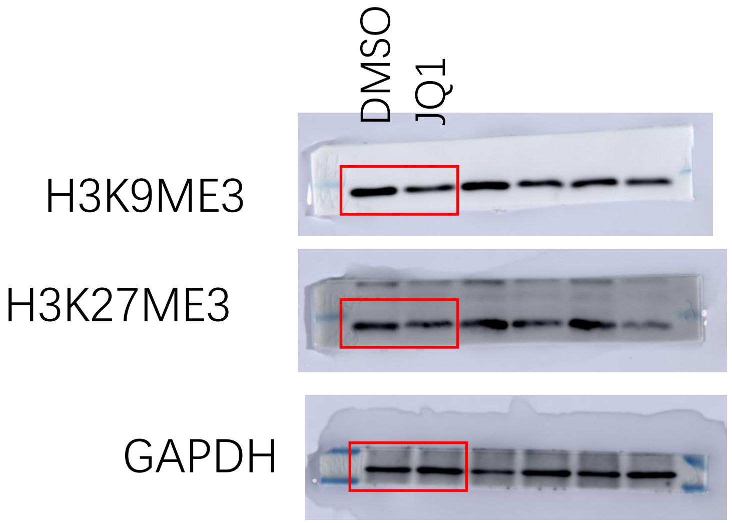

Fig3 I

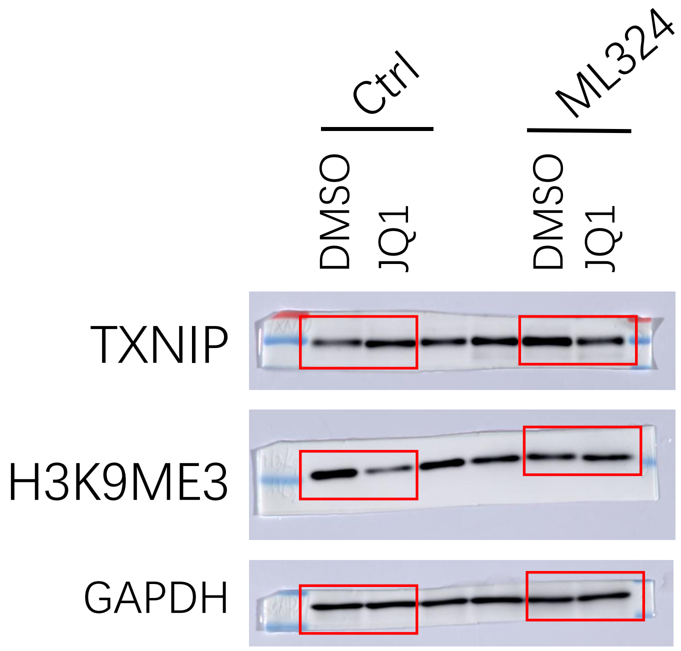

Fig4 A

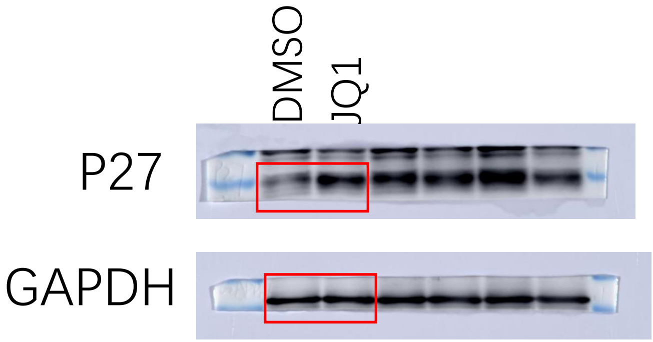

Fig4 B

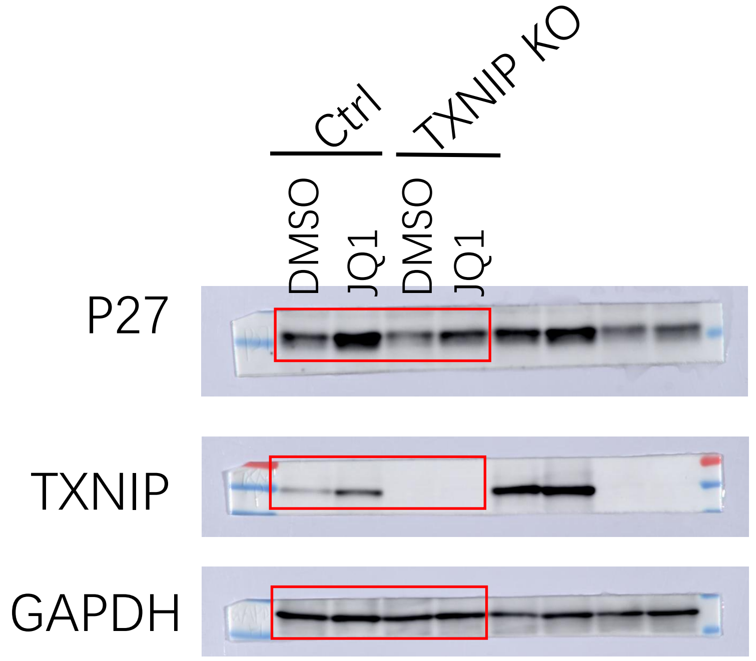

Fig4 D

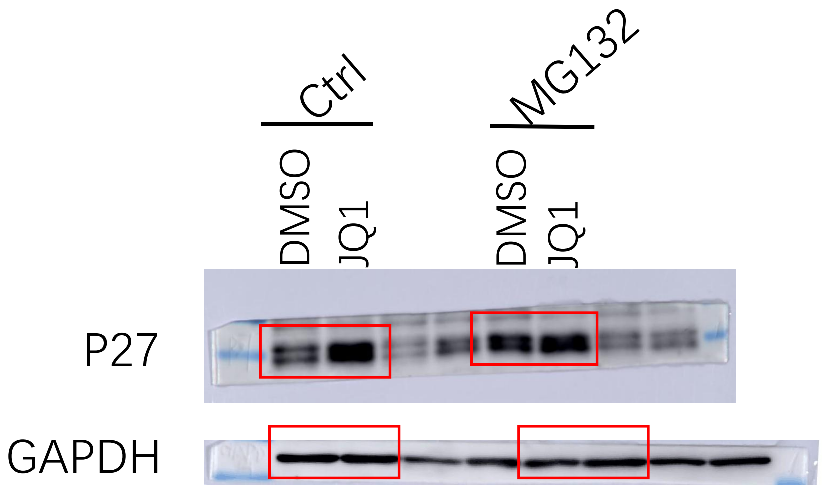

Fig4 E

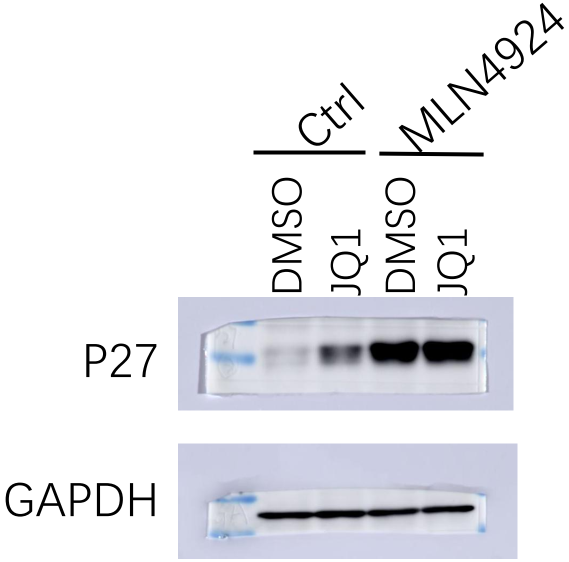

Fig4 F

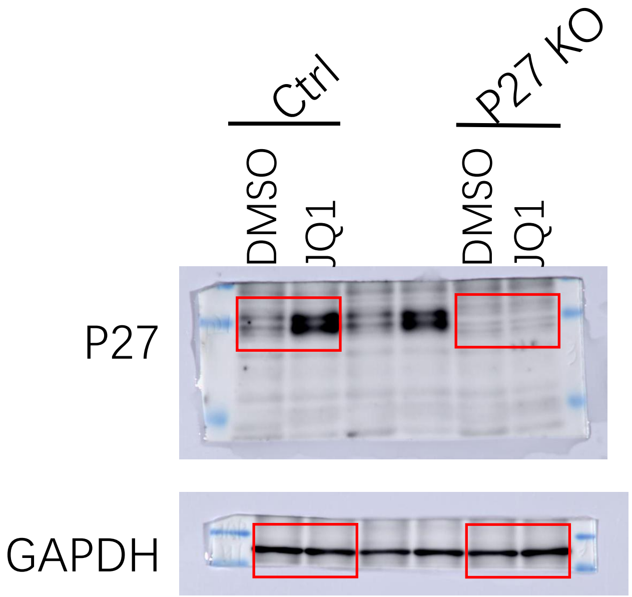

Fig5 B

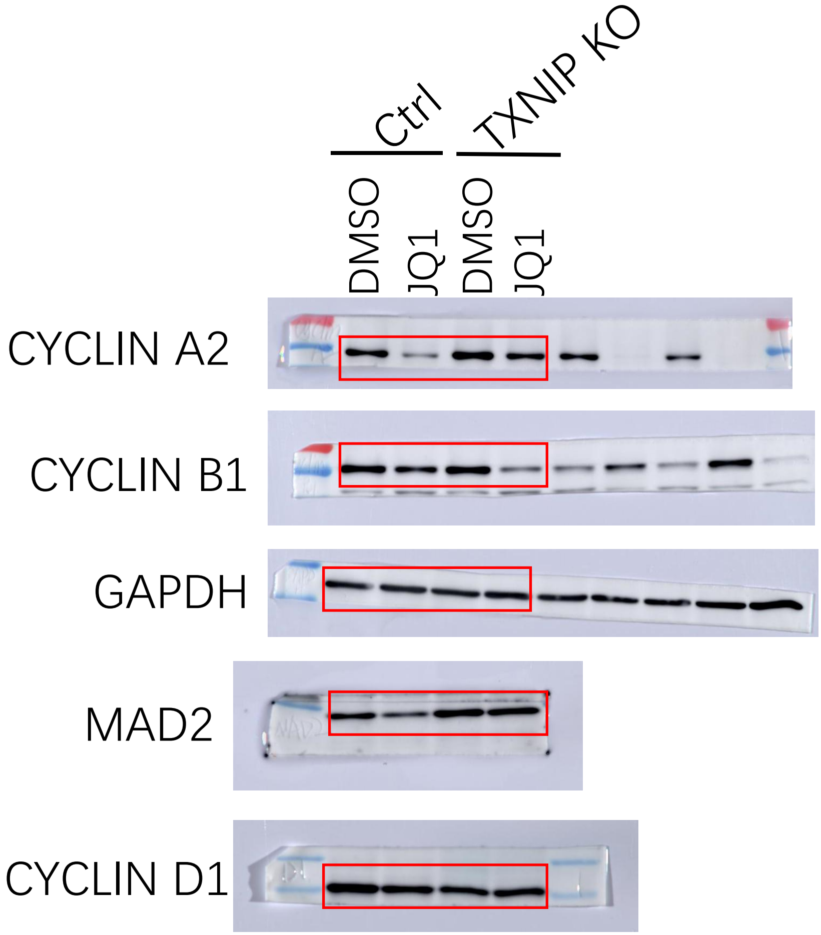
Ctrl
TXNIP KO
DMSO
JQ1
DMSO
JQ1
CYCLIN A2
CYCLIN B1
GAPDH
MAD2
CYCLIN D1

Fig5 E

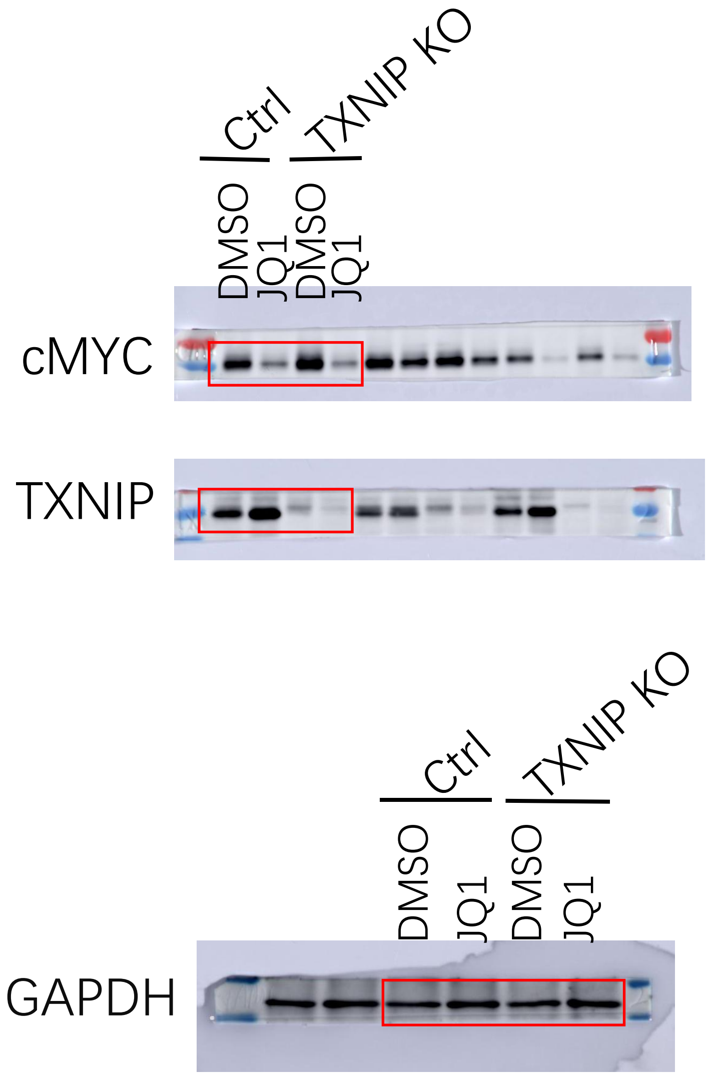
Ctrl
TXNIP KO
DMSO
JQ1
DMSO
JQ1
cMYC
TXNIP
Ctrl
TXNIP KO
DMSO
JQ1
DMSO
JQ1
GAPDH

Fig 5F

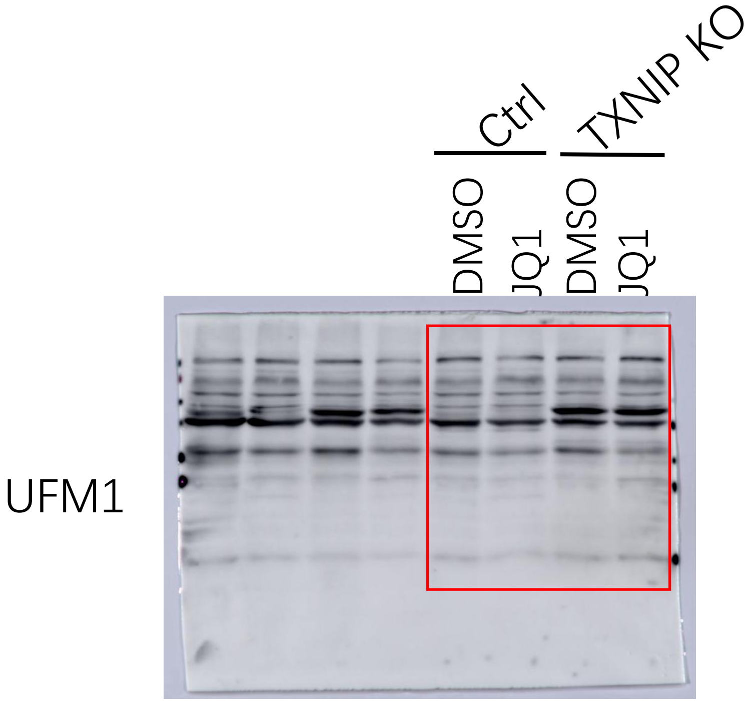

Ctrl
TXNIP KO
DMSO
JQ1
DMSO
JQ1
UFM1

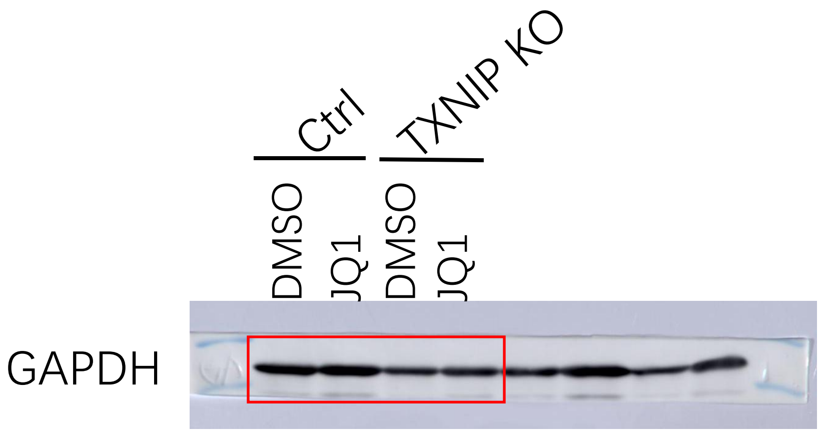

Ctrl
TXNIP KO
DMSO
JQ1
DMSO
JQ1
GAPDH

Fig 5H

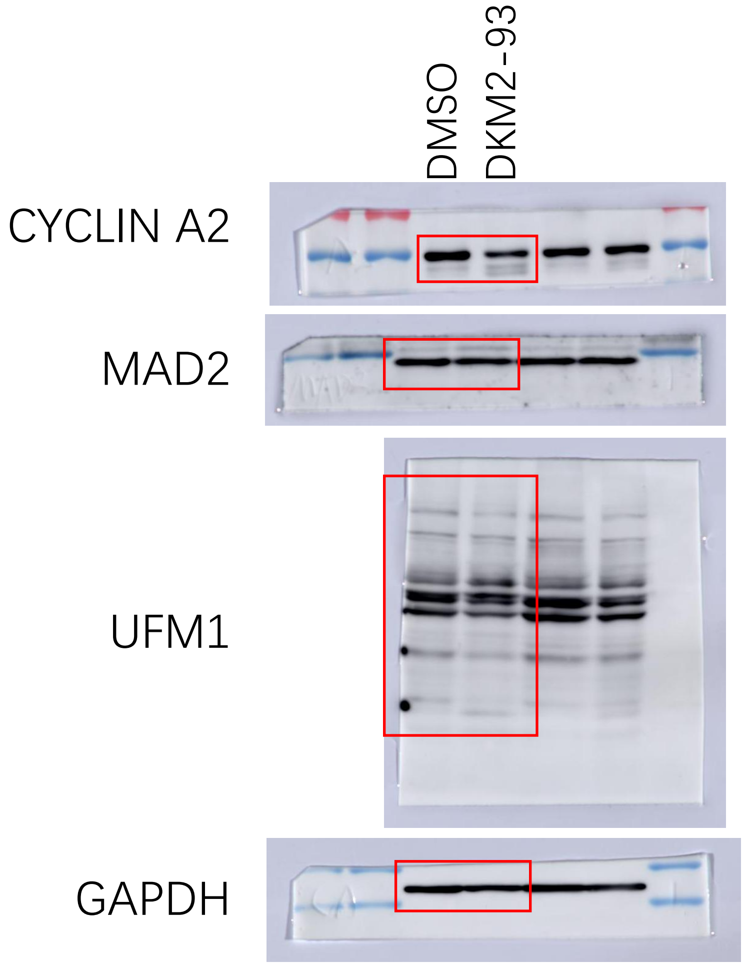

Fig6 A

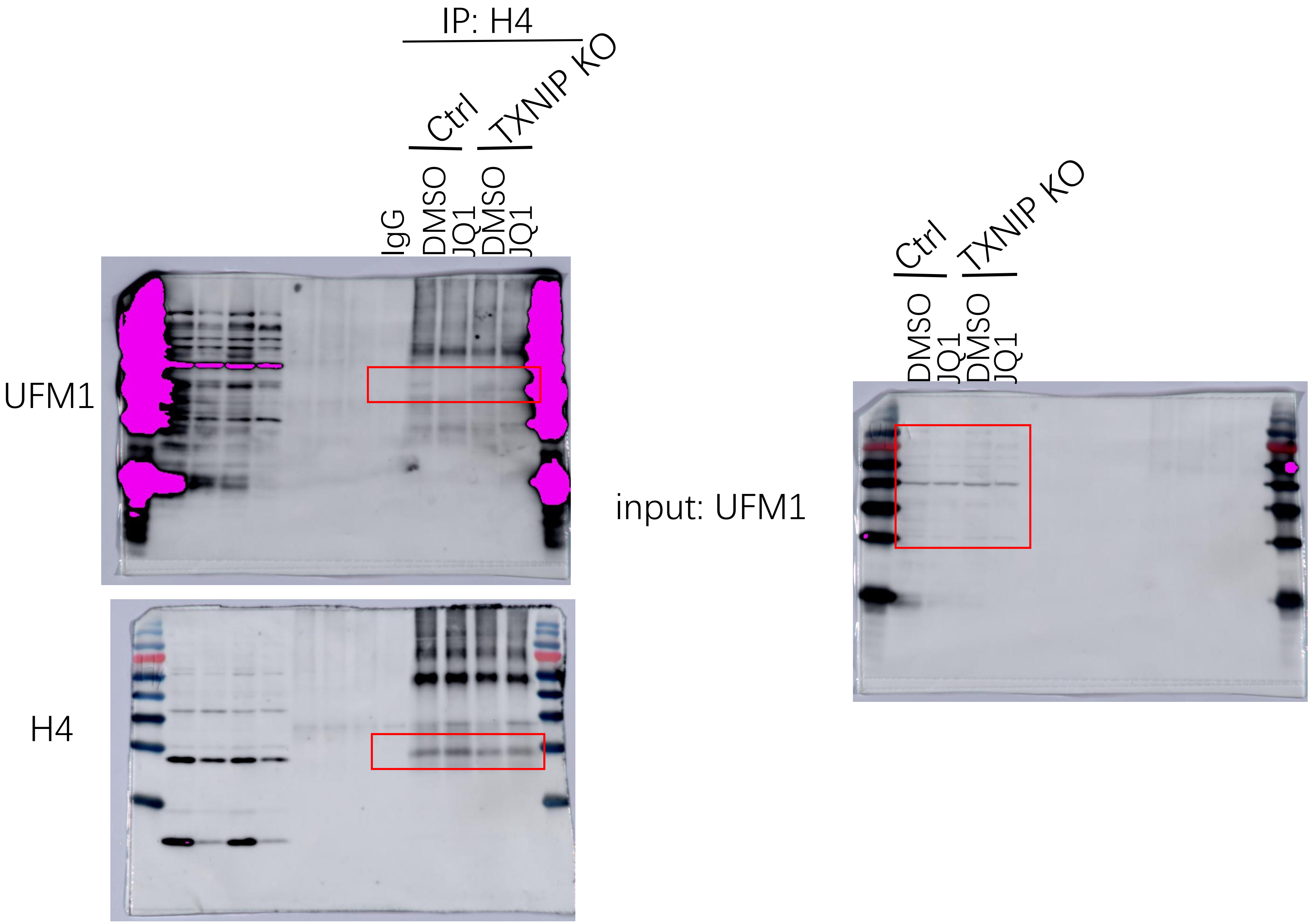
IP: H4
Ctrl
TXNIP KO
IgG
DMSO
JQ1
DMSO
JQ1
UFM1
H4
Ctrl
TXNIP KO
DMSO
JQ1
DMSO
JQ1
input: UFM1

Fig6 B

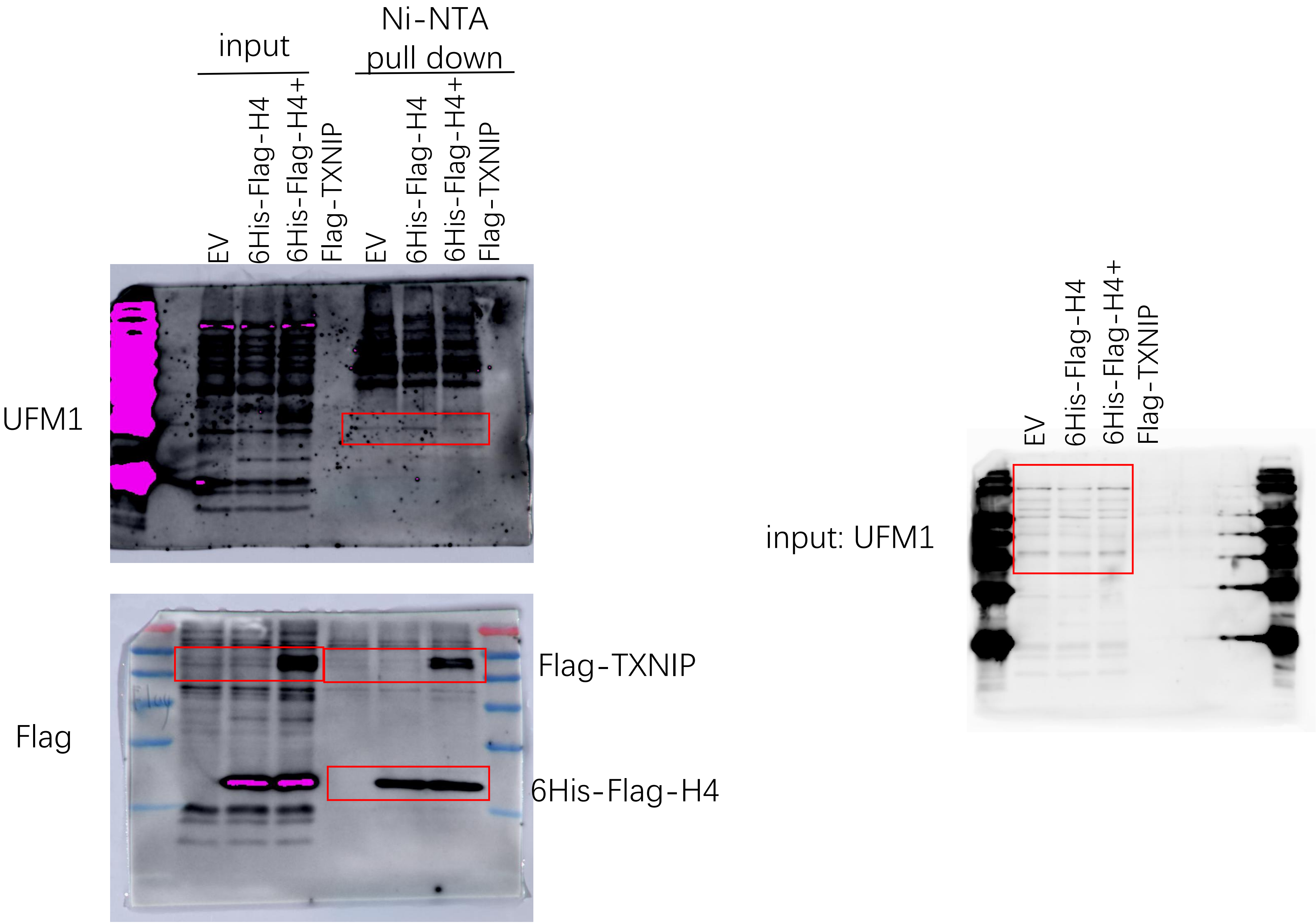

input
Ni-NTA pull down
EV
6His-Flag-H4
6His-Flag-H4+
Flag-TXNIP
EV
6His-Flag-H4
6His-Flag-H4+
Flag-TXNIP
UFM1
Flag
Flag-TXNIP
6His-Flag-H4
EV
6His-Flag-H4
6His-Flag-H4+
Flag-TXNIP
input: UFM1

Fig 6C

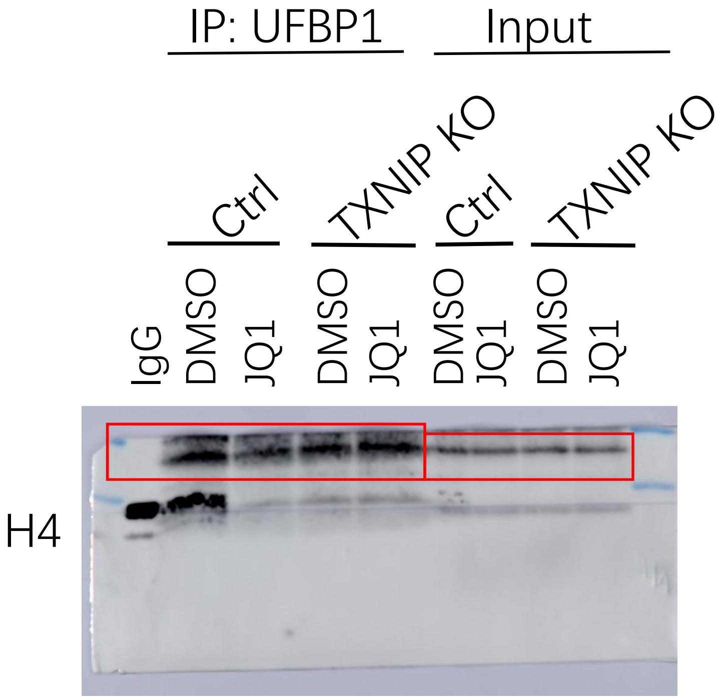
IP: UFBP1
Input
Ctrl
TXNIP KO
Ctrl
TXNIP KO
IgG
DMSO
JQ1
DMSO
JQ1
DMSO
JQ1
DMSO
JQ1
H4

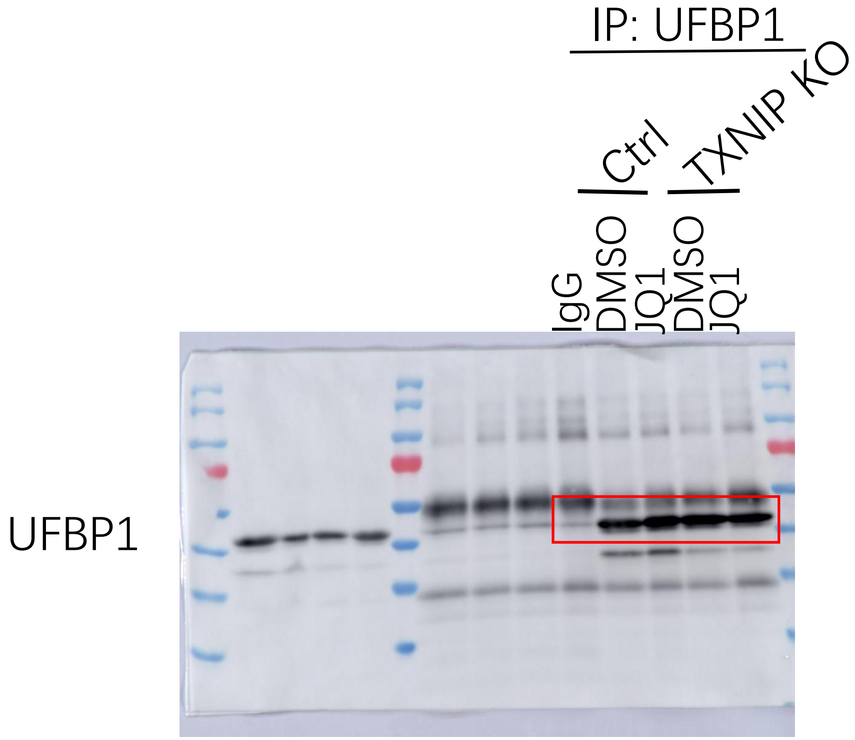
IP: UFBP1
Ctrl
TXNIP KO
IgG
DMSO
JQ1
DMSO
JQ1
UFBP1

Fig 6D

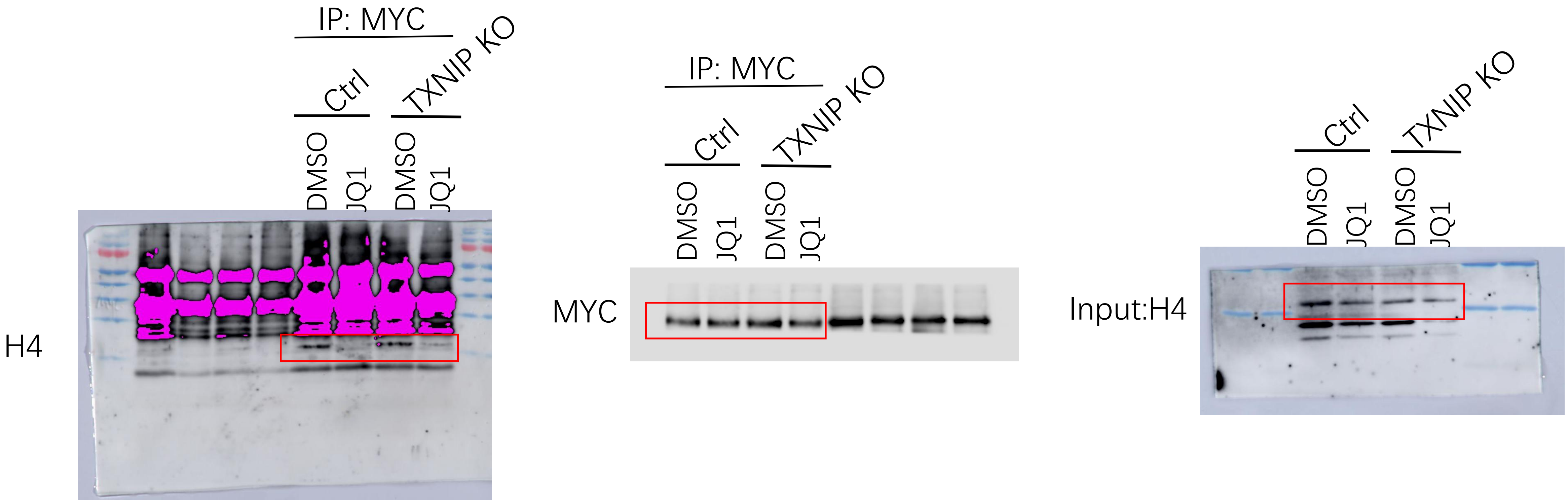

IP: MYC
Ctrl
TXNIP KO
DMSO
JQ1
DMSO
JQ1
H4
IP: MYC
Ctrl
TXNIP KO
DMSO
JQ1
DMSO
JQ1
MYC
Ctrl
TXNIP KO
DMSO
JQ1
DMSO
JQ1
Input:H4

Fig 6E

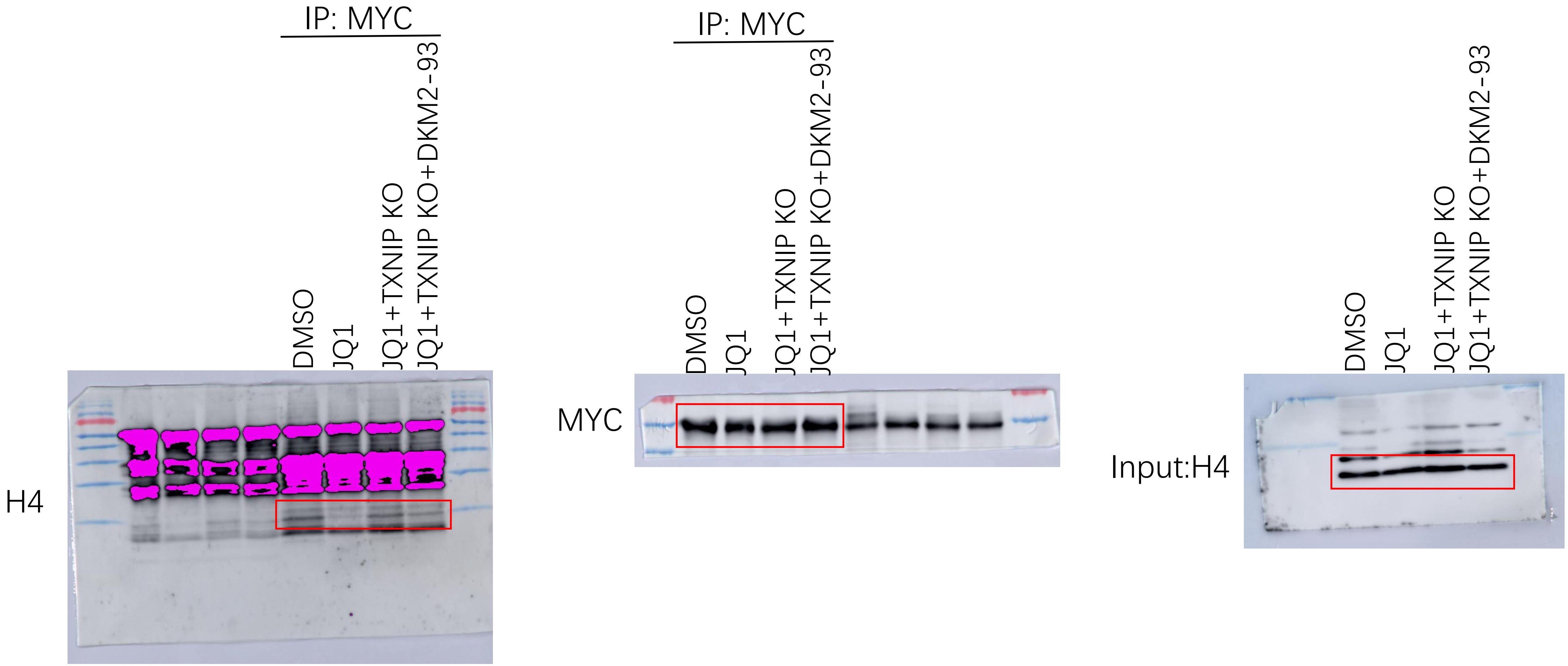
IP: MYC
DMSO
JQ1
JQ1+TXNIP KO
JQ1+TXNIP KO+DKM2-93
H4
IP: MYC
DMSO
JQ1
JQ1+TXNIP KO
JQ1+TXNIP KO+DKM2-93
MYC
DMSO
JQ1
JQ1+TXNIP KO
JQ1+TXNIP KO+DKM2-93
Input:H4

Fig 6G

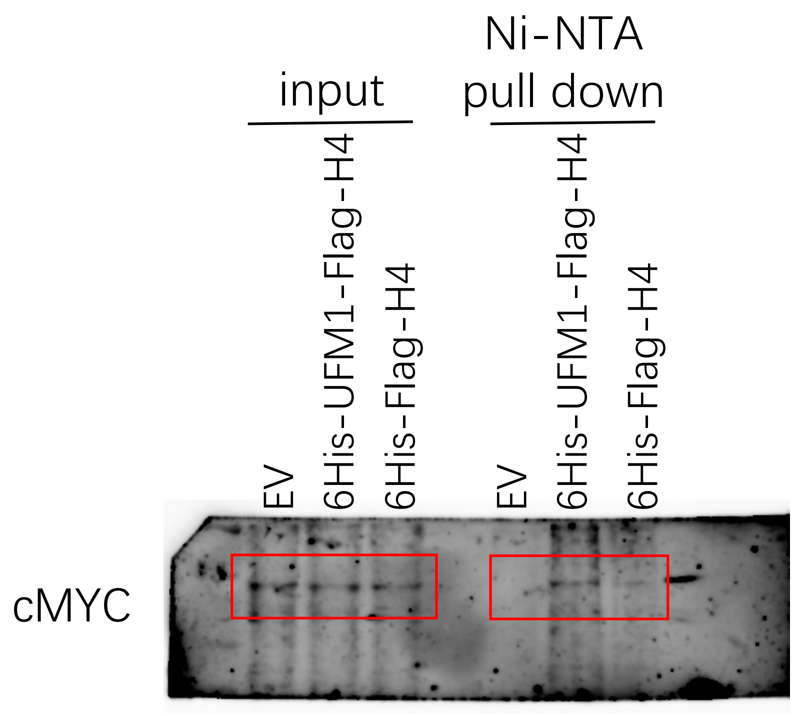
input
Ni-NTA
pull down
EV
6His-UFM1-Flag-H4
6His-Flag-H4
EV
6His-UFM1-Flag-H4
6His-Flag-H4
cMYC

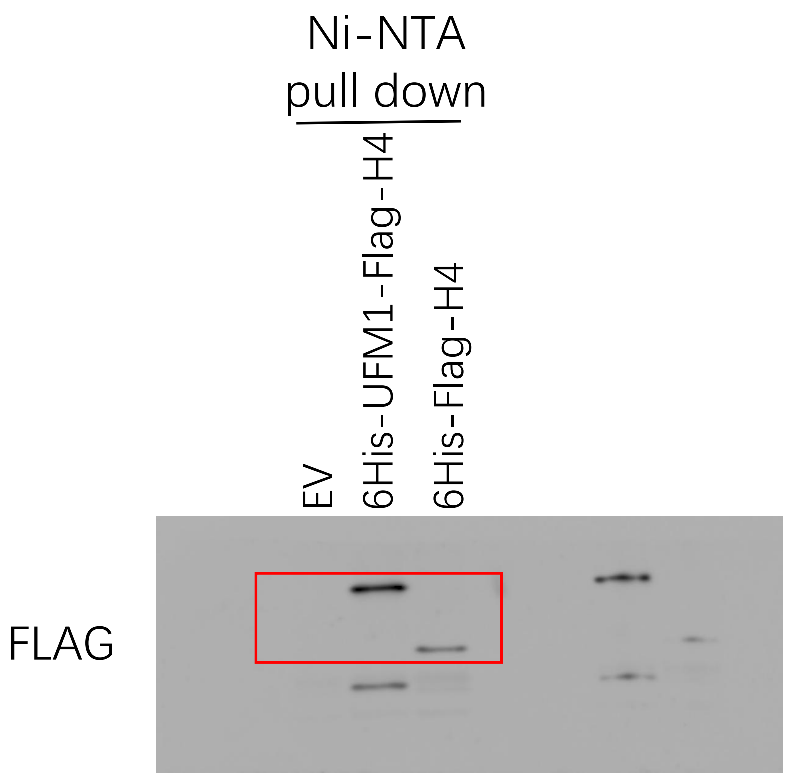
Ni-NTA
pull down
EV
6His-UFM1-Flag-H4
6His-Flag-H4
FLAG

Fig 6H

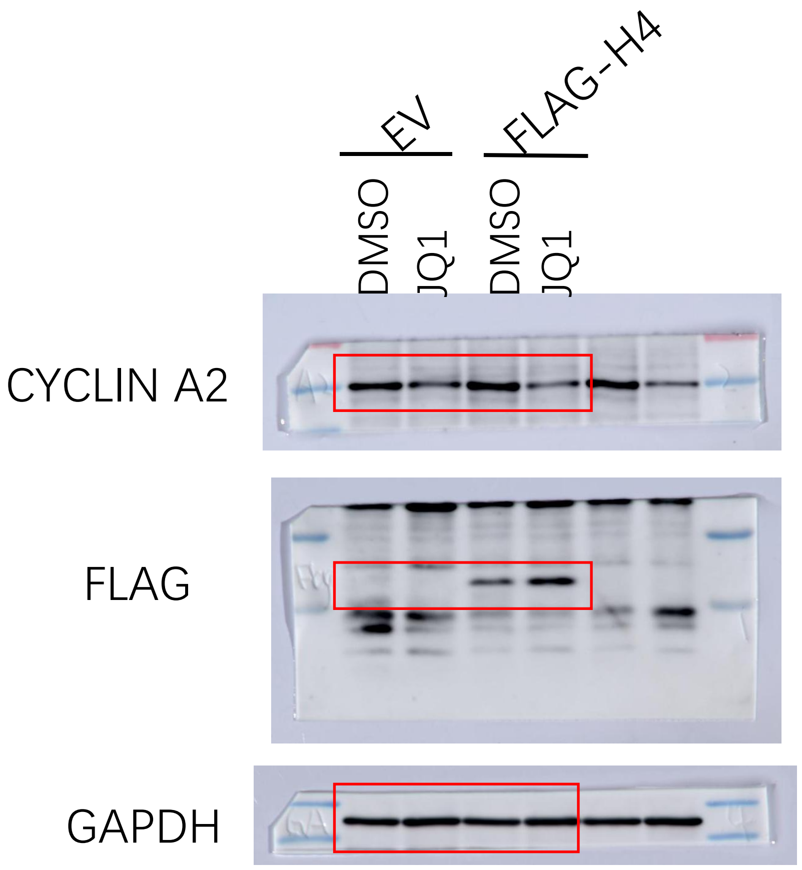
EV
FLAG-H4
DMSO
JQ1
DMSO
JQ1
CYCLIN A2
FLAG
GAPDH

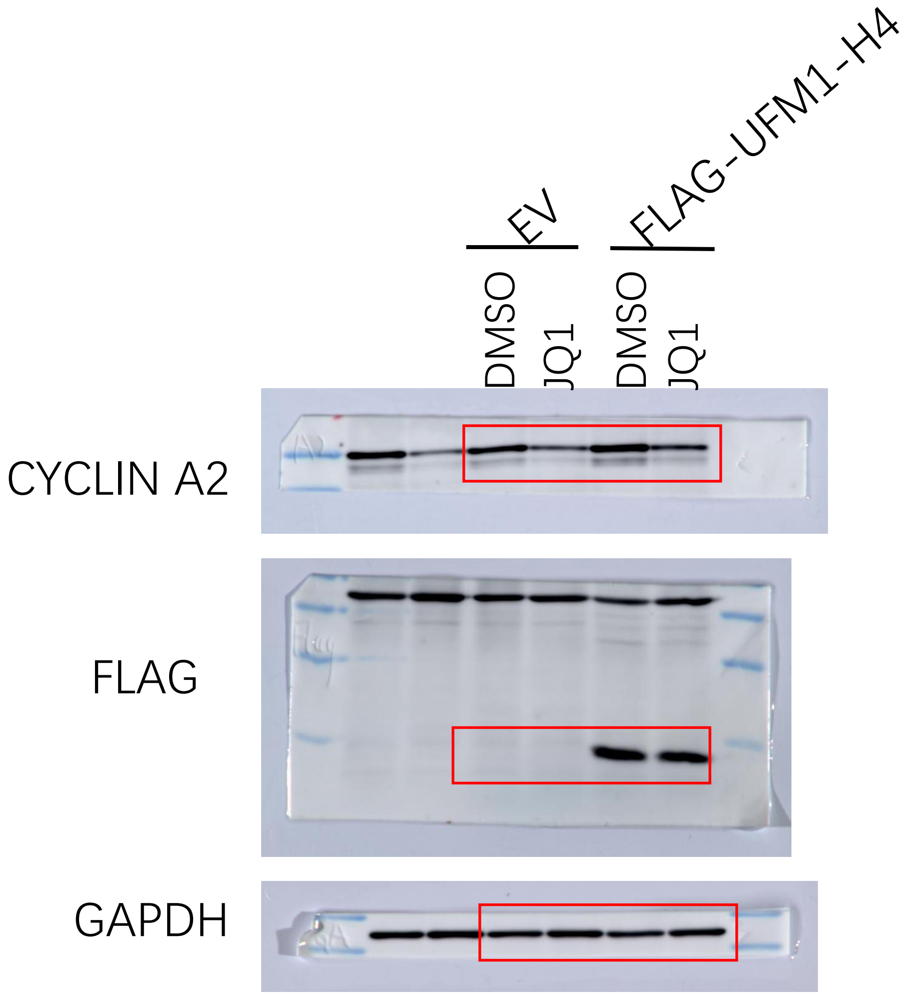
EV
FLAG-UFM1-H4
DMSO
JQ1
DMSO
JQ1
CYCLIN A2
FLAG
GAPDH

Fig 8D

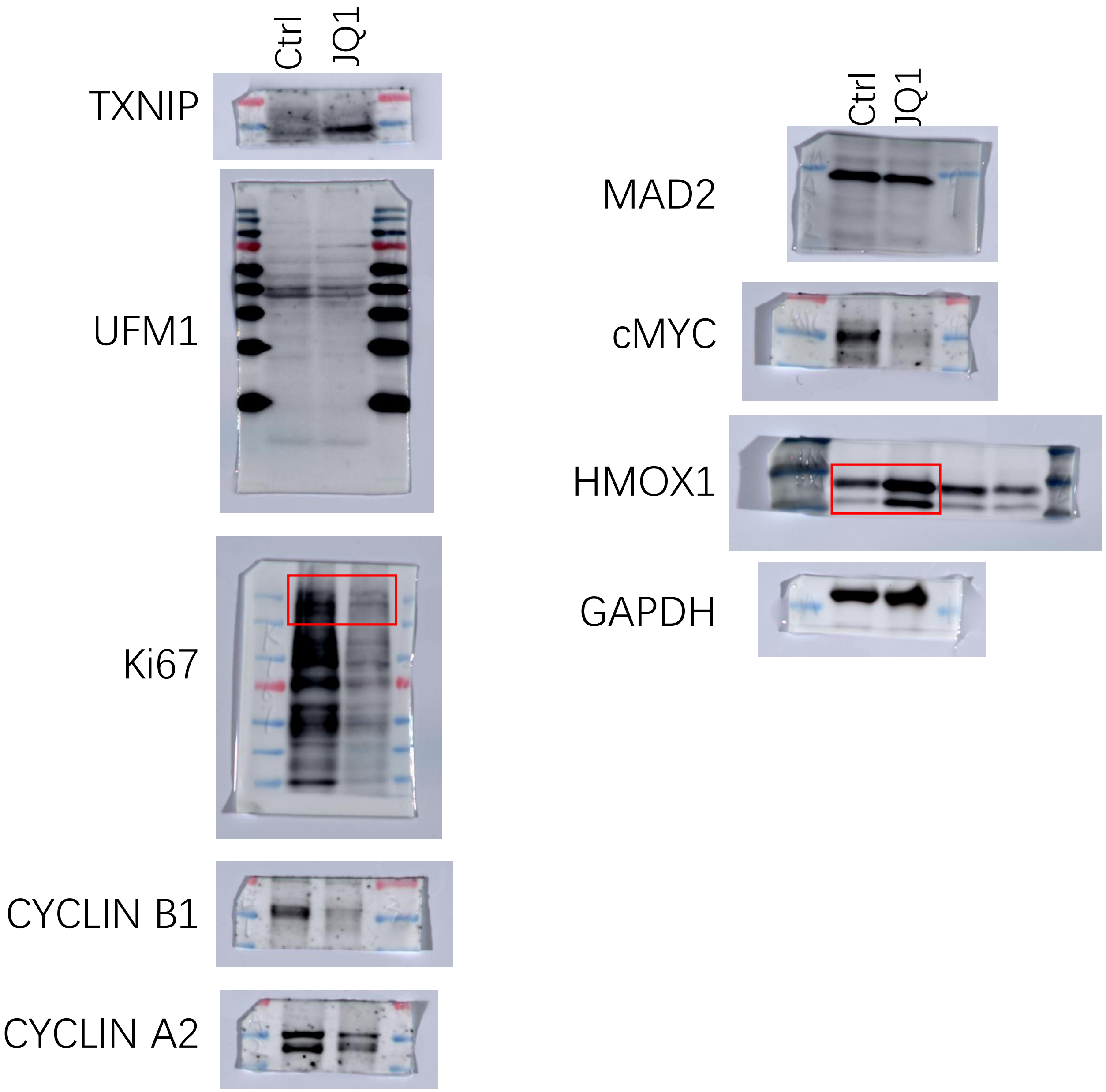
Ctrl
JQ1
TXNIP
UFM1
Ki67
CYCLIN B1
CYCLIN A2
Ctrl
JQ1
MAD2
cMYC
HMOX1
GAPDH

FigS1 F

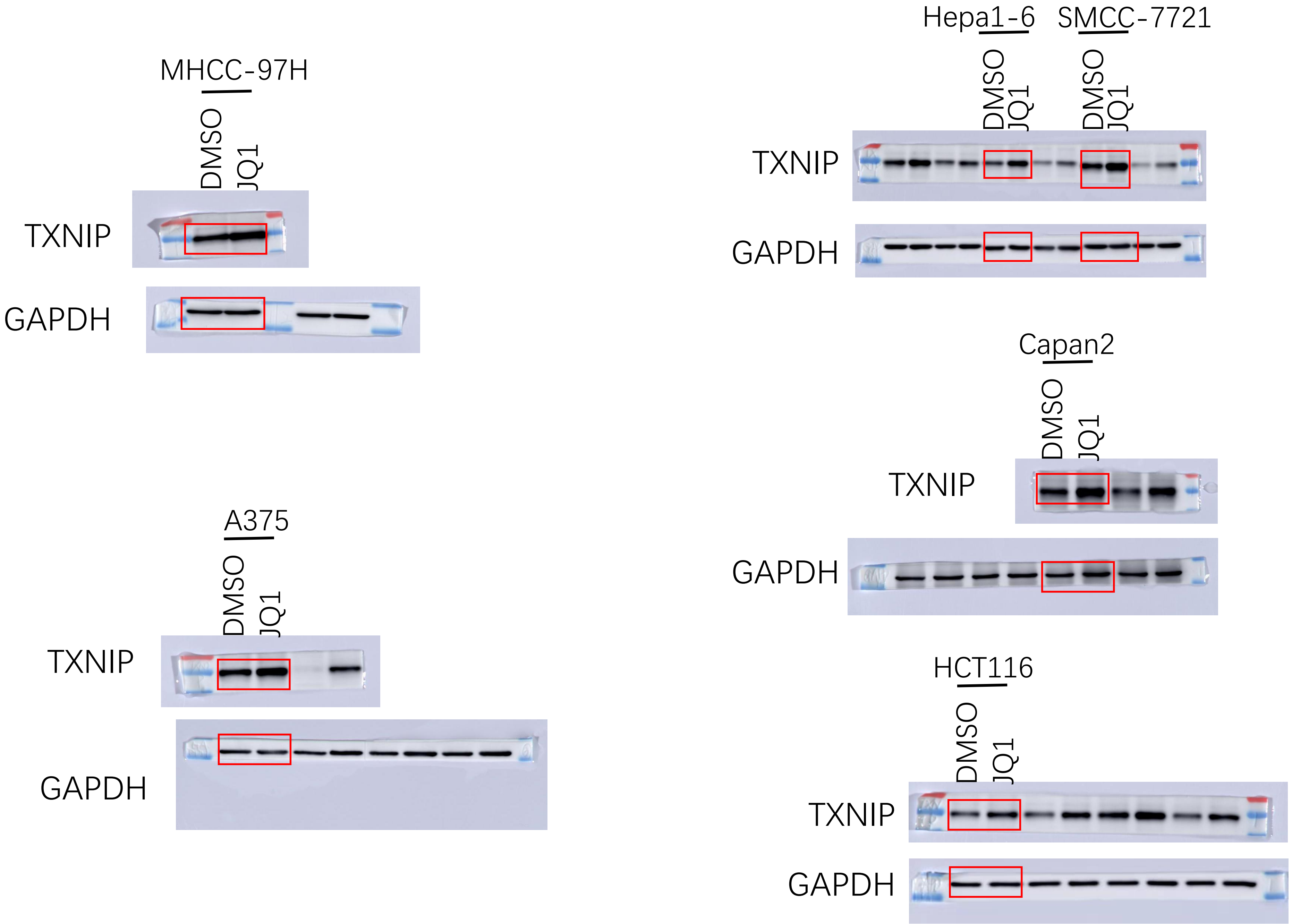
MHCC-97H
DMSO
JQ1
TXNIP
GAPDH
Hepa1-6
SMCC-7721
DMSO
JQ1
DMSO
JQ1
TXNIP
GAPDH
Capan2
DMSO
JQ1
TXNIP
GAPDH
A375
DMSO
JQ1
TXNIP
GAPDH
HCT116
DMSO
JQ1
TXNIP
GAPDH

FigS1 H

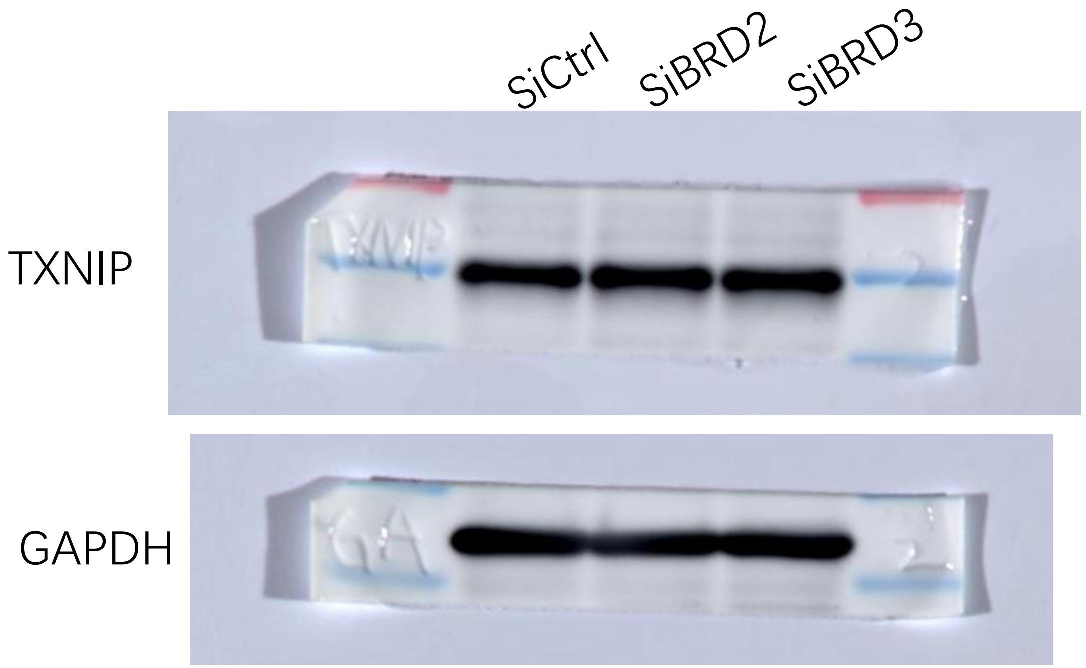

FigS3 A

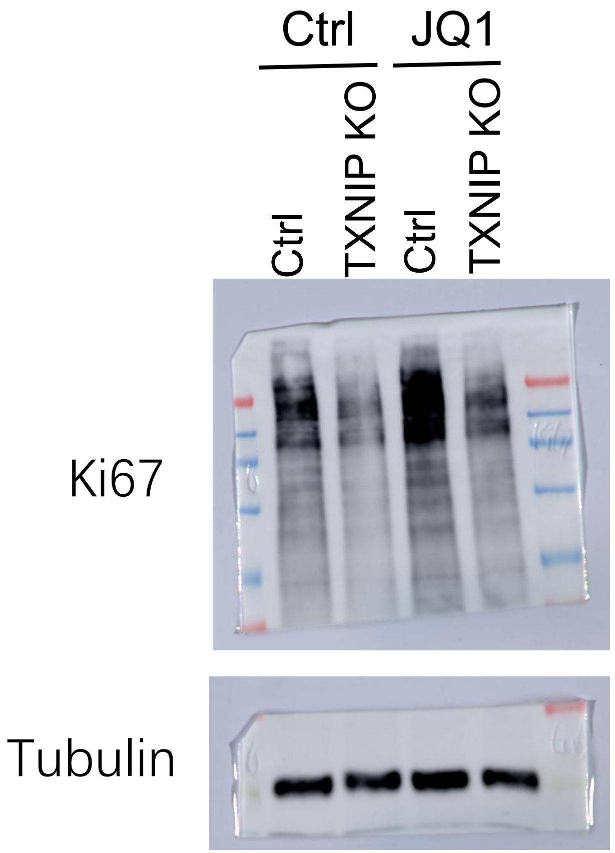

FigS3 F

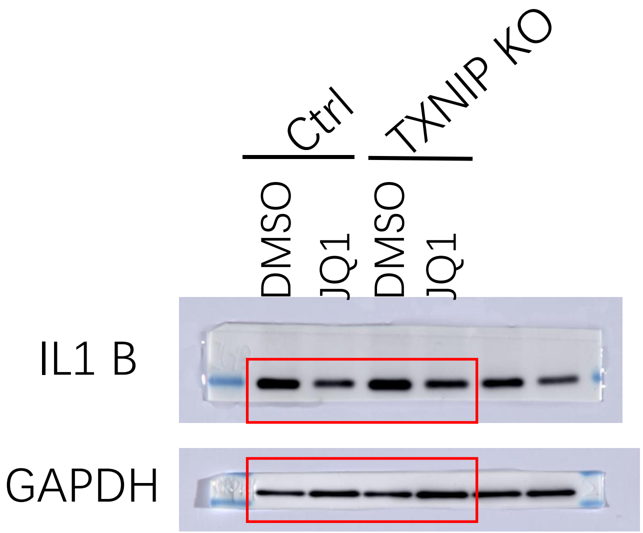

FigS4 A

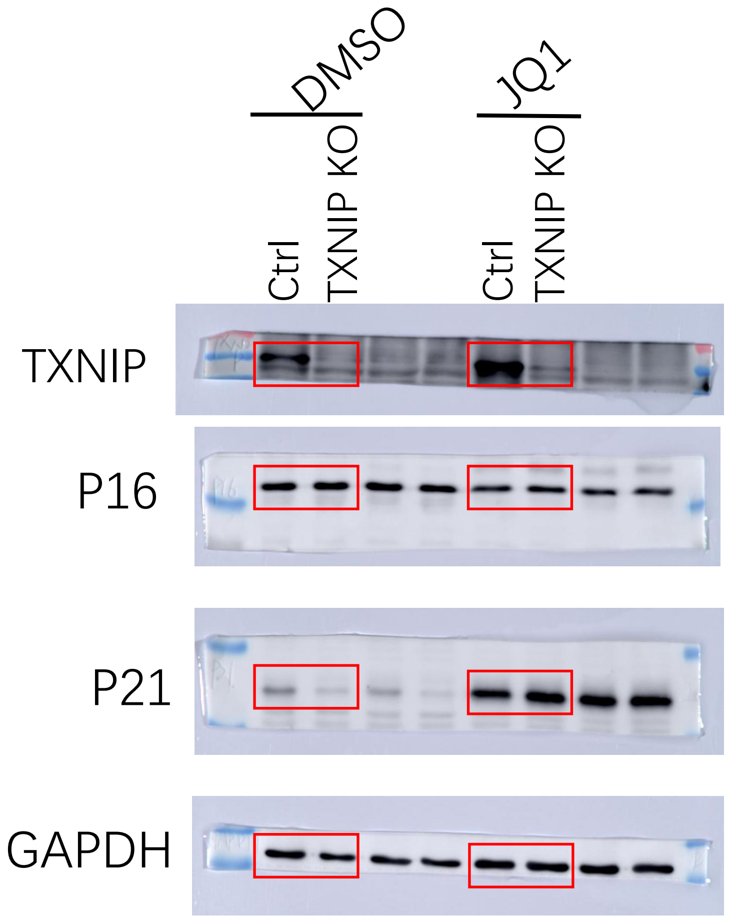

FigS5 D

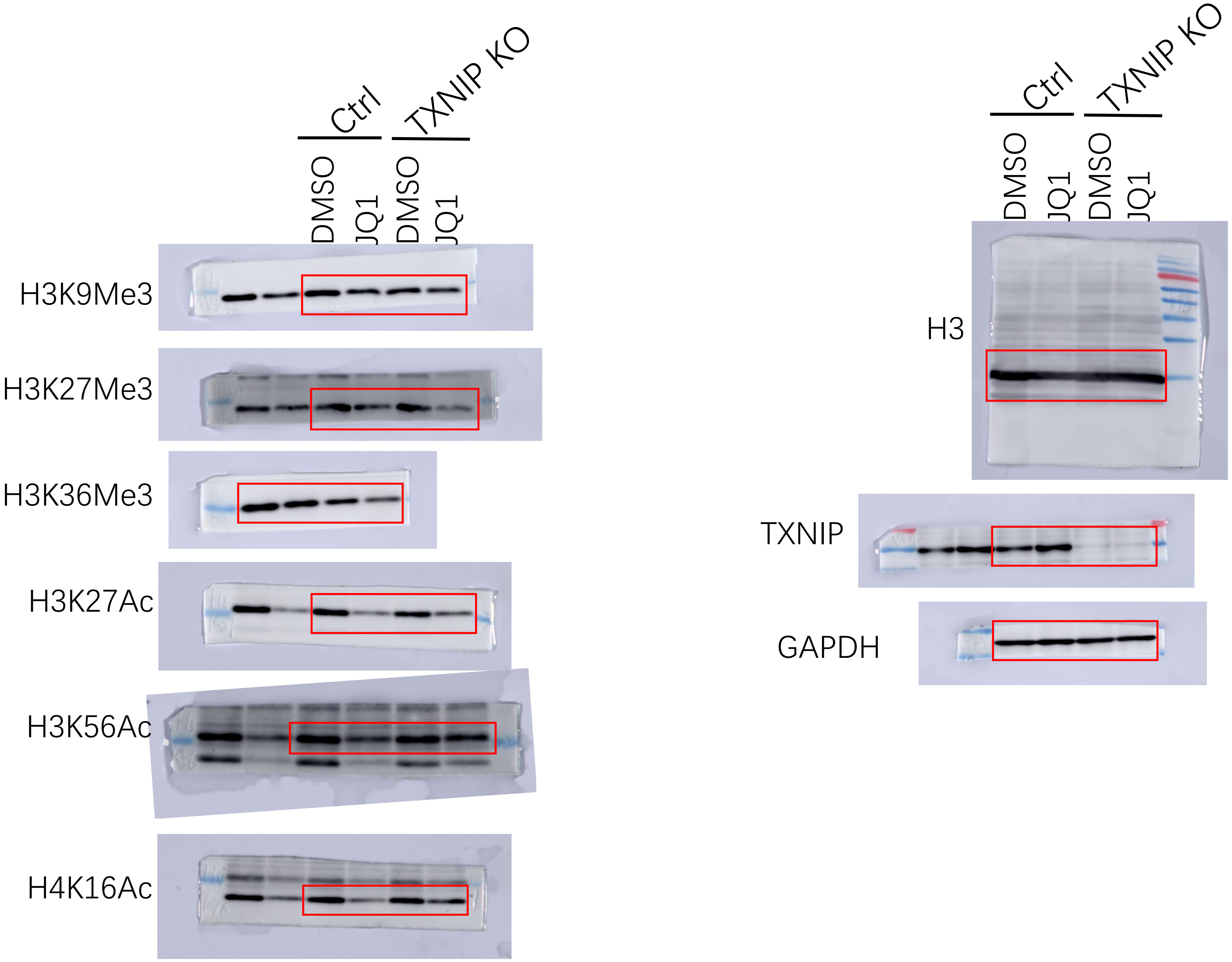
Ctrl
TXNIP KO
DMSO
JQ1
DMSO
JQ1
H3K9Me3
H3K27Me3
H3K36Me3
H3K27Ac
H3K56Ac
H4K16Ac
H3
TXNIP
GAPDH

FigS5 E

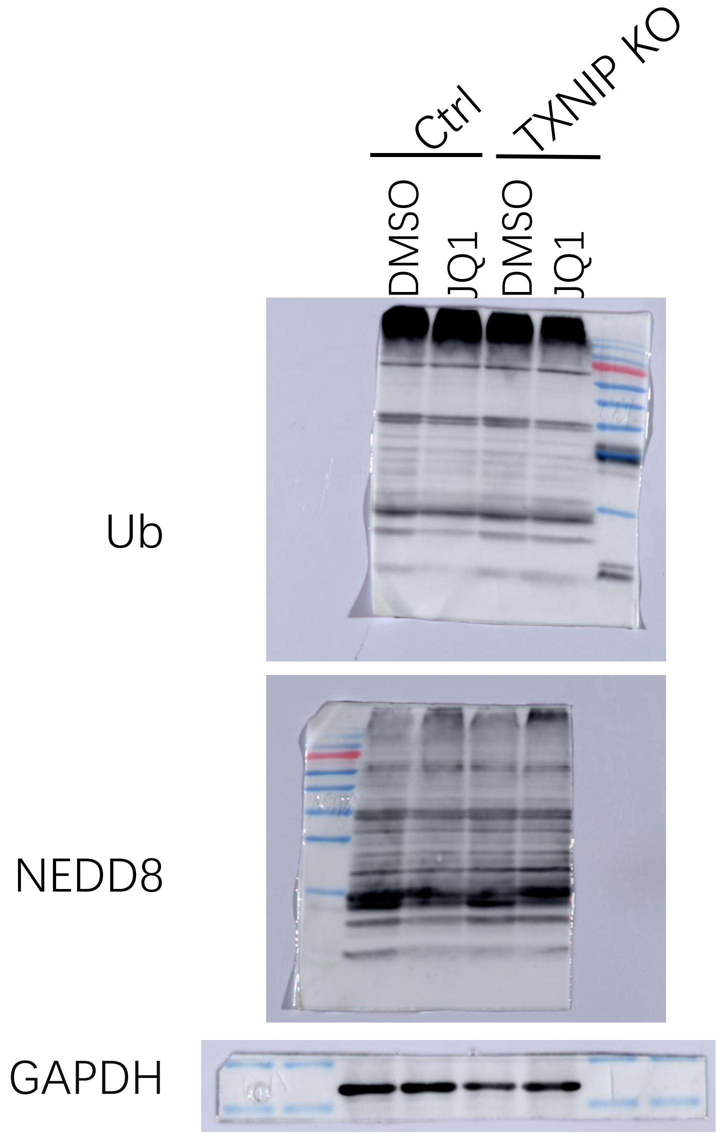

FigS8A

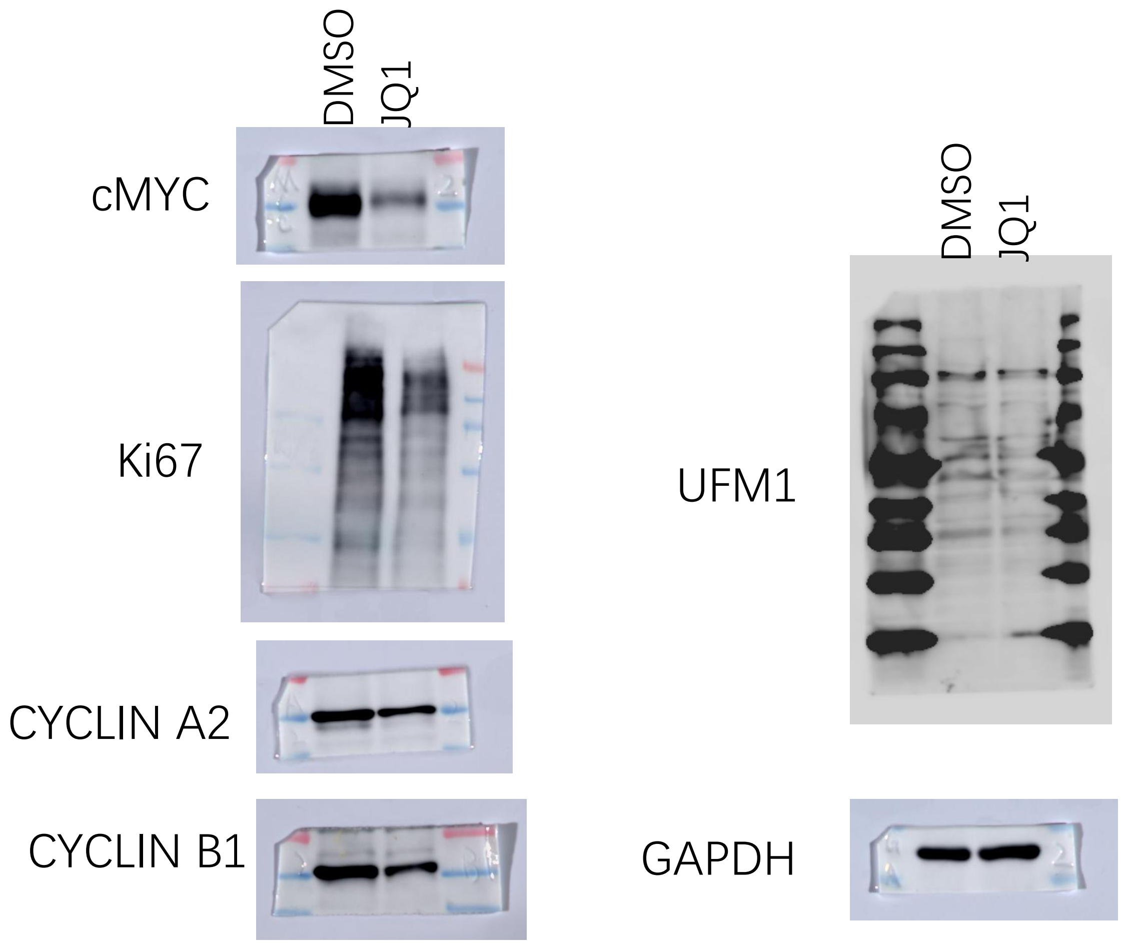

Supplement: Supplementary file 11 — supplemental original WB figures [file 41419_2025_8166_MOESM11_ESM.pdf]
